# Lamination Speeds the Functional Development of Visual Circuits

**DOI:** 10.1016/j.neuron.2015.10.020

**Published:** 2015-12-02

**Authors:** Nikolas Nikolaou, Martin P. Meyer

**Affiliations:** 1 MRC Centre for Developmental Neurobiology, King’s College London, Guy’s Hospital Campus, London, SE1 1UL, UK

## Abstract

A common feature of the brain is the arrangement of synapses in layers. To examine the significance of this organizational feature, we studied the functional development of direction-selective (DS) circuits in the tectum of *astray* mutant zebrafish in which lamination of retinal ganglion cell (RGC) axons is lost. We show that although never laminar, the tuning of DS-RGC axons targeting the mutant tectum is normal. Analysis of mutant tectal neurons at late developmental stages reveals that directional tuning is indistinguishable from wild-type larvae. Furthermore, we show that structural plasticity of tectal dendrites and RGC axons compensates for the loss of lamination, establishing connectivity between DS-RGCs and their normal tectal targets. However, tectal direction selectivity is severely perturbed at earlier developmental stages. Thus, the formation of synaptic laminae is ultimately dispensable for the correct wiring of direction-selective tectal circuits, but it is crucial for the rapid assembly of these networks.

**Video Abstract:**

## Highlights

• Robo2 directs lamination of direction-selective retinal axons and tectal dendrites
• Tectal lamination is required for rapid assembly of direction-selective circuits
• Functional direction-selective circuits eventually form when lamination is lost
• Structural plasticity compensates for the loss of tectal lamination

## Teaser

A feature of the brain is the arrangement of synapses in layers. The significance of this architecture is unknown. Nikolaou and Meyer show that lamination is ultimately dispensable for circuit function but that it is crucial for rapid circuit development.

## Introduction

A common feature of the CNS is the concentration of synapses in layers or laminae. Axons and dendrites are not randomly distributed among different laminae. Instead, a particular lamina will contain axons and dendrites arising from specific neuronal subtypes resulting in the formation of laminae that contain synapses with the same or similar functional properties. For example, the retina and tectum/superior colliculus contain multiple synaptic laminae stacked on top of one another and a lamina’s output will often represent one visual feature such as motion in a particular direction (Baier, 2013, Dhande and Huberman, 2014, Sanes and Zipursky, 2010). While there is now a wealth of experimental evidence showing that the generation of layers can be achieved through multiple cellular and molecular mechanisms (Sanes and Yamagata, 2009), the purpose of layers themselves is not known. One possibility is that laminar-specific targeting of axons and dendrites is a necessary step in the development of synapses between specific neuronal subtypes. In this scenario, laminae are a by-product of the developmental mechanisms that establish wiring specificity in the brain. An alternative idea is that laminae may represent a fundamental unit of brain function, clustering synapses with similar functional properties and segregating synapses whose functional properties differ. This organizing principle may be vital for processing and integration of information in postsynaptic circuits. However, studies of *reeler* mutant mice, in which cortical lamination is lost, show only minor abnormalities in electrophysiological properties of single cortical neurons and sensory map organization and stimulus representation in the barrel and visual cortex are largely intact in these mutants (Dräger, 1981, Guy et al., 2015, Lemmon and Pearlman, 1981, Simmons and Pearlman, 1983). These findings suggest that subdivision of cortical networks into layers may not be required to establish wiring specificity or cortical function. A third explanation for the existence of layered neural architecture is wiring economy, the tendency to minimize axon and dendrite lengths. Wiring minimization has successfully explained the individual placement of neurons in simple nervous systems like that of *C. elegans* and *Drosophila melanogaster*, as well as the spatial organization of coarser structures like cortical areas in the mammalian brain, but it has yet to explain the organization of synaptic connections into layers (Chklovskii and Koulakov, 2004, Pérez-Escudero and de Polavieja, 2007, Rivera-Alba et al., 2011). Thus, there is currently little experimental or theoretical data demonstrating a role for layers in the brain.

Here we use the optic tectum of larval zebrafish to investigate the significance of a layered neural architecture. The tectum is involved in directing the head and eyes to particular locations in visual space and receives input from upward of 20 different RGC subtypes, each responding best to a specific feature in the visual scene. In the zebrafish tectum, RGC axons project to a neuropil composed of four major layers that, from the most superficial to the deepest, are named stratum opticum (SO), stratum fibrosum et griseum superficiale (SFGS), stratum griseum centrale (SGC), and a lamina at the interface between the stratum album centrale and the stratum periventriculare (SAC/SPV) (Burrill and Easter, 1994). The majority of retinal inputs target SFGS, which is further divided into at least six sublaminae (Robles et al., 2013). As a rule, a single RGC axon will only innervate a single sublamina and the laminar specificity with which RGC axons innervate the tectum is precise from the earliest developmental stages (Robles et al., 2013). Functional analysis of direction- and orientation-selective RGCs shows that, at least for these functional classes, precise targeting of axons can generate stereotypic and functionally specialized sublaminae within the tectum. For example, RGCs tuned to upward-, downward-, and forward-directed motion specifically target superficial sublaminae within SFGS (Gabriel et al., 2012, Lowe et al., 2013, Nikolaou et al., 2012). Within the population of tectal neurons are subtypes similarly selective for up, down, and forward motion as well as a subtype selective for backward motion—an emergent property of tectal circuits (Gabriel et al., 2012, Grama and Engert, 2012, Hunter et al., 2013, Niell and Smith, 2005). For forward-selective tectal neurons, tuning can be predicted from the retinal input layer in which their dendrites stratify (Gabriel et al., 2012). Thus, the precise targeting of RGC axons channels information about specific visual features to particular sublaminae and selective sublaminar targeting of tectal dendrites provides a simple mechanism by which retinal information is sampled and integrated by tectal neurons.

How is the functional development of direction-selective (DS) connections between RGCs and tectal neurons affected when lamination of RGC axons is lost? To answer this, we took advantage of the *astray* (*ast*^*ti272z*^) mutant zebrafish in which the laminar arrangement of retinal inputs to the tectum is severely disrupted. The *ast*^*ti272z*^ allele is a functional null mutation of *robo2*—part of an evolutionarily conserved group of transmembrane proteins that, together with their Slit ligands, have been shown to be important for long-range axon guidance, growth, and targeting (Campbell et al., 2007, Fricke et al., 2001, Xiao et al., 2011). Targeting errors of RGC axons within the *ast*^*ti272z*^ tectum are thought to arise from the failure of mutant axons to read a superficial-to-deep gradient of Slit1a (Xiao et al., 2011). The functional consequences of these targeting errors are unknown. Using in vivo functional imaging of RGC axons in the *ast*^*ti272z*^ mutant tectum we show that, although never laminar, the tuning of DS-RGCs is normal. Strikingly, the tuning properties of individual tectal neurons and population encoding of directional motion in the mutant tectum are indistinguishable from that seen in wild-type larvae at late stages of development. Furthermore, we show that structural plasticity of tectal dendrites or RGC axons overcomes the loss of lamination cues, enabling functional and cell-type-specific connections to form between DS-RGCs and their normal tectal neuron targets. However, at early stages of development, direction selectivity in the tectal population is significantly perturbed. Thus, our results show that a laminar tectal architecture is ultimately dispensable for the functional development of direction-selective circuits but that lamination is essential for the rapid assembly of these neural networks.

## Results

### Functional Properties of DS-RGCs Targeting the Tectum Are Normal in *astray* Mutants

**Figure 1:**
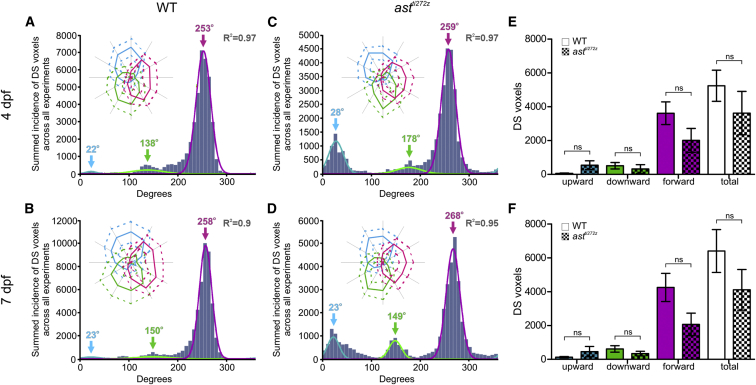
Tuning of DS-RGCs Innervating the Tectum Is Normal in *astray* Mutants Functional imaging in WT (n = 8, total of 24 optical sections) and *ast*^*ti272z*^ (n = 9, total of 27 optical sections) larvae expressing the presynaptic reporter of neural activity, SyGCaMP3, specifically in RGC axons (*Tg(Isl2b:Gal4;UAS:SyGCaMP3)*). (A–D) Cumulative histograms summarizing the incidence of preferred angles of all DS voxels in WT larvae at 4 dpf (A) and 7 dpf (B), and *ast*^*ti272z*^ larvae at 4 dpf (C) and 7 dpf (D). Overlaid colored curves show fitted von-Mises distributions used to identify three functional subtypes of DS-RGC. Color-coded arrows represent peak preferred angles of each subtype. *R*^*2*^ values relate to the summed von-Mises distributions. Insets show polar plots of the mean (solid line) ± 1 SD (dashed line) normalized response profile for voxels of each DS-RGC subtype. (E and F) Quantification of DS voxel numbers shows no significant difference between WT and *ast*^*ti272z*^ RGCs at 4 dpf (E) or 7 dpf (F). All graphs show mean values ± SEM. ns, not significant, unpaired t test. See also Figure S1, Table S1, Movies S1 and S2.

Functional properties of RGCs and tectal neurons have not been studied in the *astray* (*ast*^*ti272z*^) mutant. Therefore, before determining how tectal circuits devoted to processing of motion were altered in animals with disrupted laminar input, we first determined whether direction-selective circuits are established correctly in the mutant retina. We did this by generating wild-type (WT) and *ast*^*ti272z*^ larvae expressing a presynaptically targeted, genetically encoded reporter of neural activity specifically in RGCs (*Tg(Isl2b:Gal4;UAS:SyGCaMP3)*), and recording visually evoked activity in presynaptic terminals of RGC axons within the intact tectum (Nikolaou et al., 2012). Drifting bars moving in each of 12 different directions were presented to one eye of *Tg(Isl2b:Gal4;UAS:SyGCaMP3)* larvae while visually evoked responses were imaged in the contralateral tectum (Movies S1 and S2). To characterize directional tuning in RGC axons, we employed an analysis strategy developed previously that permits direction selectivity to be determined on a voxel-by-voxel basis, but also describes how the direction of motion is encoded on a population basis. The integral response of each voxel during each stimulus epoch was calculated and this was then used to generate tuning curves for each voxel (Figures S1A–S1F). Direction- and orientation-selective indices (DSI and OSI) of all visually responsive voxels were calculated based on fitted von-Mises profiles, together with an estimate for goodness of fit, R^2^. For a voxel to be regarded as DS, mutually exclusive criteria were employed: DS if DSI > 0.5, OSI < 0.5, and the *R*^*2*^ for DSI and OSI had to be greater than 0.8 (Lowe et al., 2013) (see Experimental Procedures). The center of the fitted curves used to estimate DSI was also used to provide an estimate of the preferred direction of motion. The WT and *ast*^*ti272z*^ larvae were imaged at two stages of development—4 days post-fertilization (4 dpf), which is approximately 24 hr after the first RGC axons enter the tectum, and 7 dpf when synapses between RGC axons and tectal dendrites and visually evoked behaviors are well established (Fleisch and Neuhauss, 2006, Meyer and Smith, 2006, Niell et al., 2004). The preferred angles of DS voxels from all imaged larvae were used to generate a population histogram for each condition (WT and *ast*^*ti272z*^ larvae imaged at 4 and 7 dpf). These reveal distinct distributions of preferred angles that are strikingly similar in WT and mutant tecta (Figures 1A–1D). Moreover, by iteratively fitting three summed von-Mises distributions to the population histograms (overlaid color curves in Figures 1A–1D), we found that there are three, largely non-overlapping, DS subpopulations with peaks centered at very similar locations in WT and mutant larvae. Location of peak centers across the four experimental conditions ranged from 22°–28° (upward motion), 138°–178° (downward motion), and 253°–268° (forward motion) (color-coded arrows in Figures 1A–1D). To estimate the relative proportions of each DS functional subtype, we band-limited responsive voxels to those within two times the bandwidth of each fitted von-Mises distribution. We found that the number of voxels of each DS subtype and the total number of DS voxels are not significantly different in *ast*^*ti272z*^ and WT larvae at either 4 or 7 dpf (Figures 1E and 1F). To test whether the response properties of DS subtypes differ between the two experimental groups, we generated polar plots illustrating the mean (±1 SD) normalized response profile for each DS subtype (insets in Figures 1A–1D). These illustrate that in both WT and *ast*^*ti272z*^ larvae, the three DS-RGC subtypes fill direction space and exhibit a triangular organization. Furthermore, we found that in both *ast*^*ti272z*^ and WT tecta DS-RGC tuning is precise from 4 dpf and that tuning bandwidths of DS-RGC subtypes are very similar in WT and *ast*^*ti272z*^ larvae (Table S1). These data show that in WT larvae the tuning and development of DS-RGCs are the same as that described previously (Lowe et al., 2013, Nikolaou et al., 2012) and that the functional development of DS-RGCs is normal in *ast*^*ti272z*^ mutants.

### Lamination of DS-RGCs Is Perturbed in the *astray* Mutant Tectum

**Figure 2:**
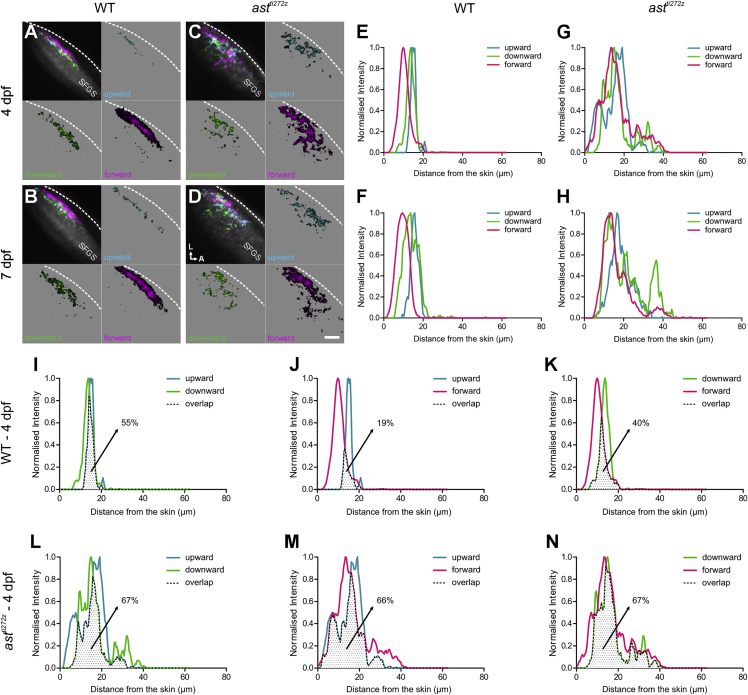
Lamination of DS-RGC Axons in the Tectum Is Lost in *astray* Mutants (A–D) Composite parametric maps generated from all *Tg(Isl2b:Gal4;UAS:SyGCaMP3)* fish imaged showing the spatial distribution of DS-RGC subtypes in the tectal neuropil of WT larvae (n = 8, total of 24 optical sections) at 4 dpf (A) and 7 dpf (B), and *ast*^*ti272z*^ larvae (n = 9, total of 27 optical sections) at 4 dpf (C) and 7 dpf (D). Voxel brightness is proportional to the summed incidence of each functional subtype across all larvae imaged. The standard space template image derived from the mean fluorescence image of SyGCaMP3-expressing axons (grayscale) provides an anatomical reference. Dashed lines indicate the position of the skin overlaying the tectum. Scale bar represents 20 μm. A, anterior; L, lateral; SFGS, stratum fibrosum et griseum superficiale. (E–H) Line plots generated from the composite parametric maps in (A)–(D) illustrating the distribution of DS-RGC subtypes along the laminar axis of the tectum in WT larvae at 4 dpf (E) and 7 dpf (F), and *ast*^*ti272z*^ larvae at 4 dpf (G) and 7 dpf (H). Line plots show normalized intensity of each DS-RGC subtype as a function of its distance from the skin. (I–N) Pairwise comparisons showing the degree of spatial overlap between upward and downward (I and L), upward and forward (J and M), and downward and forward (K and N) DS-RGC subtypes within WT (I–K) and *ast*^*ti272z*^ (L–N) tecta at 4 dpf. Dotted area represents the area of intersection between the two subtypes. Values shown represent the fraction of downward (for I and L) or forward (for J, K, M, and N) DS voxels that spatially overlap with upward or downward DS voxels. See also Figures S2 and S3.

To examine how the targeting of axons of DS-RGCs is altered in the *ast*^*ti272z*^ mutant, we spatially co-registered data from all imaged larvae in each experimental condition to generate composite functional maps of DS-RGC axons in WT and mutant tecta (see Experimental Procedures). The panels in Figures 2A–2D are color coded for the three DS-RGC subtypes and in each parametric map, voxel brightness is proportional to the summed incidence of each functional subtype across all larvae. In WT animals, responses of DS-RGC subtypes are confined to superficial sublaminae within SGFS throughout development (Figures 2A and 2B) (Lowe et al., 2013, Nikolaou et al., 2012). In contrast, we found that the laminar functional architecture of DS-RGC subtypes in *ast*^*ti272z*^ larvae is severely disrupted at both 4 and 7 dpf, as would be expected from previous morphological studies of RGC axons in the *ast*^*ti272z*^ retinotectal projection (Xiao et al., 2011). Specifically, responses of DS-RGC subtypes are diffusely distributed across SFGS in *ast*^*ti272z*^ mutants (Figures 2C and 2D), a pattern that is also evident in functional maps generated from single larvae (compare Figures S2A and S2B with S2C and S2D). To further examine the distribution of DS-RGC responses in the mutant tectum, we used the composite maps to generate line plots showing the summed incidence of each functional subtype across an axis that represents the laminar organization of the tectal neuropil. In WT animals, the three DS-RGC subtypes at each age exhibit a sublaminar organization with the most abundant subtype (forward motion, magenta) found in a superficial sublamina, whilst the subtypes selective for upward and downward motion occupy a slightly deeper sublamina (Figures 2E and 2F) (Lowe et al., 2013, Nikolaou et al., 2012). In contrast, the laminar organization of DS-RGC subtypes in *ast*^*ti272z*^ animals is lost (Figures 2G and 2H). While lamination may be lost, the relative ordering of DS-RGC subtypes across the depth of the tectum may still be intact. To examine this, we performed pairwise comparisons of the spatial distributions of each subtype within WT and *ast*^*ti272z*^ larvae at 4 dpf (Figures 2I–2N) and 7 dpf (Figures S3G–S3L). This reveals that the degree of overlap between subtypes is increased in the mutant and that the relative order of functional subtypes across the laminar axis of the tectum is perturbed in the mutants. For example, in wild-type tecta, voxels tuned to forward motion always occupy the most superficial locations at both 4 and 7 dpf. In the mutant tectum, voxels tuned to upward motion invade the territory normally only occupied by voxels tuned to forward motion (compare Figure 2J with 2M and Figure S3H with S3K). Similarly, voxels tuned to upward and downward occupy the deepest locations in wild-type larvae but in the mutant voxels selective for forward motion invade these locations (for example, compare Figure S3I with S3L). Thus, lamination and the relative ordering of DS-RGC subtypes are perturbed in the mutant. However, a bias toward the superficial half of SFGS is nevertheless maintained in the mutant tectum, suggesting mechanisms that establish a coarse organization of retinal afferents are present in the *ast*^*ti272z*^ tectum. By overlaying the line plots for each DS subtype from WT and *ast*^*ti272z*^ larvae, we estimated from the area of intersection the fraction of all DS voxels that are located in the correct location in mutant tecta. At 4 dpf, this fraction is 24% (upward motion), 43% (downward motion), and 38% (forward motion). At 7 dpf, the fraction is 44% (upward motion), 54% (downward motion), and 46% (forward motion) (Figures S3A–S3F). These data show that while the functional properties of DS-RGCs targeting the tectum are normal in *ast*^*ti272z*^ mutants, the laminar architecture of these inputs is absent at both 4 and 7 dpf.

### Development of DS Tectal Cell Responses Is Delayed in *astray* Mutants

**Figure 3:**
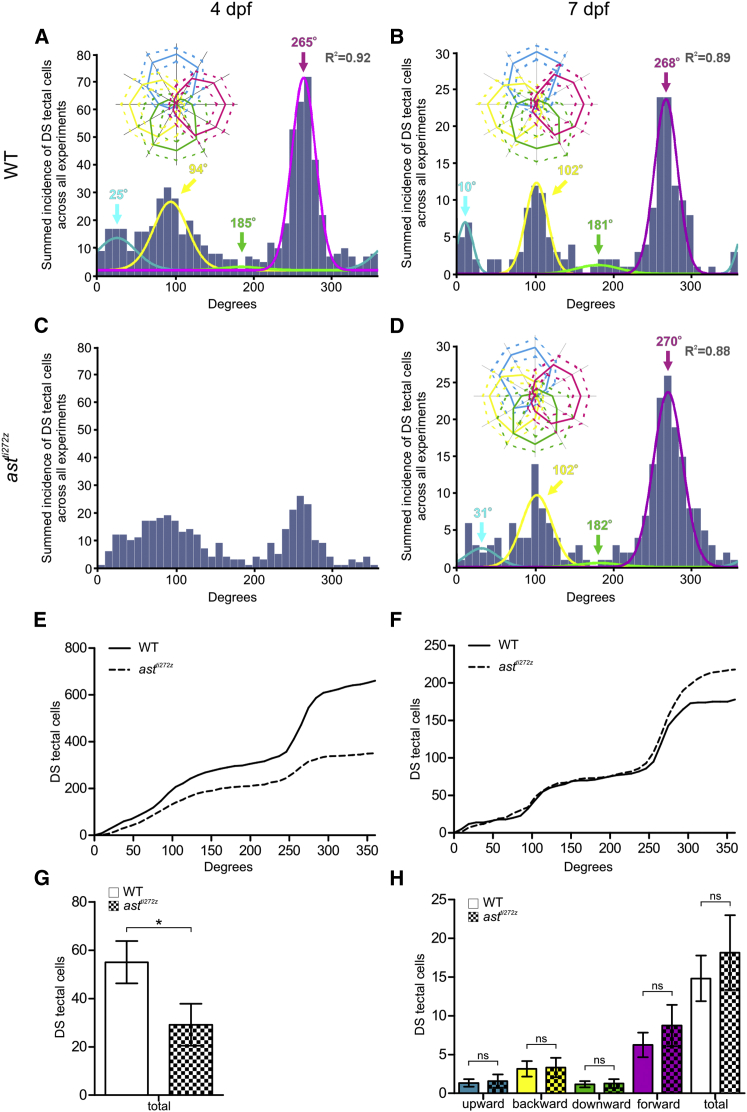
Functional Development of DS Tectal Cells Is Delayed in *astray* Mutants Functional imaging of tectal cell responses in WT (n = 12, total of 36 optical sections) and *ast*^*ti272z*^ (n = 12, total of 36 optical sections) larvae with pan-neuronal expression of GCaMP5G (*Tg(elavl3:GCaMP5G)*). (A–D) Cumulative histograms summarizing the incidence of preferred angles for DS tectal cells in WT larvae at 4 dpf (A) and 7 dpf (B), and *ast*^*ti272z*^ larvae at 4 dpf (C) and 7 dpf (D). Overlaid colored curves in (A), (B), and (D) show fitted von-Mises distributions used to identify the four DS subtypes and the color-coded arrows represent peak preferred angles. *R*^*2*^ values relate to the summed von-Mises distributions. Insets in (A), (B), and (D) show polar plots of the mean (solid line) ± 1 SD (dashed line) normalized responses of cells within each DS subtype. Note that the same functional subtypes are present at 4 and 7 dpf in WT tecta. (E and F) Cumulative plots generated from the data in (A)–(D) comparing the incidence of preferred angles for DS tectal cells between WT and *ast*^*ti272z*^ larvae at 4 dpf (E) and 7 dpf (F). (G and H) Quantification of DS tectal cell number at 4 dpf (G) and 7 dpf (H). Note the significant reduction in DS tectal cell numbers at 4 dpf, but recovery at 7 dpf. All graphs show mean values ± SEM. ^∗^p < 0.05; ns, not significant, unpaired t test. See also Figures S4 and S5, Table S2, Movies S3 and S4.

Our next goal was to determine how, if at all, spatial disorder in the retinal inputs to the tectum impacts the functional development of DS tectal neurons. We accomplished this by performing functional imaging of tectal neurons in *ast*^*ti272z*^ mutants expressing GCaMP5G in the majority of neurons in the brain *(Tg(elavl3:GCaMP5G)* (Ahrens et al., 2013) (Movies S3 and S4). DS-tectal neurons were identified by functional imaging at 4 dpf and 7 dpf using the procedure for RGCs described above (Figures S4A–S4F). DS metrics (DSI > 0.5, OSI < 0.5 and *R*^*2*^ > 0.8) were applied to all visually responsive voxels followed by automatic aggregation of similarly tuned voxels into cell body-sized units (see Experimental Procedures). The resultant preferred angles of all DS tectal cells were summed across all subjects within each of the four experimental groups (WT and *ast*^*ti272z*^ larvae imaged at 4 and 7 dpf). Surprisingly, cumulative histograms reveal almost identical distributions in the preferred angles of tectal cells in WT and *ast*^*ti272z*^ larvae at 7 dpf (Figures 3B, 3D, and 3F). These distributions were fit by four summed von-Mises distributions (overlaid color curves in Figures 3B and 3D), demonstrating that the four DS subpopulations of tectal cells identified previously (tuned to upward, backward, downward, and forward motion) (Hunter et al., 2013) are present in both WT and *ast*^*ti272z*^ tecta. Furthermore, we found no significant difference in the number of DS tectal cells in each subtype or in the total number of DS tectal neurons between WT and *ast*^*ti272z*^ larvae (Figure 3H). (The decrease in DS-tectal neurons seen in WT larvae reflects the progressive loss of GCaMP5G expression rather than a real reduction in DS tectal cell numbers, which have been shown to be constant during development [Niell and Smith, 2005].) We also found that tuning angle and bandwidths of DS tectal neurons were not significantly altered in mutant larvae (insets in Figures 3B and 3D; Table S2). These results demonstrate that despite the loss of laminated retinal input, all DS tectal neuron subtypes, including the emergent population tuned to backward motion, are established in appropriate numbers by 7 dpf. However, functional imaging of the same animals at 4 dpf reveals a marked reduction in the numbers of DS tectal cells across all preferred angles (Figures 3A, 3C, and 3E), resulting in a significant reduction in the total number of DS tectal neurons in the tectum of *ast*^*ti272z*^ mutants at 4 dpf (Figure 3G; WT: 55 ± 8.7, *ast*^*ti272z*^: 29.1 ± 8.6, p < 0.05). (The four normally distributed populations are not as clearly evident in the cumulative data from 4 dpf *ast*^*ti272z*^ mutants, precluding the possibility of identifying the four functional subtypes at this stage.) The reduction in DS tectal cell number is not due to delayed proliferation or differentiation, because the numbers of mitotic (PH-3^+^) and postmitotic (HuC/D^+^) cells in the developing *ast*^*ti272z*^ tectum at 48 hpf are comparable to those found in WT animals (Figures S5A–S5D). The reduced number of DS tectal neurons is also unlikely to result from a failure to form retinotectal synapses per se, since the number of presynaptic sites in RGC axons is actually increased in *ast*^*ti272z*^ mutants (Campbell et al., 2007). These results show that loss of a laminar architecture results in a significant delay in the functional development of DS circuits in *ast*^*ti272z*^ mutant tecta.

### Lamination of DS-Tectal Cell Dendrites Is Lost in *astray* Mutants

**Figure 4:**
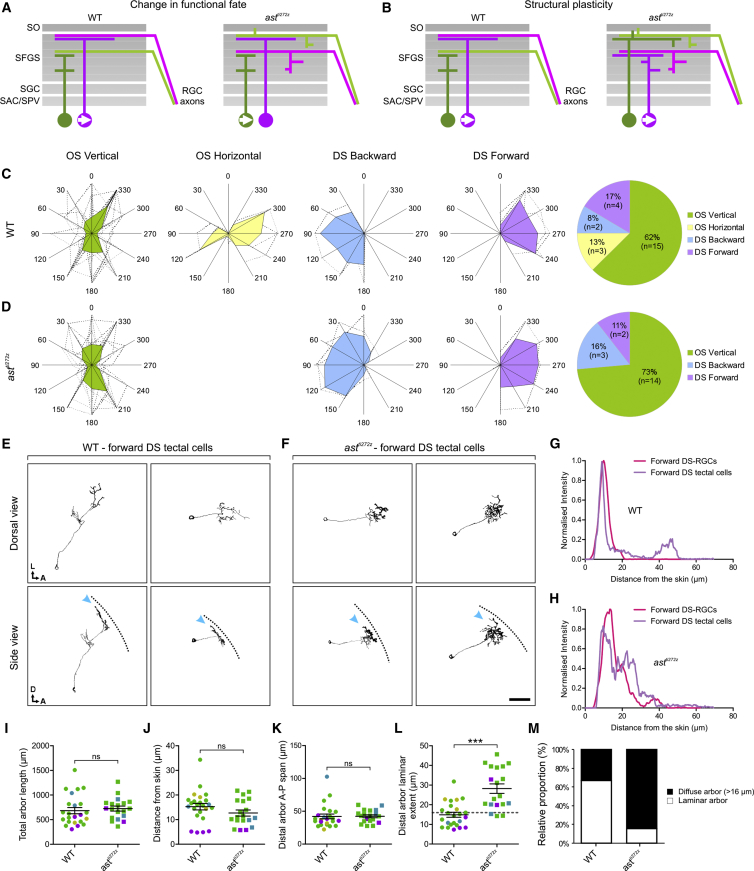
Morphology of DS Tectal Cell Dendrites Is Disrupted in the *astray* Mutant (A and B) Two alternative models of how direction selectivity recovers in the tectal cell population in *ast*^*ti272z*^ mutants, functional plasticity (A) or structural plasticity (B). SO, stratum opticum; SFGS, stratum fibrosum et griseum superficiale; SGC, stratum griseum centrale; SAC, stratum album centrale; SPV, stratum periventriculare. (C and D) Polar plots illustrating functional subtypes of WT (n = 24 in 22 larvae) (C) and *ast*^*ti272z*^ (n = 19 in 16 larvae) (D) tectal cells singly labeled with GCaMP6F in the 7 dpf tectum following co-electroporations of *FoxP2.A:Gal4FF* + *5UAS:GCaMP6F* DNA constructs. Dashed lines indicate polar plots from individual cells, and filled color-coded polar plots represent the mean for each functional subtype. Pie charts to the right indicate the relative proportions of functional subtypes and number of neurons analyzed. (E and F) Morphologies of tectal cells selective for forward motion. Two examples from WT (E) and *ast*^*ti272z*^ tecta (F) are shown. Note that the laminar profile of DS tectal cells tuned to forward motion is lost in *ast*^*ti272z*^ animals (arrowheads). Dashed lines indicate the position of the skin overlaying the tectum. Scale bar represents 30 μm. A, anterior; D, dorsal; L, lateral. (G and H) Line plots comparing the distribution of forward tuned DS-RGC axons and forward tuned DS tectal cell dendrites along the laminar axis of the tectum at 7 dpf in WT (G) and *ast*^*ti272z*^ (H) animals. Line plots show normalized intensity of each cell type as a function of its distance from the skin. Note the alignment of DS-RGC axons and DS tectal dendrites in both conditions. (I–L) Comparison of four morphological parameters extracted from all tectal cells (Figure S4A). For total arbor length (I), 682.6 ± 62.4 for WT and 724.6 ± 50.5 for *ast*^*ti272z*^; for distance from skin (J), 15.2 ± 1.3 for WT and 12.6 ± 1.2 for *ast*^*ti272z*^; for distal arbor anterior-posterior span (K), 42 ± 3.6 for WT and 42.3 ± 2.2 for *ast*^*ti272z*^; and for distal arbor laminar extent (L), 14.9 ± 1.2 for WT and 28.2 ± 2.4 for *ast*^*ti272z*^. Data points are color coded according to functional subtype as in (C) and (D). All graphs show mean values ± SEM. ^∗∗∗^p < 0.001; ns, not significant, Mann Whitney test. (M) Proportions of cells with laminar and diffuse (distal arbor laminar extent < 16 μm and > 16 μm, respectively) arbors in WT and *ast*^*ti272z*^ tecta. WT: 67% laminar, 33% diffuse arbor; and *ast*^*ti272z*^: 16% laminar, 84% diffuse arbor. See also Figures S6 and S7 and Movies S5 and S6.

The recovery of tectal direction selectivity at 7 dpf in *ast*^*ti272z*^ mutants demonstrates a striking ability of the tectum to adapt to the loss of laminated retinal input. We reasoned that two mechanisms could account for functional recovery: (1) a change in functional fate, in which tectal cells that are not fated to become DS maintain their morphology in the mutant tectum but form synapses indiscriminately with the misplaced DS-RGC axons and, in so doing, adopt directional tuning (Figure 4A). Such a scenario would imply that lamination cues such as Robo2 are necessary for wiring specificity. (2) Structural plasticity, in which DS-tectal cells adjust their dendritic morphology in order to locate and synapse with the misplaced DS-RGCs. This would imply that cell-type-specific wiring is retained in the mutant and that structural plasticity in the developing brain enables functional and cell-type-specific connections to form despite the loss of lamination cues (Figure 4B). To distinguish between these mechanisms, it was necessary to label tectal cells whose morphology and function is predictable so that we could determine which is changing in the mutant. Here we identify the *FoxP2.A* enhancer as a means to genetically target subtypes of tectal cells (Bonkowsky et al., 2008). We used the *FoxP2.A* enhancer to drive expression of Gal4 and co-electroporated *FoxP2.A:Gal4FF* and *5UAS:GCaMP6F* plasmids into the tectum to label isolated tectal cells with the genetically encoded reporter of neural activity, GCaMP6F (Chen et al., 2013). This allowed us to examine both the tuning and morphology of single tectal cells in the WT and mutant tectum at 7 dpf (an example of a tuning experiment in single cell is shown in Movie S5). We found that tectal cells labeled by this approach could be grouped into four functional subtypes: 62% of labeled cells were selective for horizontally oriented bars moving along the vertical axis (vertically tuned), 13% were selective for vertically oriented bars moving along the horizontal axis (horizontally tuned), 8% were backward DS, and 17% were forward DS (Figure 4C; also Figures S6A–S6F for response profiles of individual FoxP2.A-labeled cells). Morphological reconstruction of these cell types revealed that they are morphologically diverse (Figures S7B and S7C) but, consistent with a previous study (Gabriel et al., 2012), we find that WT DS tectal cells tuned to forward motion have laminar dendrites (arrowheads in Figure 4E; Movie S6) that co-stratify with the axons of DS-RGCs in superficial sublaminae within SFGS (Figure 4G). Three of these subtypes were also labeled by the *FoxP2.A:Gal4FF* construct in the tectum of *ast*^*ti272z*^ mutants and in similar proportions to that seen in WT tecta (Figure 4D; also Figures S6G–S6L for response profiles of individual FoxP2.A-labeled cells). The exception was an absence of horizontally tuned cells—one of the cell types rarely labeled in WT larvae. The fact that the *FoxP2.A* enhancer labels similar functional subtypes of tectal neuron in the WT and mutant tectum suggests that a change in functional fate of tectal neurons is unlikely to account for the recovery of tectal direction selectivity in *ast*^*ti272z*^ mutants. If this were the case, we would expect to see a change either in tuning or in the proportions of functional subtypes targeted by the *FoxP2.A* enhancer. To examine whether structural plasticity could account for the functional recovery of direction selectivity in the tectum, we reconstructed the morphology of tectal neurons labeled by the *FoxP2.A* enhancer in *ast^ti272z^* mutants. We found that dendrites from forward DS tectal cells in the *ast*^*ti272z*^ tectum lose their characteristic laminar profile and are instead diffuse (arrowheads in Figure 4F; Movie S6). However, by overlaying line plots showing the distribution of forward tuned DS tectal dendrites and RGCs in the mutant, we show that the two profiles nevertheless still show considerable overlap (Figure 4H). To quantify the change in morphology of tectal dendrites, we measured four morphological features of all imaged tectal cells in WT and *ast*^*ti272z*^ tecta (Figure S7A). Total arbor length, distance of distal arbor from skin, and the anterior-posterior span of the distal arbor are not significantly different in mutant and WT larvae (Figures 4I–4K). However, dendrites of tectal cells have a significantly broader laminar profile in *ast*^*ti272z*^ mutants (Figure 4L; WT: 14.9 ± 1.2, *ast*^*ti272z*^: 28.2 ± 2.4, p < 0.001), and a higher proportion of them possess diffuse dendritic arbors (>16 μm extent in laminar axis) (Figure 4M; WT: 33%, *ast*^*ti272z*^: 84%). Thus, dendritic lamination is lost but tuning properties of tectal cells targeted by the *FoxP2.A:Gal4FF* are unaltered in the mutant. These data suggest a model of functional recovery in which structural plasticity in the mutant tectum establishes functional connections between DS-RGCs and their normal tectal targets. It is interesting to note that tectal cells tuned to posterior motion and some vertically tuned tectal neurons also have laminar dendrites and that this lamination appears to be lost in the mutant tectum. This suggests that structural plasticity perhaps ensures appropriate functional wiring of multiple tectal neuron subtypes when lamination cues are lost (Figure 4L).

### Robo2 Guides Laminar Growth of Tectal Cell Dendrites

**Figure 5:**
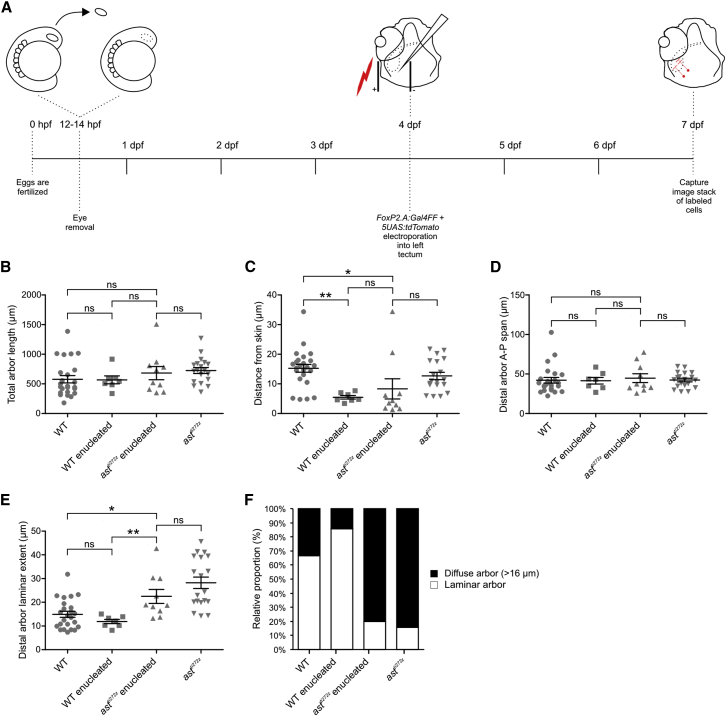
Robo2 Directs Laminar Growth of Tectal Cell Dendrites (A) Experimental procedure used to assess the role of RGC axons and/or Robo2 in regulating the morphology of tectal cell dendrites. Optic vesicles were removed at 12–14 hpf. Single cells were labeled by co-electroporating *FoxP2.A:Gal4FF* + *5UAS:tdTomato* DNA constructs into the deprived tectum at 4 dpf. Single tectal cells were imaged at 7 dpf. (B–E) Comparison of morphological parameters of tectal cells in four conditions: WT (n = 24 cells in 22 larvae), WT enucleated (n = 7 cells in 6 larvae), *ast*^*ti272z*^ enucleated (n = 10 cell in 9 larvae), and *ast*^*ti272z*^ (n = 19 cells in 16 larvae). For total arbor length (B), 682.6 ± 62.4 for WT, 567.4 ± 66.7 for WT enucleated, 681.7 ± 110.4 for *ast*^*ti272z*^ enucleated, and 724.6 ± 50.5 for *ast*^*ti272z*^; for distance from skin (C), 15.2 ± 1.3 for WT, 5.4 ± 0.5 for WT enucleated, 8.3 ± 3.4 for *ast*^*ti272z*^ enucleated, and 12.6 ± 1.2 for *ast*^*ti272z*^; for distal arbor anterior-posterior span (D), 42 ± 3.6 for WT, 41.4 ± 4.1 for WT enucleated, 44.6 ± 5.6 for *ast*^*ti272z*^ enucleated, and 42.3 ± 2.2 for *ast*^*ti272z*^; and for distal arbor laminar extent (E), 14.9 ± 1.2 for WT, 11.8 ± 0.9 for WT enucleated, 22.4 ± 2.9 for *ast*^*ti272z*^ enucleated, and 28.2 ± 2.4 for *ast*^*ti272z*^. All graphs show mean values ± SEM. ^∗∗^p < 0.01; ^∗^p < 0.05; ns, not significant, Kruskal-Wallis and Dunn’s multiple comparison tests. (F) Proportions of cells with laminar and diffuse (distal arbor laminar extent < 16 μm and > 16 μm, respectively) arbors within each experimental group. WT: 67% laminar, 33% diffuse arbor; WT enucleated: 86% laminar, 14% diffuse arbor; *ast*^*ti272z*^ enucleated: 20% laminar, 80% diffuse arbor; and *ast*^*ti272z*^: 16% laminar, 84% diffuse arbor.

Two mechanisms may potentially account for the switch from a laminar to a diffuse dendritic morphology of tectal cells in the mutant tectum. RGC axons may simply act as a scaffold for tectal dendrites—if DS-RGC axons are diffusely distributed in the mutant then so are the dendrites of tectal neurons. Alternatively, Robo2 may play a direct role in guiding laminar growth of tectal cell dendrites. In addition to the expression in RGCs, Robo2 is also expressed in tectal neurons but its role here is unknown (Campbell et al., 2007). To address this, we enucleated WT and mutant zebrafish prior to RGC innervation of the tectum in order to remove any positional information that might be provided to tectal dendrites by RGC axons. We then mosaically labeled tectal cells by co-electroporating *FoxP2.A:Gal4FF* and *5UAS:tdTomato* DNA constructs into tecta deprived of retinal input. Labeled cells were imaged at 7 dpf and morphologically reconstructed (Figure 5A). Total dendritic arbor length and anterior-posterior span were unaltered by enucleation in either WT or *ast*^*ti272z*^ larvae (Figures 5B and 5D). However, while tectal cells in WT enucleated animals retain their laminar morphology (Figure 5E, laminar extent of dendrites = 11.8 μm ± 0.9; Figure 5F, proportion of laminar arbors = 86%), lamination of tectal dendrites is lost in enucleated *ast*^*ti272z*^ larvae (Figures 5E, laminar extent = 22.4 μm ± 2.9; Figure 5F, proportion of laminar arbors = 20%). Thus, even in the absence of disorganized RGC axons, tectal dendrites lose dendritic stratification in the *ast*^*ti272z*^ tectum. These findings suggest that Robo2 can direct laminar growth of DS tectal cell dendrites.

### Rapid Development of Tectal Direction Selectivity Requires Robo2 in RGC Axons and Tectum

**Figure 6:**
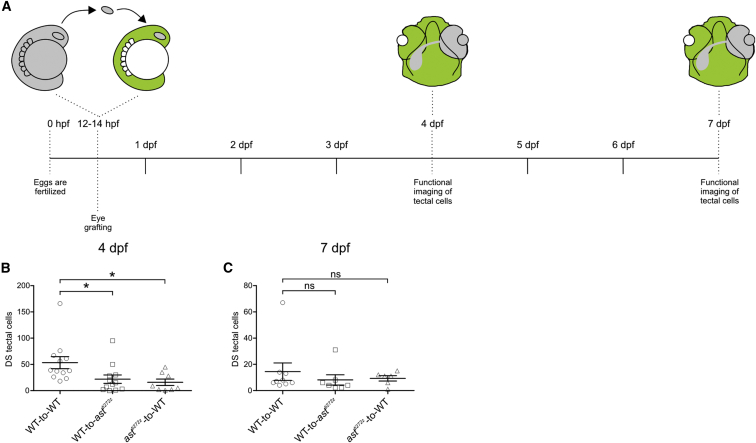
Robo2 Is Required in RGC Axons and Tectum for Rapid Development of DS Responses in Tectal Cells (A) Experimental procedure used to assess the cellular requirements for Robo2 in the functional development of DS tectal cells. Optic vesicles from donor embryos were transplanted into *Tg(elavl3:GCaMP5G)* hosts at 12-14 hpf and functional imaging of tectal cell responses was performed at 4 and 7 dpf. (B and C) Quantification of average number of DS tectal cells per group. Total number of DS cells at 4 dpf (B), 53.4 ± 11.5 for WT-to-WT (n = 12 larvae, total of 36 optical sections), 21.9 ± 7.9 for WT-to-*ast*^*ti272z*^ (n = 12 larvae, total of 36 optical sections), and 15.8 ± 6.1 for *ast*^*ti272z*^-to-WT (n = 8 larvae, total of 24 optical sections). Total number of DS cells at 7 dpf (C), 14.4 ± 6.6 for WT-to-WT (n = 9 larvae, total of 27 optical sections), 8.1 ± 3.8 for WT-to-*ast*^*ti272z*^ (n = 7 larvae, total of 21 optical sections), and 9.3 ± 2 for *ast*^*ti272z*^-to-WT (n = 6 larvae, total of 18 optical sections). All graphs show mean values ± SEM. ^∗^p < 0.05; ns, not significant, Kruskal-Wallis and Dunn’s multiple comparison tests. See also Figure S8.

Our data show that Robo2 can drive lamination of both tectal dendrites and RGC axons. Thus, by bringing axons and dendrites into precise spatial alignment Robo2 may speed the functional development of retinotectal connections. In this scenario, Robo2 expression would be required in both RGCs and tectal neurons. To test this, we transplanted mutant eyes into WT hosts and vice-versa prior to retinal differentiation and RGC innervation of the tectum. The WT and *ast*^*ti272z*^ hosts had pan neuronal expression of GCaMP5G (*Tg(elavl3:GCaMP5G*), allowing us to perform functional imaging of the tectal population receiving input from the transplanted eye at 4 dpf and then again at 7 dpf (Figure 6A). To confirm the success of each transplantation experiment, we fixed samples post-functional imaging at 7 dpf and we bulk-loaded the donor and host eyes with the lipophilic dyes, DiI and DiD, respectively, in order to label the entire retinotectal projection (Figures S8A–S8D). As an additional control, we transplanted either mutant or WT eyes expressing SyGCaMP3 and then performed functional imaging of RGC axons originating from the transplanted eye (Figure S8E). This showed that regardless of the host-donor combination, similar numbers of DS-RGC voxels were found in the host tectum, indicating that functional development of DS-RGCs and innervation of the tectum is unaffected by the transplantation procedure or the differing genotypes of host and donor (Figures S8F and S8G). Each transplantation experiment fell within one of the following three groups: WT-to-WT group, which serves as a control group, and two experimental groups WT-to-*ast*^*ti272z*^ and *ast*^*ti272z*^-to-WT transplants. Functional imaging of the population of tectal cells revealed that WT-to-*ast*^*ti272z*^ and *ast*^*ti272z*^-to-WT groups have, on average, significantly less DS tectal cells compared to the WT-to-WT group at 4 dpf (Figure 6B; WT-to-WT: 53.4 ± 11.5, WT-to-*ast*^*ti272z*^: 21.9 ± 7.9, and *ast*^*ti272z*^-to-WT: 15.8 ± 6.1, p < 0.05). These findings indicate that Robo2 expression is required in both RGCs and tectal neurons for the rapid (by 4 dpf) development of DS retinotectal connections. However, at 7 dpf, the number of DS tectal cells in the two experimental groups match those found in the control, WT-to-WT group (Figure 6C), suggesting that DS tectal responses eventually recover in tecta receiving input from transplanted eyes.

### Structural Plasticity of RGC Axons or Tectal Neurons Compensates for Loss of Robo2

**Figure 7:**
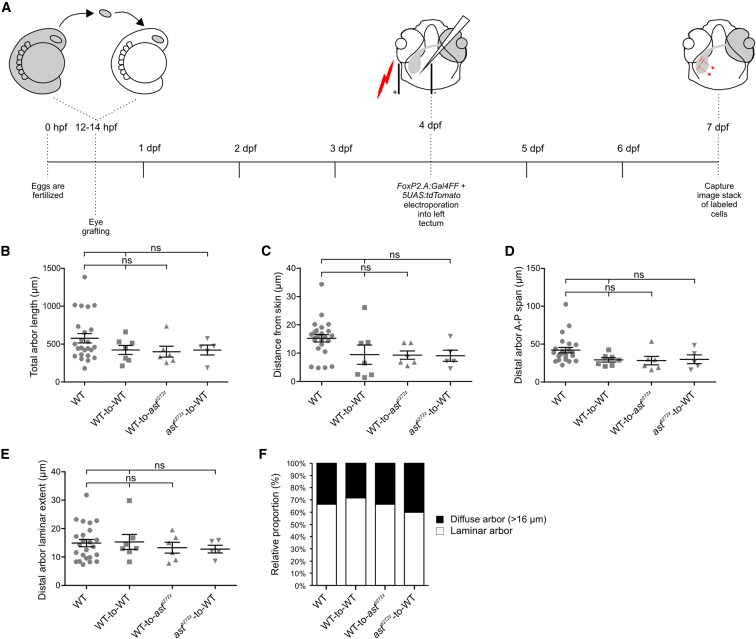
*robo2*^*+/+*^ RGC Axons Provide Positional Cues for *robo2*^*−/−*^ Tectal Cell Dendrites (A) Schematic showing the experimental procedure followed. Optic vesicles from donor embryos were transplanted into hosts at 12–14 hpf. Single cells were labeled by co-electroporating *FoxP2.A:Gal4FF* + *5UAS:tdTomato* DNA constructs into the tectum receiving retinal input from transplanted eyes at 4 dpf and imaged at 7 dpf. (B–E) Comparisons of the main morphological parameters of tectal cells in four conditions: WT (n = 24 in 22 larvae), WT-to-WT (n = 7 in 6 larvae), WT-to-*ast*^*ti272z*^ (n = 6 in 5 larvae), and *ast*^*ti272z*^-to-WT (n = 5 in 4 larvae) tectal cells. For total arbor length (B), 682.6 ± 62.4 for WT, 423.6 ± 60 for WT-to-WT, 399.5 ± 71.9 for WT-to-*ast*^*ti272z*^, and 421.8 ± 64.5 for *ast*^*ti272z*^-to-WT; for distance from skin (C), 15.2 ± 1.3 for WT, 9.4 ± 3.4 for WT-to-WT, 9.3 ± 1.4 for WT-to-*ast*^*ti272z*^, and 9 ± 1.9 for *ast*^*ti272z*^-to-WT; for distal arbor anterior-posterior span (D), 42 ± 3.6 for WT, 29.2 ± 2.9 for WT-to-WT, 28.2 ± 5.5 for WT-to-*ast*^*ti272z*^, and 29.9 ± 5.8 for *ast*^*ti272z*^-to-WT; and for distal arbor laminar extent (E), 14.9 ± 1.2 for WT, 15.3 ± 2.6 for WT-to-WT, 13.2 ± 1.8 for WT-to-*ast*^*ti272z*^, and 12.8 ± 1.3 for *ast*^*ti272z*^-to-WT. All graphs show mean values ± SEM ns, not significant, Kruskal-Wallis and Dunn’s multiple comparison tests. (F) Proportions of cells with laminar and diffuse (distal arbor laminar extent < 16 μm and > 16 μm, respectively) arbors within each experimental group. WT: 67% laminar, 33% diffuse arbor; WT-to-WT: 71% laminar, 29% diffuse arbor; WT-to-*ast*^*ti272z*^: 67% laminar, 33% diffuse arbor; and *ast*^*ti272z*^-to-WT: 60% laminar, 40% diffuse arbor.

**Figure 8:**
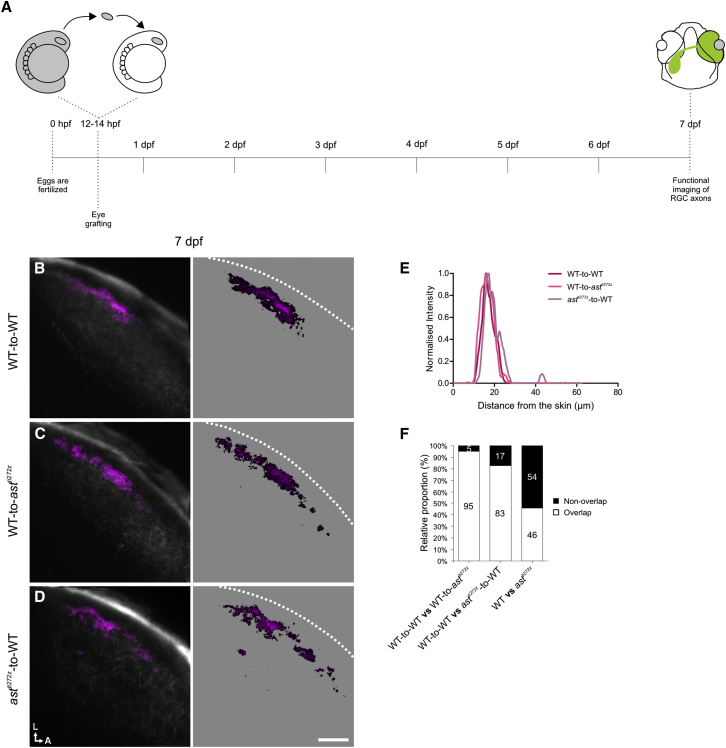
*robo2*^*+/+*^ Tectal Cell Dendrites Provide Positional Cues for *robo2*^*−/−*^ RGC Axons (A) Schematic showing the experimental procedure followed. Optic vesicles from *Tg(Isl2b:Gal4;UAS:SyGCaMP3)* donor embryos were transplanted into hosts at 12–14 hpf and functional imaging of donor RGC axons within the tectum was performed at 7 dpf. (B–D) Composite parametric maps across all fish imaged showing the spatial distribution of forward tuned DS voxels in the tectal neuropil of WT-to-WT (n = 8 larvae, total of 24 optical sections) (B), WT-to-*ast*^*ti272z*^ (n = 4 larvae, total of 12 optical sections) (C), and *ast*^*ti272z*^-to-WT (n = 4 larvae, total of 12 optical sections) (D). Within individual parametric maps, voxel brightness is proportional to the summed incidence of the functional subtype across all larvae imaged. The standard space template image derived from the mean fluorescence image of SyGCaMP3-expressing axons (grayscale) provides an anatomical reference. Dashed lines indicate the position of the skin overlaying the tectum. Scale bar represents 20 μm. A, anterior; L, lateral. (E) Line plots generated from the composite parametric maps in (B)–(D) illustrating the laminar organization of forward tuned DS voxels within each group. Line plots show normalized intensity of DS voxels as a function of distance from the skin. (F) Pairwise comparisons showing the extent of spatial overlap in the distribution of forward tuned DS voxels in the different experimental groups. Note the increased fraction of correctly positioned DS voxels in transplanted *ast*^*ti272z*^ axons (*ast*^*ti272z*^-to-WT group) compared with the *ast*^*ti272z*^ condition.

The recovery of tectal direction selectivity in the transplants is surprising because of the molecular mismatch between tectal cells and RGC axons—the Robo2-positive cell type has a lamination cue while the Robo2-negative neuron does not. How then do the two mismatched cell types eventually connect with one another? To address this question, we transplanted WT eyes expressing SyGCaMP3 in RGCs into *ast*^*ti272z*^ hosts (Figure 8A). By performing functional imaging and generating composite functional maps, we show that the WT transplanted DS-RGCs show their normal sublaminar distribution within SFGS, consistent with a role for Robo2 in laminar targeting of RGC axons (Figures 8B, 8C, and 8E). Furthermore, this suggests that functional recovery in the WT-to- *ast*^*ti272z*^ transplants is not due WT RGCs shifting their position within the mutant tectum in order to locate the misplaced tectal dendrites. Functional recovery must therefore result from structural plasticity of tectal neurons in the *ast*^*ti272z*^ tectum. To test this, we mosaically labeled tectal cells by co-electroporating *FoxP2.A:Gal4FF* and *5UAS:tdTomato* DNA constructs into the tectum receiving input from the WT transplanted eye. Labeled cells were imaged at 7 dpf and morphologically reconstructed (Figure 7A). Strikingly, when Robo2-negative tectal neurons receive input from WT RGCs, the proportion of cells that are laminar is identical to that seen in WT animals and in the WT-to-WT transplant controls (Figures 7E and 7F). Furthermore, dendrites of Robo2-negative tectal neurons that receive input from WT RGCs are morphologically indistinguishable from cells labeled in the WT tectum (Figures 7B–7D). These findings suggest that WT RGC axons can provide lamination cues for Robo2-negative tectal neurons, which themselves exhibit sufficient structural plasticity to target the correct lamina despite the loss of lamination cues provided by Robo2. We then performed the converse experiment by transplanting *ast*^*ti272z*^ eyes expressing SyGCaMP3 in RGCs into WT hosts (*ast*^*ti272z*^-to-WT group). We show that Robo2-positive tectal cells receiving input from Robo2-negative RGC axons have laminar dendrites (Figures 7E and 7F). This is consistent with a role for Robo2 in guiding growth of tectal dendrites and demonstrates that WT tectal neurons do not alter their dendritic morphology when receiving input from mutant RGC axons. Thus, in this scenario functional recovery of tectal direction selectivity must result from the shifting of DS-RGC axons to the correct lamina. To test this, we performed functional imaging of mutant DS-RGC axons in the WT tectum and generated composite functional maps at 7 dpf. Because of the technically challenging nature of the experiments, the numbers of animals imaged in this way were relatively low (n = 4). This combined with the fact that DS-RGCs tuned to upward and downward motion are rare means that composite maps generated for these subtypes appear patchy. It is therefore difficult to reliably quantify the distributions of these subtypes in the *ast*^*ti272z*^-to-WT group. For this reason, we focused on the largest subpopulation of DS-RGC tuned to forward motion. We find that the distribution of mutant DS-RGCs in WT hosts is significantly more laminar than that seen in *ast*^*ti272z*^ animals (compare Figure 8D with Figure 2D). Furthermore, the fraction of correctly positioned DS-RGC voxels is virtually normal when *ast*^*ti272z*^ RGC axons are within a WT tectum (Figures 8E and 8F). Thus, the presence of WT tectal dendrites restores the normal laminar functional architecture in Robo2-negative DS-RGCs. These findings also suggest that structural plasticity of Robo2-negative RGC axons enables them to locate their Robo2-positive tectal targets. Collectively, the above findings imply that (1) tectal cells and RGC axons can provide positional information to one another, and (2) structural plasticity in RGC axons or tectal dendrites can compensate for the lack of lamination cues provided by Robo2.

## Discussion

The aim of our study was to exploit the *astray* mutant zebrafish to investigate how loss of a layered neural organization impacts the functional development of neural circuits. We focused on the development of direction-selective circuits and found that while the laminar organization of DS retinal inputs to the tectum is lost in the *astray* tectum, the tuning properties of these inputs are normal. However, functionally intact DS input from the retina, by itself, is not sufficient to establish early development of DS circuits within the tectum. Thus, precise laminar targeting of retinal inputs to the tectum is required for rapid assembly of direction-selective circuits. A surprising finding from our study is that directional tuning in individual neurons and population encoding of directional motion by tectal neurons at later stages of development is indistinguishable from that seen in WT larvae. These findings echo those from the *reeler* mouse mutant that found that visual and somatosensory areas of the cortex were functionally intact despite the loss of a laminated architecture (Dräger, 1981, Guy et al., 2015, Lemmon and Pearlman, 1981, Simmons and Pearlman, 1983). Similarly mice with null mutations in *PlexinA4* or *Semaphorin6A* exhibit severe defects in lamina-specific neurite arborization of tyrosine hydroxylase-expressing dopaminergic amacrine cells (TH-ACs) and their synaptic partners—the intrinsically photosensitive RGCs (ipRGCs) (Matsuoka et al., 2011). However, the axons of TH-ACs still overlap with the dendrites of ipRGCs in ectopic locations, suggesting that specific wiring between these cell types persists in these mutants (Matsuoka et al., 2011). Because these studies focused on a single time point late in development, a role for laminae in speeding circuit assembly would not have been apparent. The ability to follow the functional development of circuits within a single animal is a particular strength of the zebrafish and is one that has allowed us to provide experimental evidence demonstrating a role for layers in the brain. It is important to note that although tuning of individual neurons and population encoding of directional motion are ultimately intact in the *astray* tectum, other aspects of circuit function, which cannot be measured using functional calcium imaging, may not. There is increasing evidence that sophisticated local processing may be carried out within the dendritic tree, with nonlinear interactions between closely spaced synaptic inputs shaping the output of the neuron (Branco and Häusser, 2010). The loss of a laminar dendritic profile in tectal dendrites in the *astray* tectum that we and others have observed (Xiao et al., 2011) is likely to significantly alter the spatial relationship between synaptic inputs and consequently alter how these inputs are integrated in tectal neurons. Indeed, the optic tectum of *astray* mutants may be a useful model for understanding how changes in dendritic geometry alter the integrative properties of neurons.

Our study also provides new information on the molecular mechanisms that guide laminar-specific targeting of neurites and in so doing provides insight into precisely why lamination speeds circuit assembly. We show that in addition to targeting of DS-RGC axons, Robo2 guides laminar targeting of DS-tectal cell dendrites. This finding supports a simple model for how Slit-Robo signaling brings pre- and postsynaptic partners into rapid and precise alignment in the nascent tectal neuropil: a gradient of the Robo2 ligand, Slit1a, across the laminar axis of the tectum could provide positional information to growing RGC axons and tectal dendrites. Appropriately, matched axons and dendrites may interpret this gradient similarly, perhaps by expressing similar levels of the Robo2 receptor, thereby bringing cognate pre- and postsynaptic partners into spatial proximity. In this model, lamination cues such as Robo2 could speed circuit assembly simply by increasing the likelihood of contact between matching pre- and postsynaptic neurons. Support for such a model comes from our finding that Robo2 is required in both RGCs and tectal neurons for the rapid assembly of DS circuits in the tectum. A role for Slit-Robo signaling in coordinating the spatial arrangement of both pre- and postsynaptic elements has also been demonstrated in the leg neuropil of *Drosophila*, suggesting that an evolutionarily conserved role for Slit-Robo signaling as a global neuropilar organizer (Brierley et al., 2009).

We have also provided insight into the plasticity mechanisms that allow functional circuits to establish themselves when lamination is perturbed. Our transplantation experiments, in which one synaptic partner expresses Robo2 while the other does not, demonstrates that structural plasticity of Robo2-negative RGC axons or tectal dendrites is sufficient to compensate for the loss of this lamination cue. Time-lapse imaging studies of tectal dendrite and RGC axon growth in the normal zebrafish tectum have shown that both are extremely dynamic processes (Kaethner and Stuermer, 1992, Kaethner and Stuermer, 1997, Meyer and Smith, 2006, Niell et al., 2004, Simpson et al., 2013). Thus, the structural plasticity that enables functional recovery in the *astray* tectum is likely to be part of a normal developmental program rather than a process triggered specifically by the loss of lamination cues. An obvious implication of these findings is that cell-type-specific wiring is ultimately determined by mechanisms unrelated to Robo2 and lamination. Studies of neurite patterning in the inner plexiform layer (IPL) of the retina have implicated homophilic and heterophilic cell-surface molecules on axons and dendrites in synaptic partner recognition. For example, members of the immunoglobulin-domain-containing superfamily (Sidekick 1 and 2, Dscam, DscamL, Cadherin 8 and 9) have been identified as being important elements of a cell-surface recognition code that ensures precise connectivity in the chick and mouse retina (Duan et al., 2014, Krishnaswamy et al., 2015, Yamagata and Sanes, 2008, Yamagata et al., 2002). The precise nature of the code in the tectum or superior colliculus is unknown. Although such code could match specific pre- and postsynaptic neurons, it could not provide an explicit mechanism for determining precisely where a given layer might form relative to other layers. A global neuropil organizer such as Robo2 would not only increase the speed and likelihood of contact between pre- and postsynaptic neurons expressing complementary cell-surface codes, it may also account for the stereotypic order of sublaminae found in the zebrafish tectum.

Our findings suggest that lamination is not ultimately required for the correct wiring of neural networks, but they do not rule out a role for lamination in generating wiring specificity in other species or areas of the brain. For example, in the mouse retina, perturbation of the type II Cadherins, *cdh8* and *cdh9*, disrupts laminar targeting of axons of bipolar cell types, BC2 and BC5, in the inner plexiform layer—a perturbation that results in defective functional connectivity with ON-OFF DS-RGCs (Duan et al., 2014). However, in this instance it is difficult to attribute the functional deficits to the lamination errors since Cadherins may also play a direct role in synapse formation. Furthermore, the role of layers may well be diverse. The principle driving force for a laminated architecture may even vary between subtypes of the same neuron type. For example, in zebrafish laminar targeting of all subtypes of RGC is precise from early developmental stages and functional imaging studies have shown that, in addition to directional tuning, selectivity for other visual features is established very soon after RGC axons first enter the tectum (Niell and Smith, 2005). For young larval zebrafish, which are crucially dependent on a functional visual system for survival, speed of circuit assembly may be the main evolutionary driver of a laminated architecture. However, studies of RGC subtypes innervating the mouse superior colliculus have revealed diverse and subtype-specific strategies for generating lamina-specific connections. For example, axons of the direction-selective J- and BD-RGC subtypes target definitive laminae within the superior colliculus early in postnatal development suggesting that, as in zebrafish, precise laminar targeting of RGC axons may facilitate rapid functional assembly of direction-selective circuits (Kim et al., 2010). In contrast, lamination of the non-direction-selective W3- and OFF-αRGC axons emerges gradually from an initially diffuse pattern (Huberman et al., 2008, Kim et al., 2010). Do these different developmental patterns reflect subtype-specific differences in the requirement for laminae themselves? Further studies are required to examine the diversity of roles played by layers in the brain, but our study demonstrates that while lamination appears to be dispensable for wiring specificity in direction-selective circuits, it is essential for the rapid functional assembly of these networks.

## Experimental Procedures

### Transgenic and Mutant Lines

Transgenic lines *Tg(Isl2b:Gal4)*zc60, *Tg(UAS:SyGCaMP3)kg1* and *Tg(elavl3:GCaMP5G)a4598* have been previously described (Ahrens et al., 2013, Ben Fredj et al., 2010, Nikolaou et al., 2012). The *ast*^*ti272z*^ mutant allele (Fricke et al., 2001) was kindly provided by Catherine Becker and Thomas Becker (University of Edinburgh).

### Functional Imaging, Voxel-Wise Analysis and Generation of Functional Maps

Visual stimulation, voxel-wise analysis, and generation of functional maps were performed as previously described (Hunter et al., 2013, Lowe et al., 2013, Nikolaou et al., 2012). (See also Supplemental Experimental Procedures.)

### DNA Electroporations

Tectal cells were mosaically labeled by co-electroporating the *FoxP2.A:Gal4FF* activator plasmid (FoxP2.A enhancer construct was a gift of Joshua Bonkowsky, University of Utah) together with GCaMP6F or tdTomato effector plasmids, as previously described (Hoegler and Horne, 2010). (See also Supplemental Experimental Procedures.)

### Tissue Micro-manipulations

Enucleations and eye transplantations were performed at 6–10 somite stage (12–14 hpf). (See also Supplemental Experimental Procedures.)

## Author Contributions

Conceptualization, N.N. and M.P.M.; Methodology, N.N. and M.P.M.; Investigation, NN; Formal Analysis, N.N. and M.P.M.; Writing – Original Draft, N.N. and M.P.M.; Writing – Review & Editing, N.N. and M.P.M.; Funding Acquisition, M.P.M.

## Supplemental Information

Document S1. Supplemental Experimental Procedures, Figures S1–S8, and Tables S1 and S2

Movie S1 (Related to Figure 1). Calcium Responses of SyGCaMP3-Expressing RGC Axons in the WT Optic Tectum Evoked by Drifting Bar Stimulus Tuning experiment from a *Tg(Isl2b:Gal4;UAS:SyGCaMP3)* larva (WT#6) summarized as a montage in Figure S1A. Raw fluorescence (left) and normalized ΔF/F (right) responses of SyGCaMP3-expressing RGC axons in the tectal neuropil evoked by drifting bar stimulus are shown. Movie encompasses an entire tuning experiment in which all 12 directions of bar motion are presented.

Movie S2 (Related to Figure 1). Calcium Responses of SyGCaMP3-Expressing RGC Axons in the *astray* Optic Tectum Evoked by Drifting Bar Stimulus Tuning experiment from a *Tg(Isl2b:Gal4;UAS:SyGCaMP3)* larva (*ast*^*ti272z*^#5) summarized as a montage in Figure S1D. Raw fluorescence (left) and normalized ΔF/F (right) responses of SyGCaMP3-expressing RGC axons in the tectal neuropil evoked by drifting bar stimulus are shown. Movie encompasses an entire tuning experiment in which all 12 directions of bar motion are presented.

Movie S3 (Related to Figure 3). Calcium Responses of GCaMP5G-Expressing Tectal Neurons in the WT Optic Tectum Evoked by Drifting Bar Stimulus Tuning experiment from a *Tg(elavl3:GCaMP5G)* larva (WT#2) summarized as a montage in Figure S4A. Raw fluorescence (left) and normalized ΔF/F (right) responses of GCaMP5G-expressing tectal cells in the tectum evoked by drifting bar stimulus are shown. Movie encompasses an entire tuning experiment in which all 12 directions of bar motion are presented.

Movie S4 (Related to Figure 3). Calcium Responses of GCaMP5G-Expressing Tectal Neurons in the *astray* Optic Tectum Evoked by Drifting Bar Stimulus Tuning experiments from a *Tg(elavl3:GCaMP5G)* larva (*ast*^*ti272z*^#2) that are presented as a montage in Figure S4D. Raw fluorescence (left) and normalized ΔF/F (right) responses of GCaMP5G-expressing tectal cells in the tectum evoked by drifting bar stimulus are shown. Movie encompasses an entire tuning experiment in which all 12 directions of bar motion are presented.

Movie S5 (Related to Figure 4). Calcium Responses in a Single GCaMP6F-Expressing FoxP2.A Tectal Cell Evoked by Drifting Bar Stimulus Time-lapse data showing the raw fluorescence (left) and normalized ΔF/F (right) responses of a single GCaMP6F-expressing tectal cell in the tectum evoked by drifting bar stimulus. Movie encompasses an entire tuning experiment in which all 12 directions of bar motion are presented.

Movie S6 (Related to Figure 4). Three-Dimensional Reconstructions of FoxP2.A-Labeled Tectal Cells Traced FoxP2.A tectal cells were reconstructed as shown. An example of a forward tuned DS tectal neuron in WT (top) and a*st*^*ti272z*^ (bottom) tectum is shown.

Document S2. Article plus Supplemental Information

## References

[bib1] Ahrens M.B., Orger M.B., Robson D.N., Li J.M., Keller P.J. (2013). Whole-brain functional imaging at cellular resolution using light-sheet microscopy. Nat. Methods.

[bib2] Baier H. (2013). Synaptic laminae in the visual system: molecular mechanisms forming layers of perception. Annu. Rev. Cell Dev. Biol..

[bib3] Ben Fredj N., Hammond S., Otsuna H., Chien C.B., Burrone J., Meyer M.P. (2010). Synaptic activity and activity-dependent competition regulates axon arbor maturation, growth arrest, and territory in the retinotectal projection. J. Neurosci..

[bib4] Bonkowsky J.L., Wang X., Fujimoto E., Lee J.E., Chien C.B., Dorsky R.I. (2008). Domain-specific regulation of foxP2 CNS expression by lef1. BMC Dev. Biol..

[bib5] Branco T., Häusser M. (2010). The single dendritic branch as a fundamental functional unit in the nervous system. Curr. Opin. Neurobiol..

[bib6] Brierley D.J., Blanc E., Reddy O.V., Vijayraghavan K., Williams D.W. (2009). Dendritic targeting in the leg neuropil of Drosophila: the role of midline signalling molecules in generating a myotopic map. PLoS Biol..

[bib7] Burrill J.D., Easter S.S. (1994). Development of the retinofugal projections in the embryonic and larval zebrafish (Brachydanio rerio). J. Comp. Neurol..

[bib8] Campbell D.S., Stringham S.A., Timm A., Xiao T., Law M.Y., Baier H., Nonet M.L., Chien C.B. (2007). Slit1a inhibits retinal ganglion cell arborization and synaptogenesis via Robo2-dependent and -independent pathways. Neuron.

[bib9] Chen T.W., Wardill T.J., Sun Y., Pulver S.R., Renninger S.L., Baohan A., Schreiter E.R., Kerr R.A., Orger M.B., Jayaraman V. (2013). Ultrasensitive fluorescent proteins for imaging neuronal activity. Nature.

[bib10] Chklovskii D.B., Koulakov A.A. (2004). Maps in the brain: what can we learn from them?. Annu. Rev. Neurosci..

[bib11] Dhande O.S., Huberman A.D. (2014). Retinal ganglion cell maps in the brain: implications for visual processing. Curr. Opin. Neurobiol..

[bib12] Dräger U.C. (1981). Observations on the organization of the visual cortex in the reeler mouse. J. Comp. Neurol..

[bib13] Duan X., Krishnaswamy A., De la Huerta I., Sanes J.R. (2014). Type II cadherins guide assembly of a direction-selective retinal circuit. Cell.

[bib14] Fleisch V.C., Neuhauss S.C. (2006). Visual behavior in zebrafish. Zebrafish.

[bib15] Fricke C., Lee J.S., Geiger-Rudolph S., Bonhoeffer F., Chien C.B. (2001). astray, a zebrafish roundabout homolog required for retinal axon guidance. Science.

[bib16] Gabriel J.P., Trivedi C.A., Maurer C.M., Ryu S., Bollmann J.H. (2012). Layer-specific targeting of direction-selective neurons in the zebrafish optic tectum. Neuron.

[bib17] Grama A., Engert F. (2012). Direction selectivity in the larval zebrafish tectum is mediated by asymmetric inhibition. Front. Neural Circuits.

[bib18] Guy J., Wagener R.J., Mock M., Staiger J.F. (2015). Persistence of functional sensory maps in the absence of cortical layers in the somsatosensory cortex of reeler mice. Cereb. Cortex.

[bib19] Hoegler K.J., Horne J.H. (2010). Targeting the zebrafish optic tectum using in vivo electroporation. Cold Spring Harb. Protoc..

[bib20] Huberman A.D., Manu M., Koch S.M., Susman M.W., Lutz A.B., Ullian E.M., Baccus S.A., Barres B.A. (2008). Architecture and activity-mediated refinement of axonal projections from a mosaic of genetically identified retinal ganglion cells. Neuron.

[bib21] Hunter P.R., Lowe A.S., Thompson I.D., Meyer M.P. (2013). Emergent properties of the optic tectum revealed by population analysis of direction and orientation selectivity. J. Neurosci..

[bib22] Kaethner R.J., Stuermer C.A. (1992). Dynamics of terminal arbor formation and target approach of retinotectal axons in living zebrafish embryos: a time-lapse study of single axons. J. Neurosci..

[bib23] Kaethner R.J., Stuermer C.A. (1997). Dynamics of process formation during differentiation of tectal neurons in embryonic zebrafish. J. Neurobiol..

[bib24] Kim I.J., Zhang Y., Meister M., Sanes J.R. (2010). Laminar restriction of retinal ganglion cell dendrites and axons: subtype-specific developmental patterns revealed with transgenic markers. J. Neurosci..

[bib25] Krishnaswamy A., Yamagata M., Duan X., Hong Y.K., Sanes J.R. (2015). Sidekick 2 directs formation of a retinal circuit that detects differential motion. Nature.

[bib26] Lemmon V., Pearlman A.L. (1981). Does laminar position determine the receptive field properties of cortical neurons? A study of corticotectal cells in area 17 of the normal mouse and the reeler mutant. J. Neurosci..

[bib27] Lowe A.S., Nikolaou N., Hunter P.R., Thompson I.D., Meyer M.P. (2013). A systems-based dissection of retinal inputs to the zebrafish tectum reveals different rules for different functional classes during development. J. Neurosci..

[bib28] Matsuoka R.L., Nguyen-Ba-Charvet K.T., Parray A., Badea T.C., Chédotal A., Kolodkin A.L. (2011). Transmembrane semaphorin signalling controls laminar stratification in the mammalian retina. Nature.

[bib29] Meyer M.P., Smith S.J. (2006). Evidence from in vivo imaging that synaptogenesis guides the growth and branching of axonal arbors by two distinct mechanisms. J. Neurosci..

[bib30] Niell C.M., Smith S.J. (2005). Functional imaging reveals rapid development of visual response properties in the zebrafish tectum. Neuron.

[bib31] Niell C.M., Meyer M.P., Smith S.J. (2004). In vivo imaging of synapse formation on a growing dendritic arbor. Nat. Neurosci..

[bib32] Nikolaou N., Lowe A.S., Walker A.S., Abbas F., Hunter P.R., Thompson I.D., Meyer M.P. (2012). Parametric functional maps of visual inputs to the tectum. Neuron.

[bib33] Pérez-Escudero A., de Polavieja G.G. (2007). Optimally wired subnetwork determines neuroanatomy of Caenorhabditis elegans. Proc. Natl. Acad. Sci. USA.

[bib34] Rivera-Alba M., Vitaladevuni S.N., Mishchenko Y., Lu Z., Takemura S.Y., Scheffer L., Meinertzhagen I.A., Chklovskii D.B., de Polavieja G.G. (2011). Wiring economy and volume exclusion determine neuronal placement in the Drosophila brain. Curr. Biol..

[bib35] Robles E., Filosa A., Baier H. (2013). Precise lamination of retinal axons generates multiple parallel input pathways in the tectum. J. Neurosci..

[bib36] Sanes J.R., Yamagata M. (2009). Many paths to synaptic specificity. Annu. Rev. Cell Dev. Biol..

[bib37] Sanes J.R., Zipursky S.L. (2010). Design principles of insect and vertebrate visual systems. Neuron.

[bib38] Simmons P.A., Pearlman A.L. (1983). Receptive-field properties of transcallosal visual cortical neurons in the normal and reeler mouse. J. Neurophysiol..

[bib39] Simpson H.D., Kita E.M., Scott E.K., Goodhill G.J. (2013). A quantitative analysis of branching, growth cone turning, and directed growth in zebrafish retinotectal axon guidance. J. Comp. Neurol..

[bib40] Xiao T., Staub W., Robles E., Gosse N.J., Cole G.J., Baier H. (2011). Assembly of lamina-specific neuronal connections by slit bound to type IV collagen. Cell.

[bib41] Yamagata M., Sanes J.R. (2008). Dscam and Sidekick proteins direct lamina-specific synaptic connections in vertebrate retina. Nature.

[bib42] Yamagata M., Weiner J.A., Sanes J.R. (2002). Sidekicks: synaptic adhesion molecules that promote lamina-specific connectivity in the retina. Cell.

