# Supplementary figures and images for: Lamination Speeds the Functional Development of Visual Circuits
Source: Neuron. 2015 Dec 2;88(5):999–1013. doi: 10.1016/j.neuron.2015.10.020 (PMC4674658; doi:10.1016/j.neuron.2015.10.020)

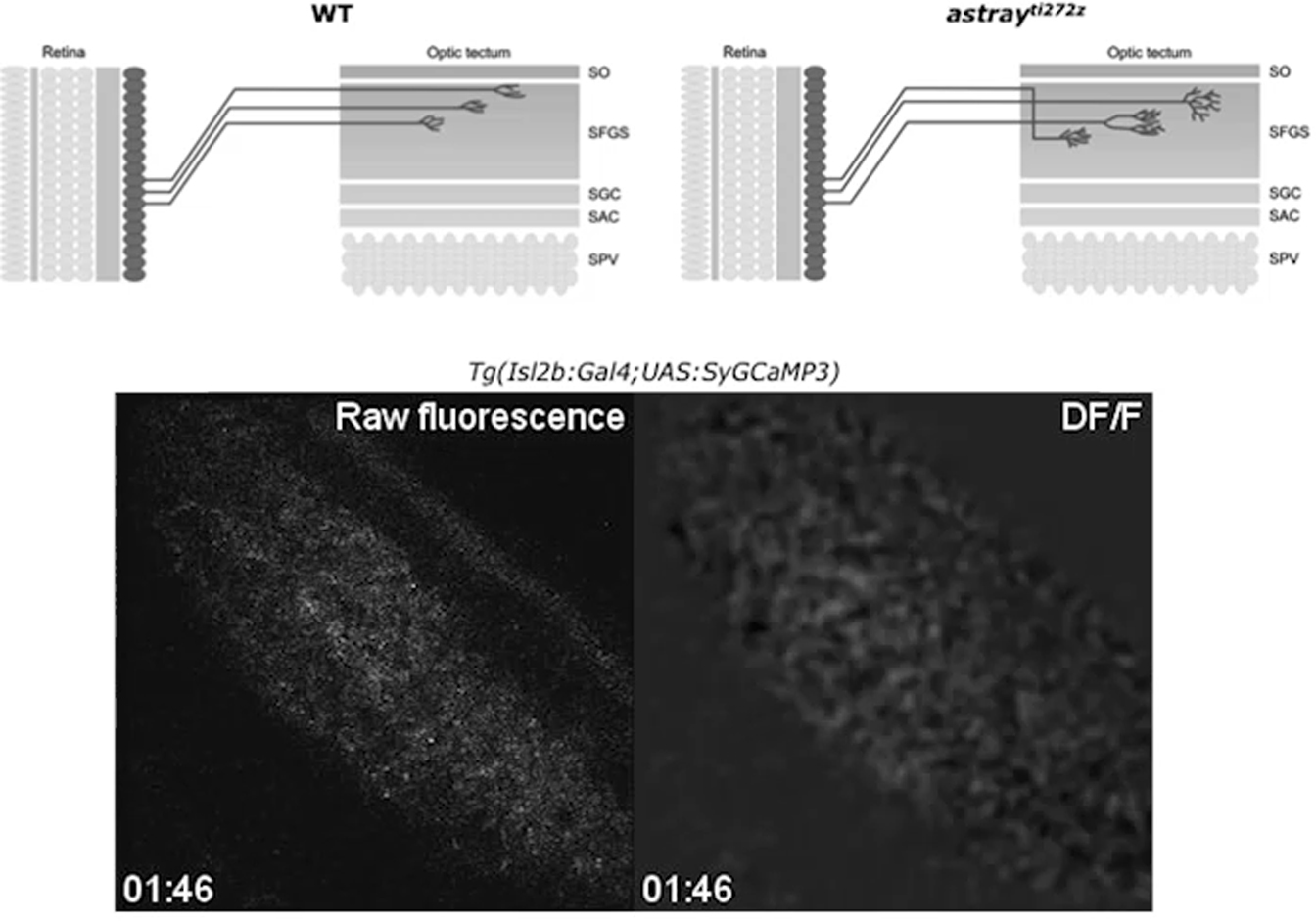

Supplement: Supplementary file 1 [file mmc9.jpg]

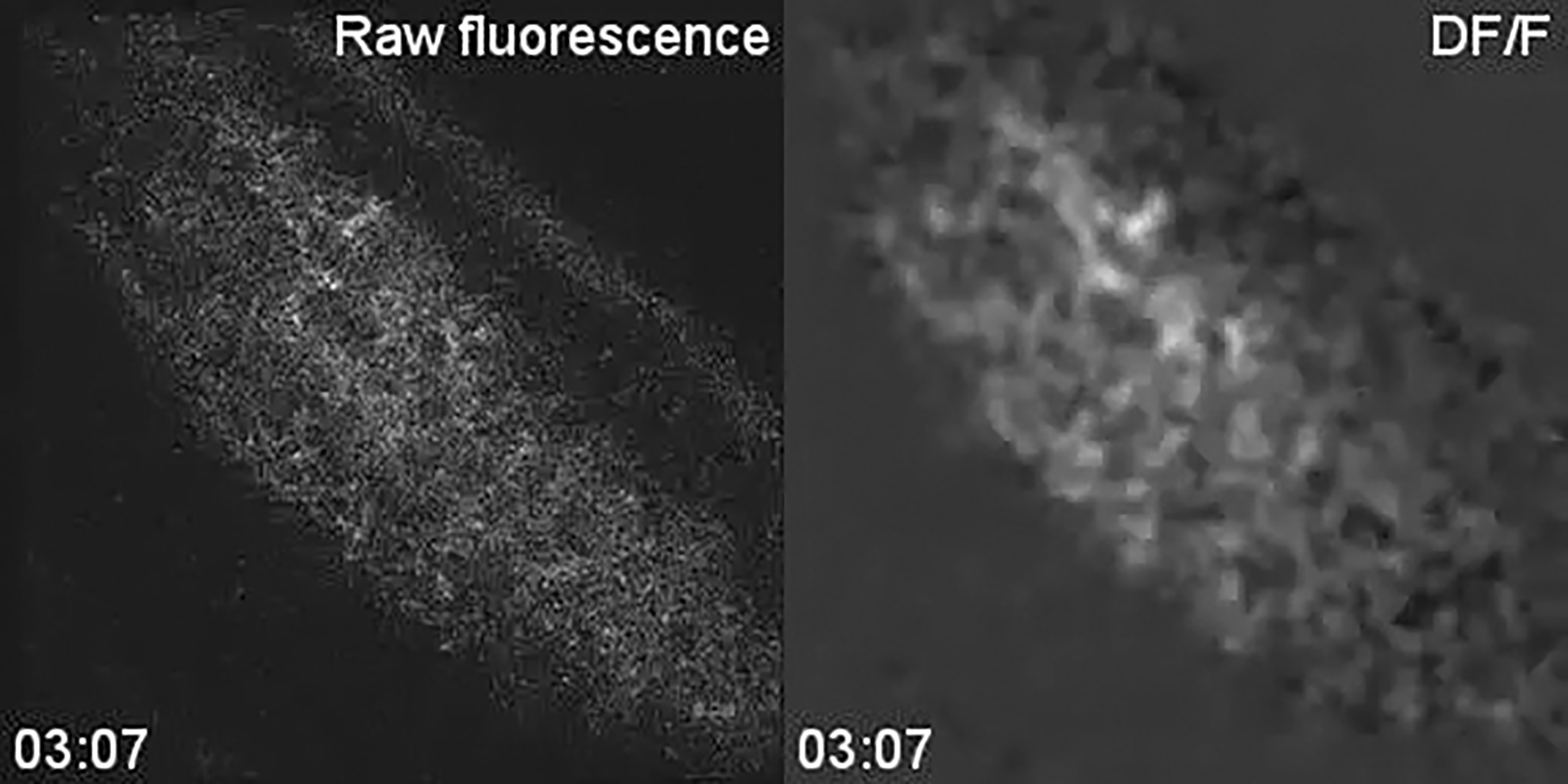

Supplement: Movie S1 (Related to Figure 1). Calcium Responses of SyGCaMP3-Expressing RGC Axons in the WT Optic Tectum Evoked by Drifting Bar Stimulus — Tuning experiment from a Tg(Isl2b:Gal4;UAS:SyGCaMP3) larva (WT#6) summarized as a montage in Figure S1A. Raw fluorescence (left) and normalized ΔF/F (right) responses of SyGCaMP3-expressing RGC axons in the tectal neuropil evoked by drifting bar stimulus are shown. Movie encompasses an entire tuning experiment in which all 12 directions of bar motion are presented. [file mmc2.jpg]

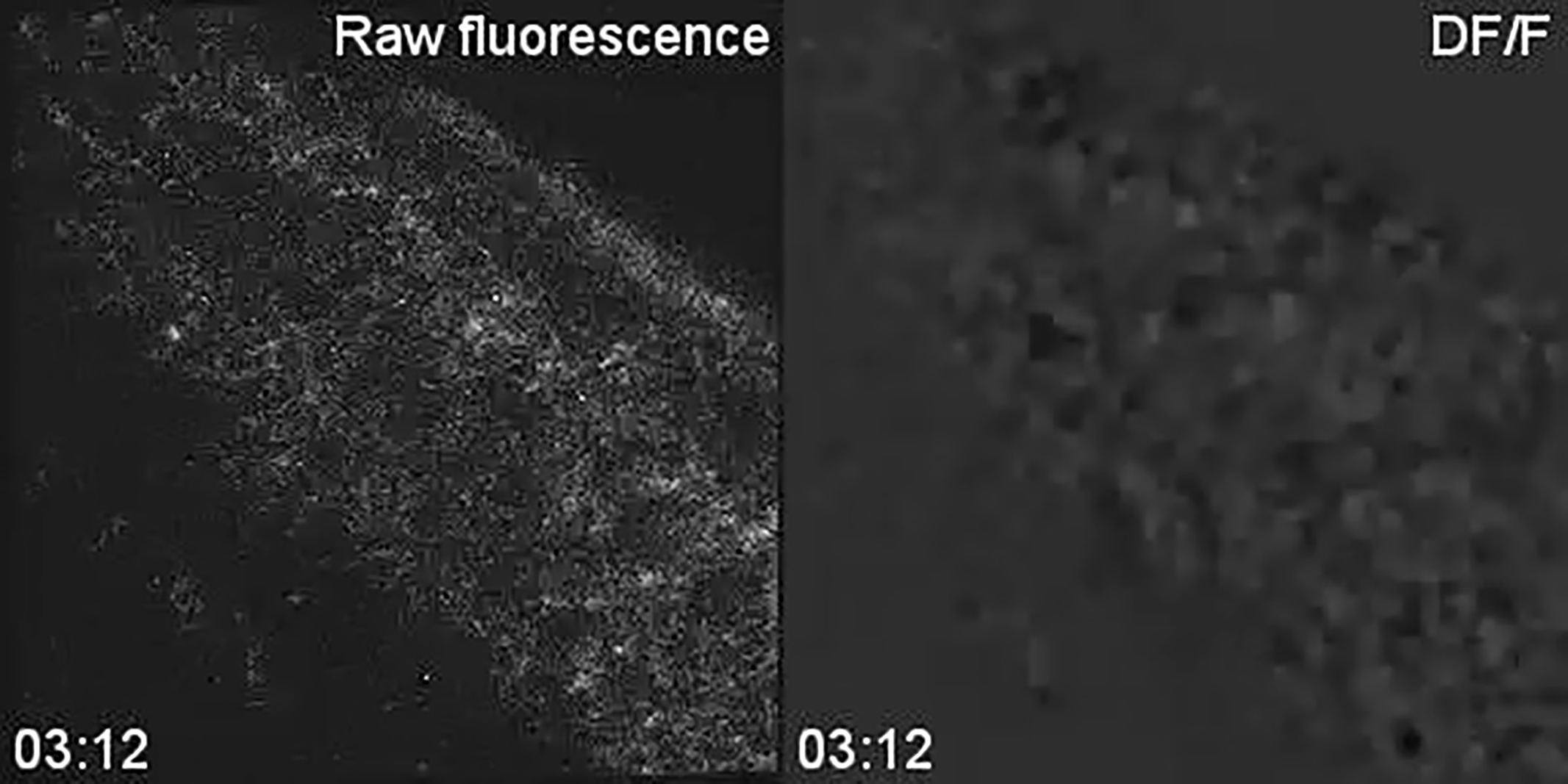

Supplement: Movie S2 (Related to Figure 1). Calcium Responses of SyGCaMP3-Expressing RGC Axons in the astray Optic Tectum Evoked by Drifting Bar Stimulus — Tuning experiment from a Tg(Isl2b:Gal4;UAS:SyGCaMP3) larva (astti272z#5) summarized as a montage in Figure S1D. Raw fluorescence (left) and normalized ΔF/F (right) responses of SyGCaMP3-expressing RGC axons in the tectal neuropil evoked by drifting bar stimulus are shown. Movie encompasses an entire tuning experiment in which all 12 directions of bar motion are presented. [file mmc3.jpg]

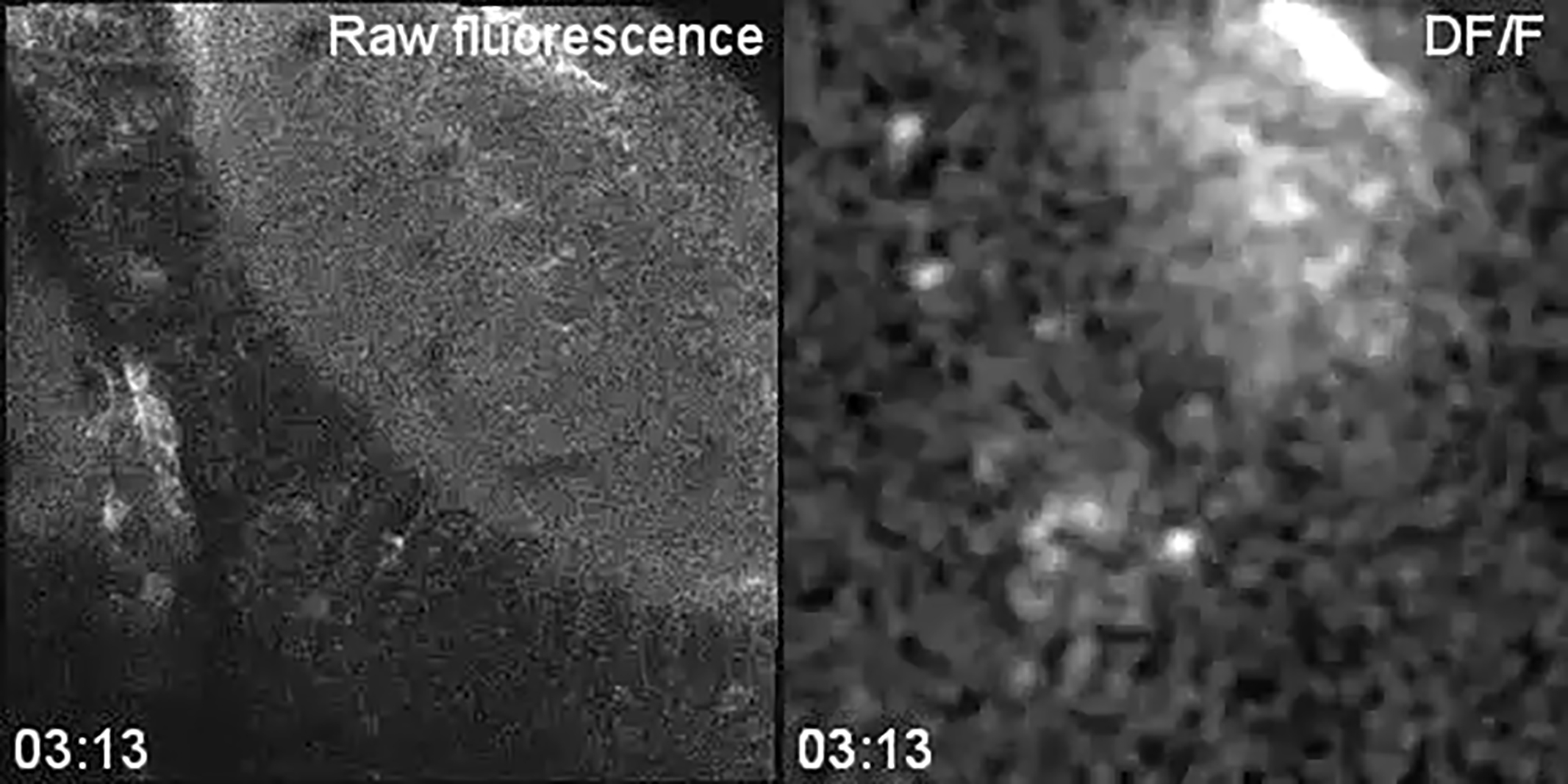

Supplement: Movie S3 (Related to Figure 3). Calcium Responses of GCaMP5G-Expressing Tectal Neurons in the WT Optic Tectum Evoked by Drifting Bar Stimulus — Tuning experiment from a Tg(elavl3:GCaMP5G) larva (WT#2) summarized as a montage in Figure S4A. Raw fluorescence (left) and normalized ΔF/F (right) responses of GCaMP5G-expressing tectal cells in the tectum evoked by drifting bar stimulus are shown. Movie encompasses an entire tuning experiment in which all 12 directions of bar motion are presented. [file mmc4.jpg]

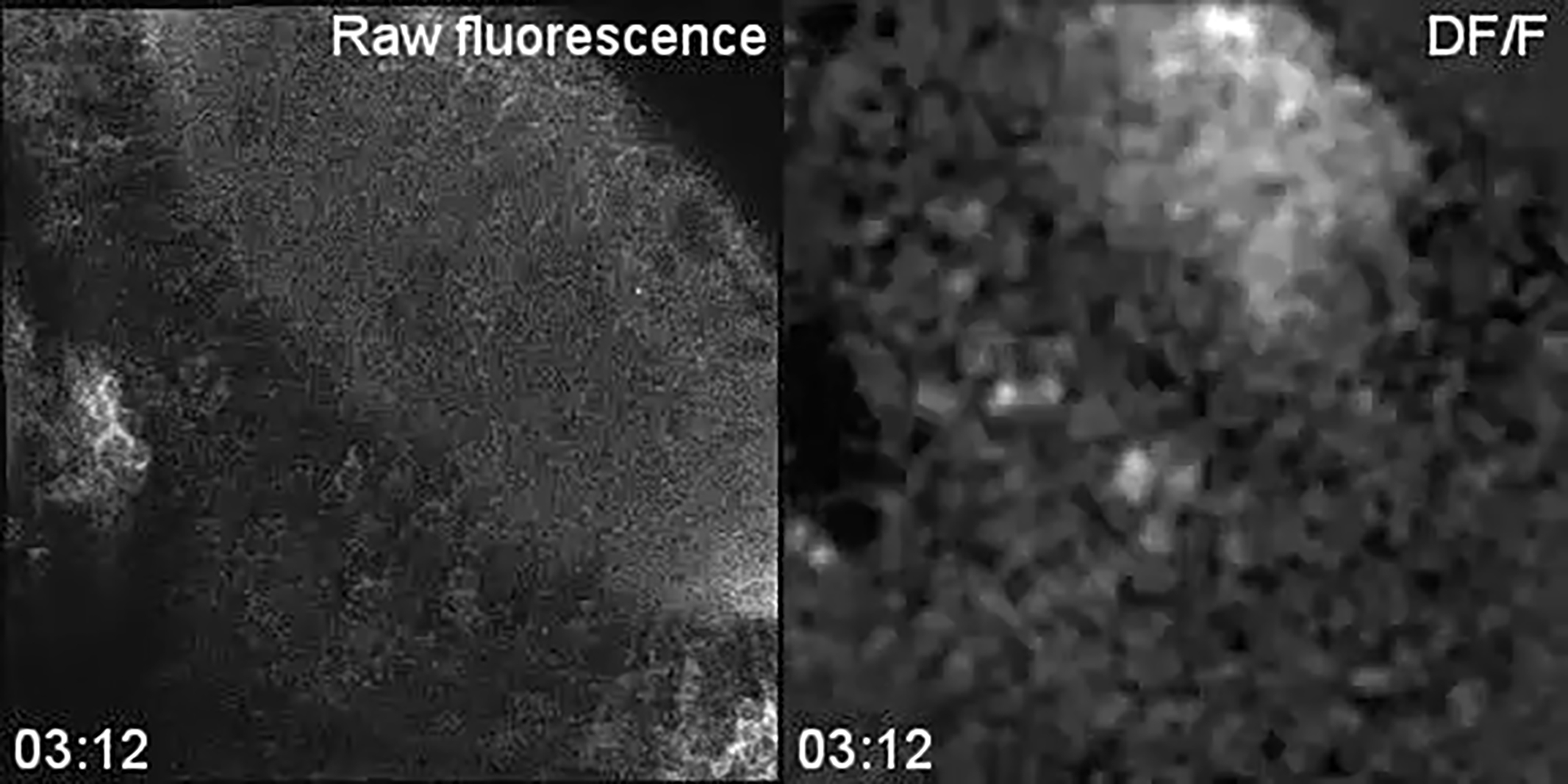

Supplement: Movie S4 (Related to Figure 3). Calcium Responses of GCaMP5G-Expressing Tectal Neurons in the astray Optic Tectum Evoked by Drifting Bar Stimulus — Tuning experiments from a Tg(elavl3:GCaMP5G) larva (astti272z#2) that are presented as a montage in Figure S4D. Raw fluorescence (left) and normalized ΔF/F (right) responses of GCaMP5G-expressing tectal cells in the tectum evoked by drifting bar stimulus are shown. Movie encompasses an entire tuning experiment in which all 12 directions of bar motion are presented. [file mmc5.jpg]

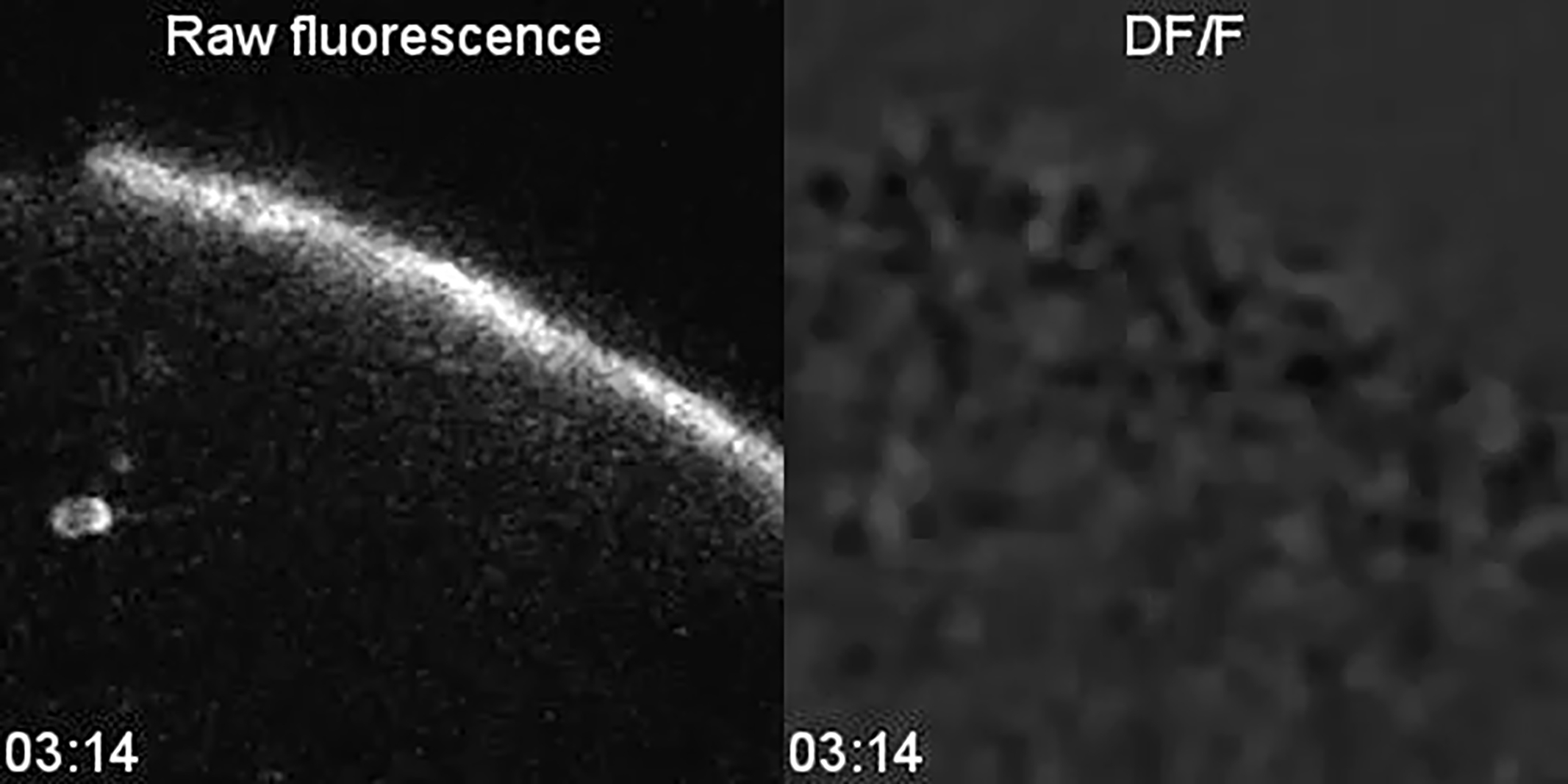

Supplement: Movie S5 (Related to Figure 4). Calcium Responses in a Single GCaMP6F-Expressing FoxP2.A Tectal Cell Evoked by Drifting Bar Stimulus — Time-lapse data showing the raw fluorescence (left) and normalized ΔF/F (right) responses of a single GCaMP6F-expressing tectal cell in the tectum evoked by drifting bar stimulus. Movie encompasses an entire tuning experiment in which all 12 directions of bar motion are presented. [file mmc6.jpg]

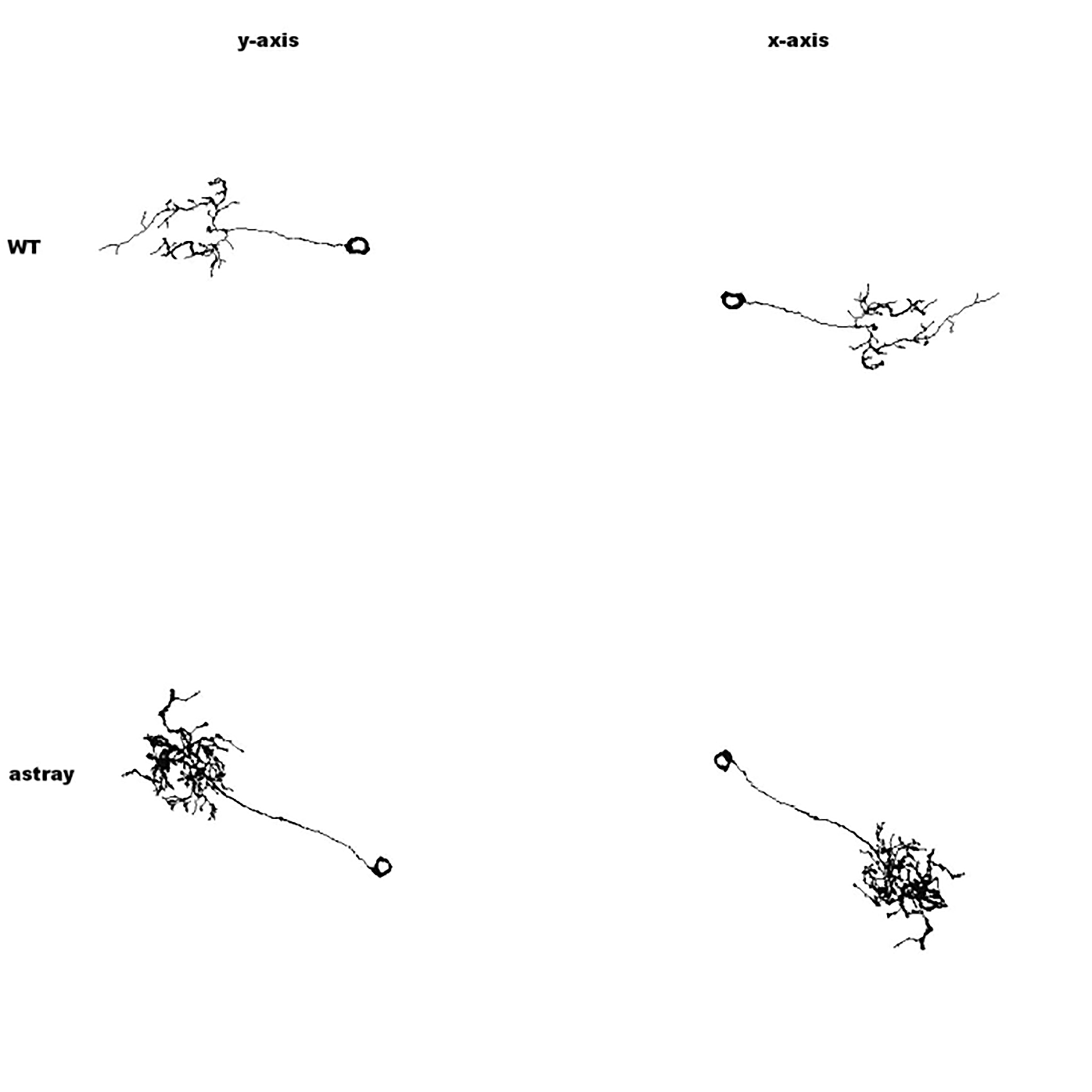

Supplement: Movie S6 (Related to Figure 4). Three-Dimensional Reconstructions of FoxP2.A-Labeled Tectal Cells — Traced FoxP2.A tectal cells were reconstructed as shown. An example of a forward tuned DS tectal neuron in WT (top) and astti272z (bottom) tectum is shown. [file mmc7.jpg]
