# Supplementary material for: Lamination Speeds the Functional Development of Visual Circuits
Source: Neuron. 2015 Dec 2;88(5):999–1013. doi: 10.1016/j.neuron.2015.10.020 (PMC4674658; doi:10.1016/j.neuron.2015.10.020)
Supplement: Document S1. Supplemental Experimental Procedures, Figures S1–S8, and Tables S1 and S2 [file mmc1.pdf]

Neuron

Supplemental Information

Lamination Speeds the Functional Development of Visual Circuits

Nikolas Nikolaou and Martin P. Meyer

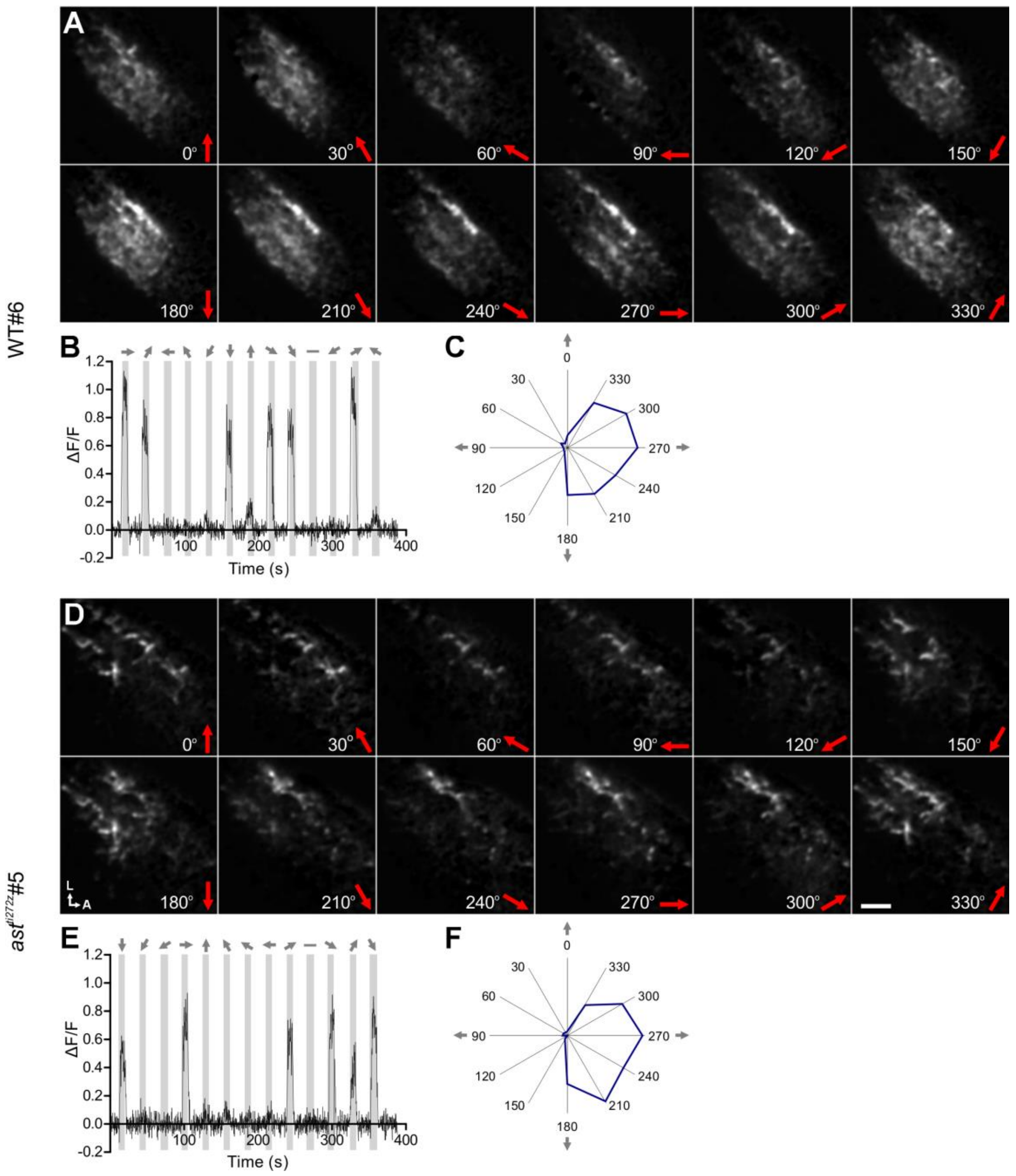

Figure S1. Responses of SyGCaMP3-expressing RGC axons terminating in the optic tectum (related to Figure 1). Examples of tuning experiments from WT (A-C) and *ast*^{ti272z} (D-F) *Tg(Isl2b:Gal4;UAS:SyGCaMP3)* larvae at 7 dpf are shown. (A, D) Montages showing integral responses (grayscale) of all RGC axons in the tectum. Direction of motion is shown on the bottom right in each panel. Scale bar represents 20 µm. A, anterior; L, lateral. (B, E) Representative response of single voxels during a tuning experiment. Stimulus epochs are shown in gray and direction of motion is indicated by arrows on the top. (C, F) Integral responses from (B) and (E) are shown in polar plot form.

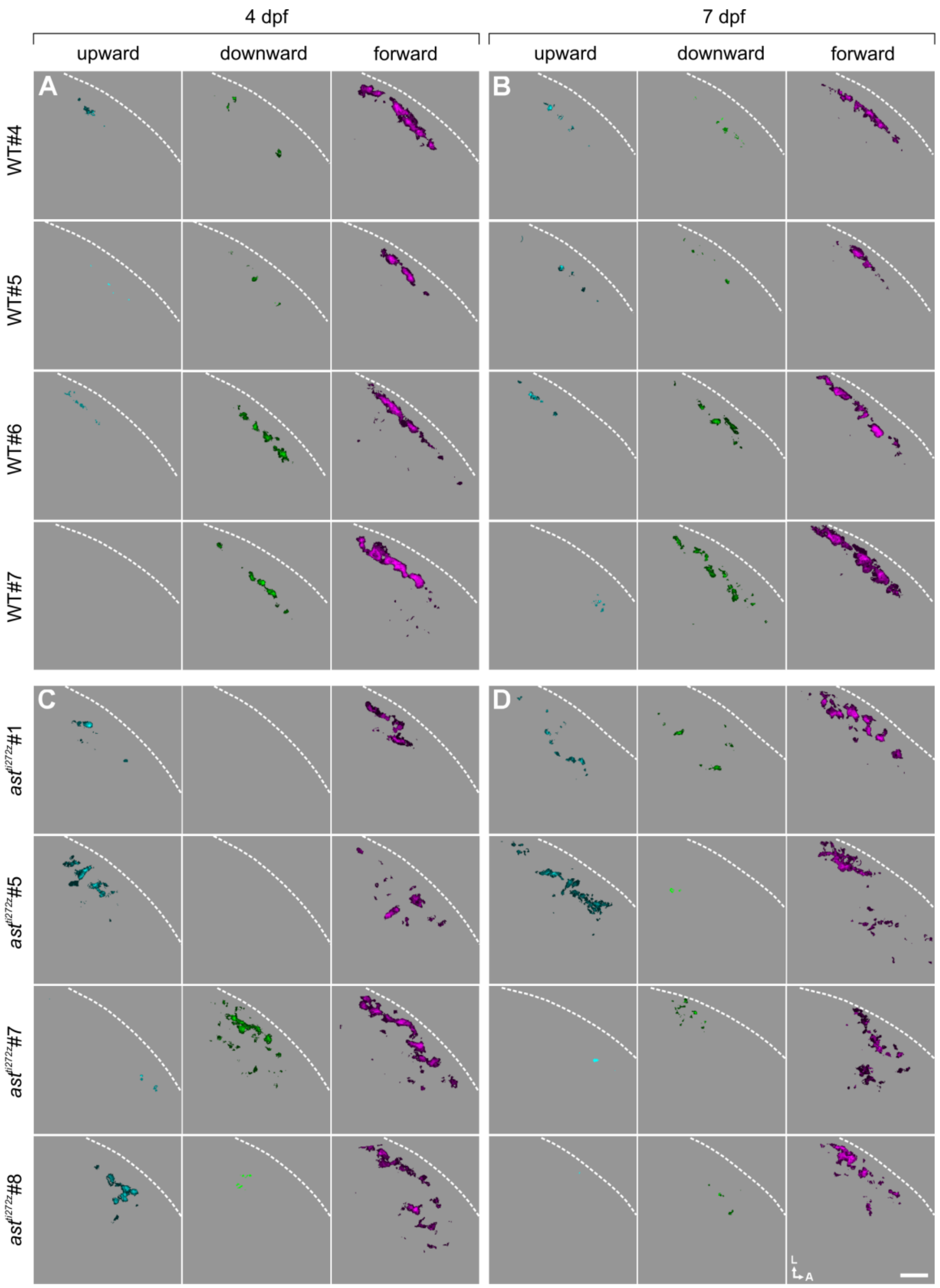

Figure S2. DS-RGC axons are diffusely distributed in the tectal neuropil of individual *astray* mutants (related to Figure 2). (A-D) Functional maps generated from single *Tg(Isl2b:Gal4;UAS:SyGCaMP3)* larvae showing the spatial distribution of DS-RGC subtypes in the tectal neuropil of WT larvae at 4 dpf (A) and 7 dpf (B), and *ast*^{ti272z} larvae at 4 dpf (C) and 7 dpf (D). Four examples of WT and *ast*^{ti272z} larvae are shown, respectively. Voxel brightness is proportional to the summed incidence of each functional subtype across all experiments

performed in each individual fish. Dashed lines indicate the position of the skin overlaying the tectum. Scale bar represents 20 µm. A, anterior; L, lateral.

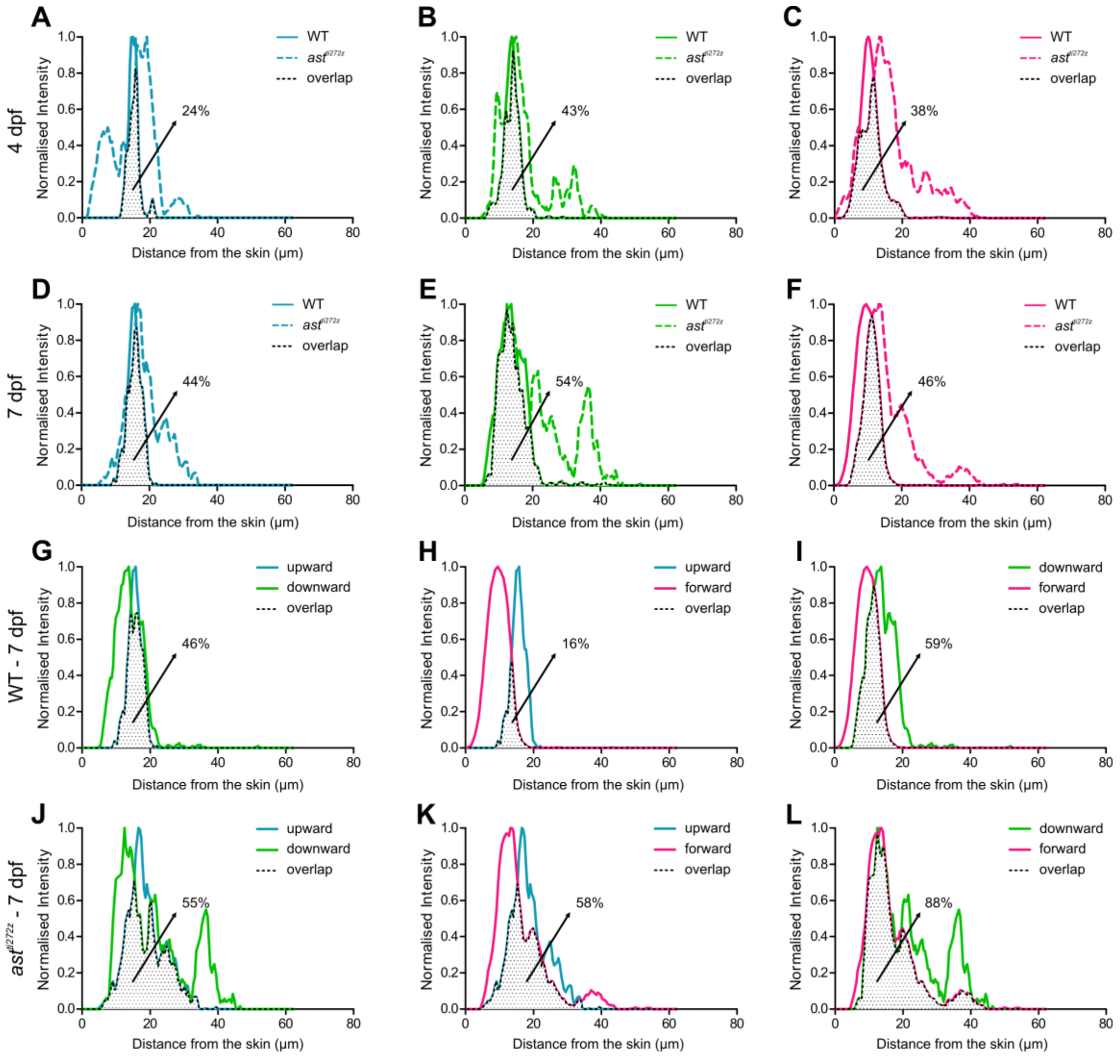

Figure S3. The lamination and segregation of DS-RGC axons is perturbed in *astray* mutants (related to Figure 2). Line plots generated from the composite parametric maps in Figures 2A-2D comparing the lamination (A-F) and segregation (G-L) of DS-RGC subtypes in WT (n = 8, total of 24 optical sections) and *ast*^{ti272z} (n = 9, total of 27 optical sections) tecta at 4 and 7 dpf. All plots show normalized intensities as a function of distance from the skin. (A-F) Pairwise comparison showing the degree of overlap for upward (A and D), downward (B and E) and forward (C and F) DS-RGC subtypes at 4 dpf (A-C) and 7 dpf (D-F). Dotted area represents the area of intersection between the WT and *ast*^{ti272z} domains. Values shown represent the fraction of all DS voxels that are located in the correct location in *ast*^{ti272z} tecta. (G-L) Pairwise comparisons showing the degree of spatial overlap between upward and downward (G and J), upward and forward (H and K) and downward and forward (I and L) DS-RGC subtypes within WT (G-I) and *ast*^{ti272z} (J-L) tecta at 7 dpf. Doted area represents the area of intersection between the two subtypes. Values shown represent the fraction of downward (for G and J) or forward (for H, I, K and L) DS voxels that spatially overlap with upward or downward DS voxels.

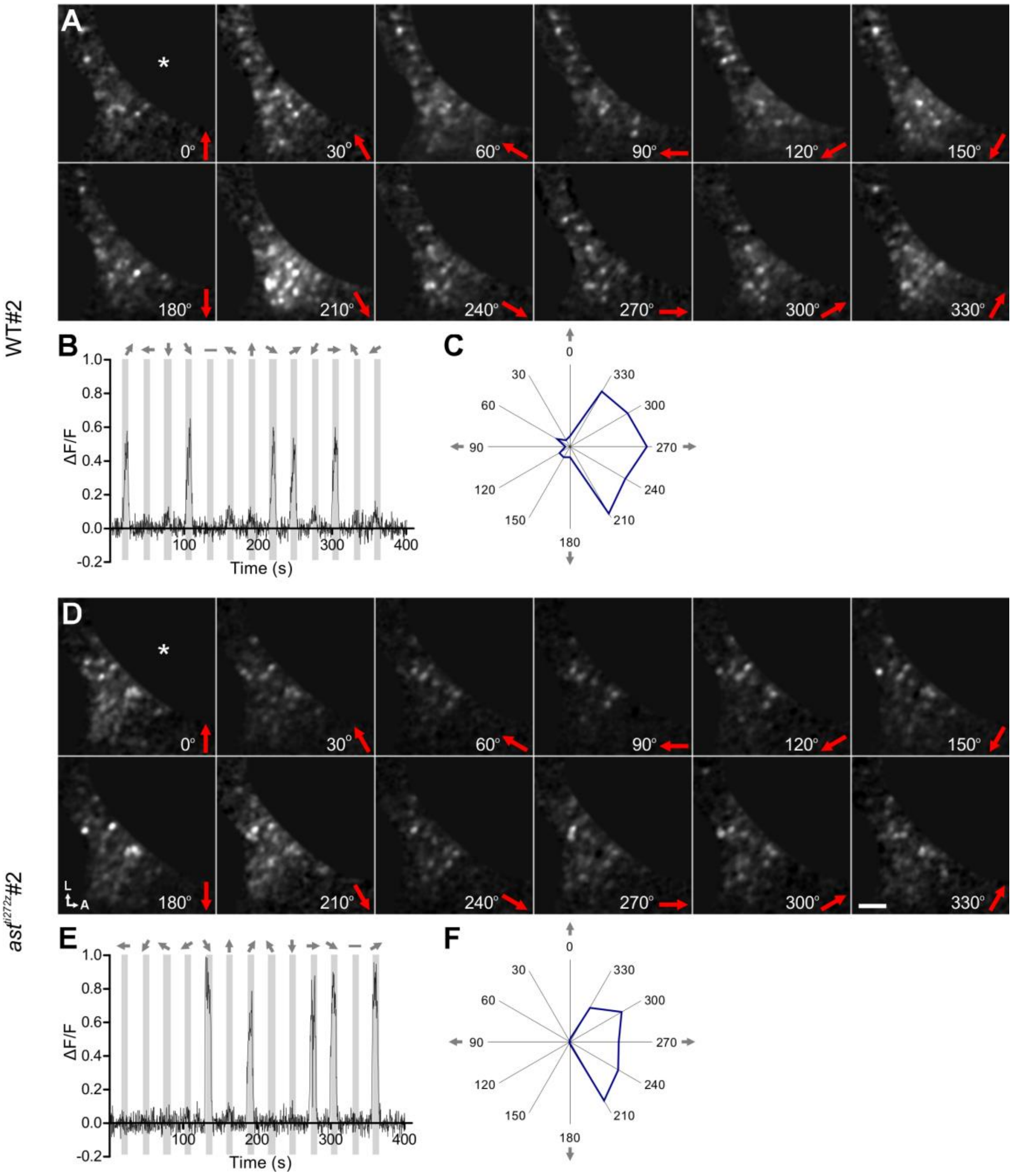

Figure S4. Responses of GCaMP5G-expressing tectal neurons in the optic tectum (related to Figure 3). Examples of tuning experiments from individual WT (A-C) and ast^{ti272z} (D-F) *Tg(elavl3:GCaMP5G)* larvae at 7 dpf are shown. Asterisks indicate the neuropil area of the tectum which has been removed for analysis purposes. (A, D) Montages showing integral responses (grayscale) of all voxels (tectal cell body area) in the tectum. Direction of motion is shown on the bottom right in each panel. Scale bar represents 30 µm. A, anterior; L, lateral. (B, E) Representative response of single voxels during a tuning experiment. Stimulus epochs are shown in gray and direction of motion is indicated by arrows on the top. (C, F) Integral responses from (B) and (E) are shown in polar plot form.

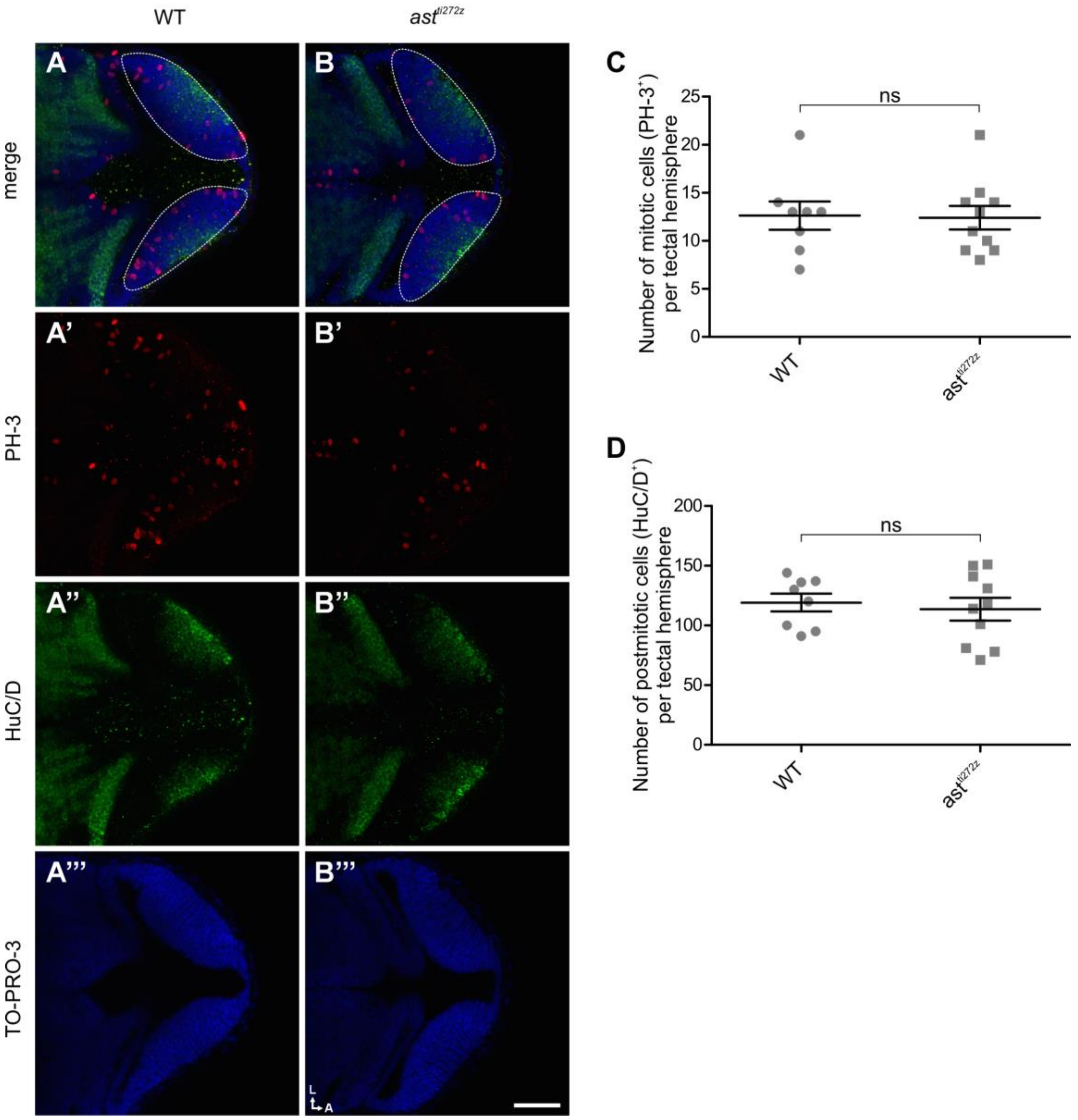

Figure S5. Tectal proliferation and differentiation are unaffected in *astray* mutants (related to Figure 3). Measurement of proliferation and differentiation in WT (n = 4) and ast^{ti272z} (n = 5) larvae at 48 hpf. (A-B) Single confocal sections showing dorsal views of WT (A) and ast^{ti272z} (B) embryos immunostained for PH-3 and HuC/D to mark mitotic and postmitotic cells, respectively. TO-PRO-3 was also used to stain cell nuclei. Dashed line outlines the border of each tectal hemisphere. Scale bar represents 50 µm. A, anterior; L, lateral. (C-D) Quantification of the degree of proliferation and differentiation in the tectum. For the number of mitotic cells (PH-3$^+$) (C) 12.6 ± 1.4 for WT and 12.4 ± 1.2 for ast^{ti272z}; for the number of post-mitotic cells (HuC/D$^+$) (D) 119.1 ± 7.4 for WT and 113.6 ± 9.4 for ast^{ti272z}. All graphs show mean values ± SEM. ns, not significant, unpaired t-test.

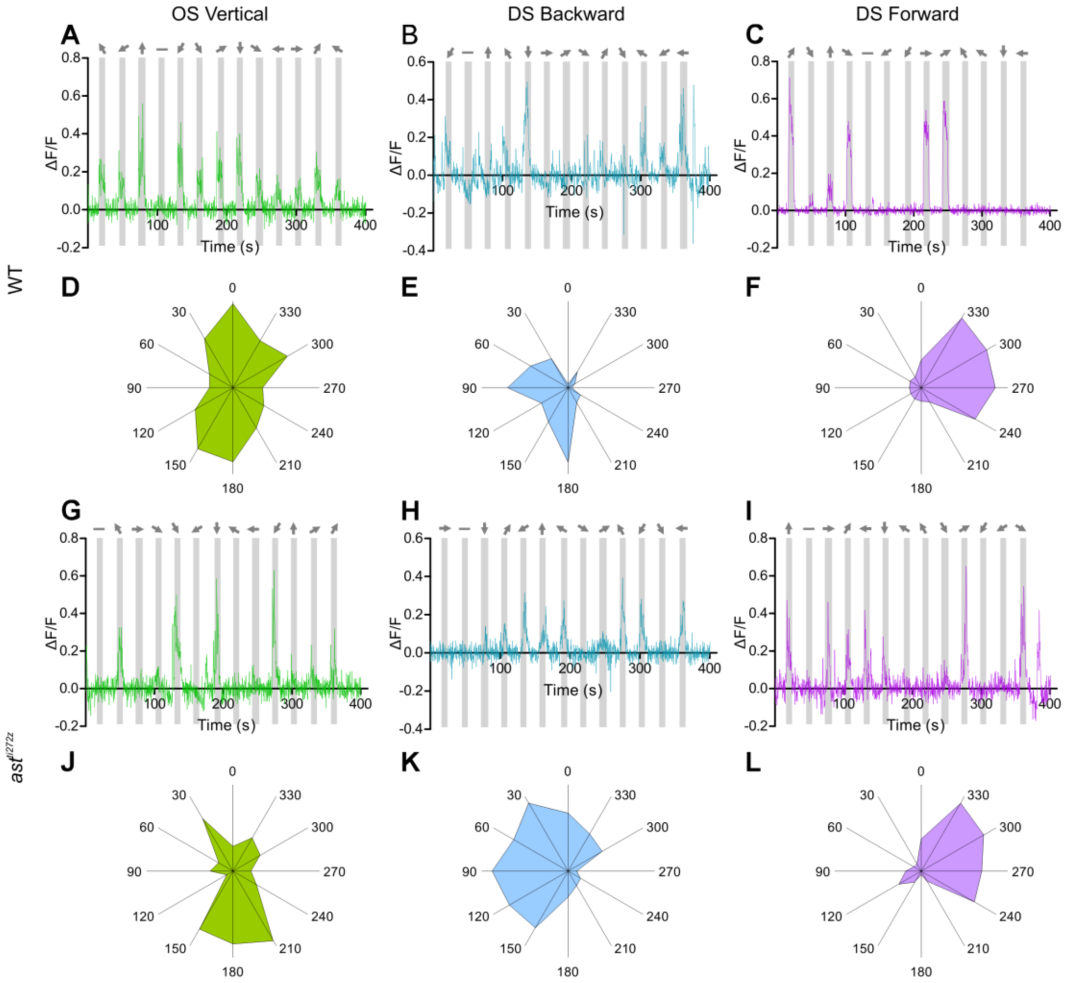

Figure S6. Responses of individual GCaMP6F-expressing FoxP2.A tectal cells (related to Figure 4). Examples of tuning experiments from individual FoxP2.A tectal cells in WT (A-F) and *ast*^{ti272z} (G-L) larvae at 7 dpf. OS vertical, DS backward and DS forward tuned cells from control and experimental groups are shown. (A, B, C, G, H, I) Cell body responses during a tuning experiment. Stimulus epochs are shown in gray and direction of motion is indicated by arrows on the top. (D, E, F, J, K, L) Integral responses are shown in polar plot form. Response traces and polar plots are color-coded accordingly to match the color-coding scheme used to describe the functional classes in Figure 4.

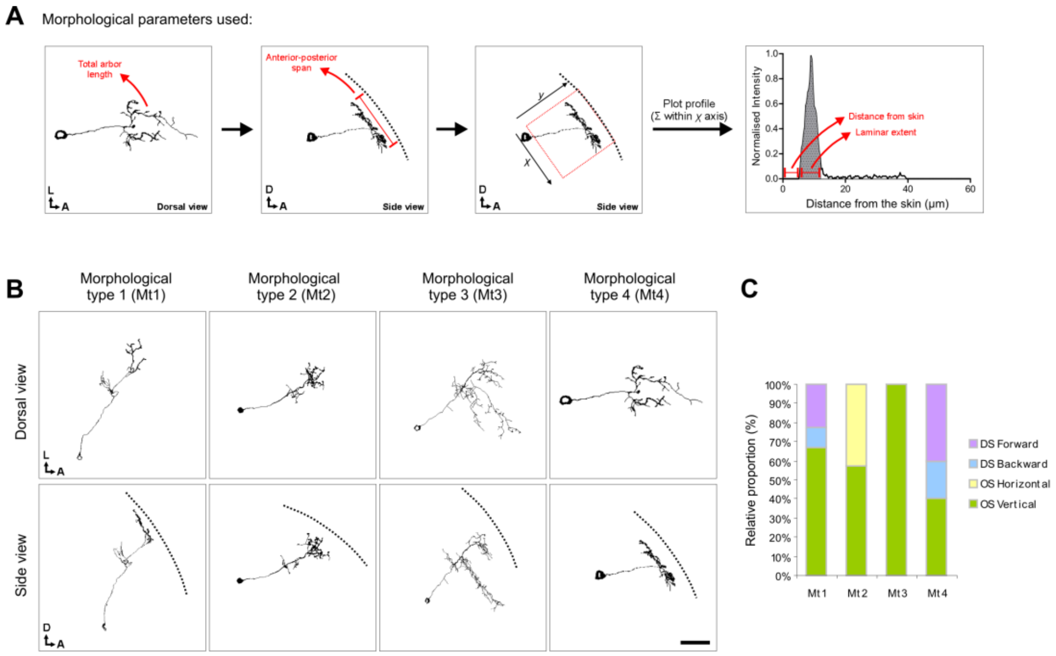

Figure S7. Morphological analysis of FoxP2.A labeled tectal cells (related to Figure 4). (A) Morphological parameters used to characterize FoxP2.A tectal cells and the process followed to extract them. Labeled tectal arbors were traced and total arbor length was calculated by summing the length of all individual traced fragments. Cells were reconstructed in three dimensions and then rotated to obtain an orthogonal view. From this view, an anterior-posterior span was measured. To obtain the other two parameters a rectangular region of interest was drawn across a relatively straight stretch of the tectal neuropil to cover the entire neurite. The fluorescence intensity across tectal depth was then calculated and the derived line plots were used to measure the distance from the skin and the laminar extent of distal arbors. A, anterior; D, dorsal; L, lateral. (B-C) Clustering of WT FoxP2.A responsive cells (n = 24 in 22 larvae) based on their morphological parameters was performed using k-means clustering. In total four morphological types are present (B), with each morphological cluster containing one or more functional subtypes (C). Scale bar represents 30 µm. A, anterior; D, dorsal; L, lateral.

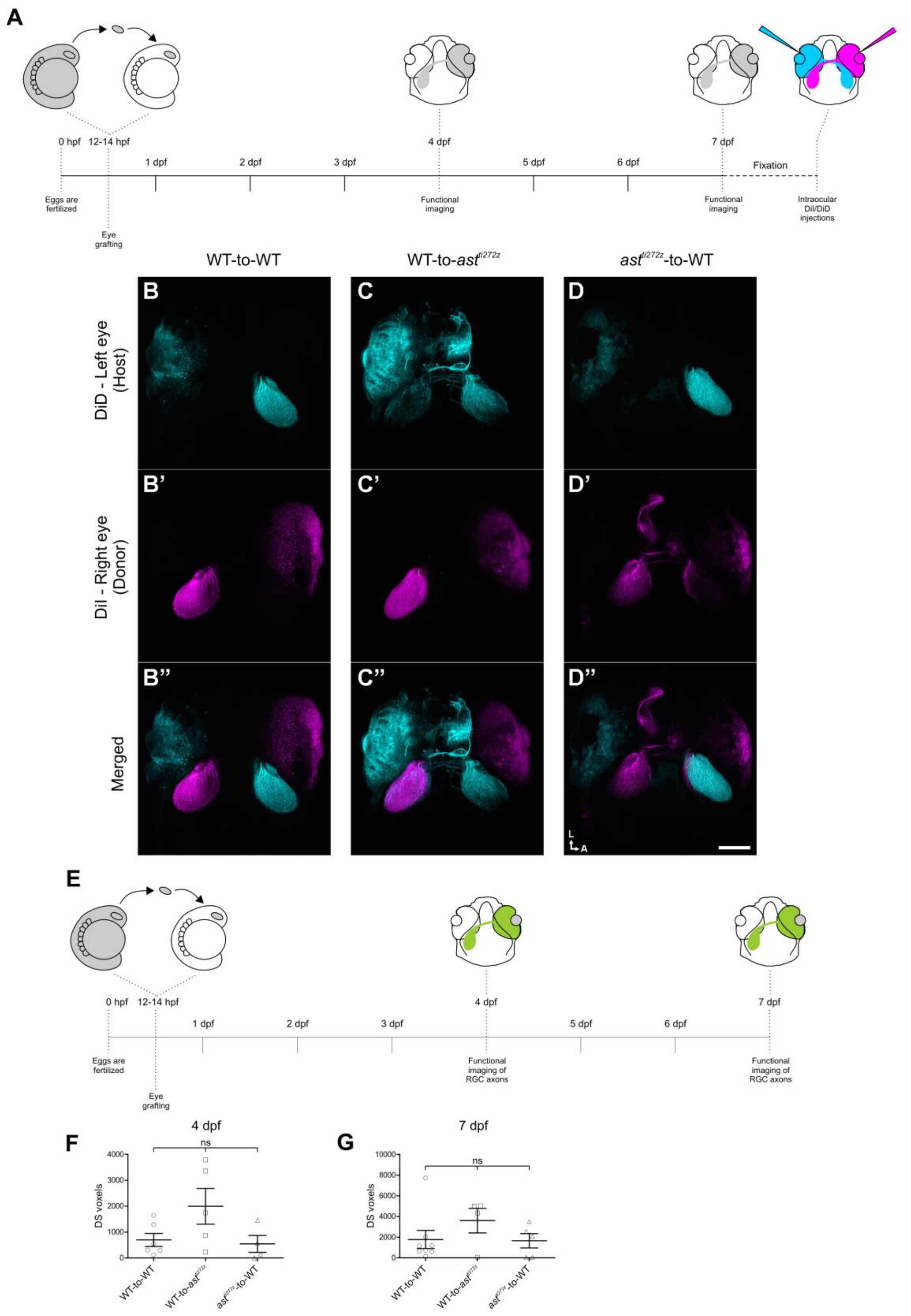

Figure S8. Eye transplantations as a means to assess the cellular requirements for Robo2 in the functional development of DS tectal cells (related to Figure 6). (A) Schematic showing the experimental procedure followed. Optic vesicles from donor embryos were transplanted into hosts at 12-14 hpf and functional imaging was performed at 4 and 7 dpf. To confirm the success of each transplantation experiment, samples were fixed post-functional imaging at

7dpf and the donor and host eyes were bulk-loaded with the lipophilic dyes, DiI and DiD respectively, in order to label the entire retinotectal projection. (B-D) Three transplantation examples (one per transplantation group) showing the retinotectal projection of host and donor eyes. Scale bar represents 100 µm. A, anterior; L, lateral. (E) Schematic showing the experimental procedure followed to investigate the functional development of donor DS-RGC axons. Optic vesicles from *Tg(Isl2b:Gal4;UAS:SyGCaMP3)* donor embryos were transplanted into hosts at 12-14 hpf and functional imaging of donor RGC axons within the tectum was performed at 4 and 7 dpf. (F-G) Quantification of average number of DS-RGC voxels per group. Total number of DS-RGC voxels at 4 dpf (F), 697 ± 252.6 for WT-to-WT (n = 6 larvae, total of 18 optical sections), 1996 ± 689.9 for WT-to-ast^{ti272z} (n = 5 larvae, total of 15 optical sections) and 544.3 ± 325.4 for ast^{ti272z}-to-WT (n = 4 larvae, total of 12 optical sections). Total number of DS cells at 7 dpf (G), 1783 ± 870.9 for WT-to-WT (n = 8 larvae, total of 24 optical sections), 3606 ± 1190 for WT-to-ast^{ti272z} (n = 4 larvae, total of 12 optical sections) and 1653 ± 692.9 for ast^{ti272z}-to-WT (n = 5 larvae, total of 15 optical sections). All graphs show mean values ± SEM. ns, not significant, Kruskal-Wallis and Dunn's multiple comparison tests.

	4 dpf			7 dpf		
	wt Mean ± SEM	*ast*$^{-/-}$ Mean ± SEM	*P* value	wt Mean ± SEM	*ast*$^{-/-}$ Mean ± SEM	*P* value
UPWARD **subpopulation**	56.5 ± 26.9	545.9 ± 257.1	0.095 ns	128.6 ± 39	455.7 ± 305.7	0.333 ns
DOWNWARD **subpopulation**	507.5 ± 191	316.2 ± 258.1	0.568 ns	618.1 ± 192.7	345.1 ± 139.6	0.261 ns
FORWARD **subpopulation**	3613 ± 667.4	2009 ± 705.4	0.121 ns	4259 ± 832.9	2067 ± 666.4	0.055 ns
TOTAL **population**	5237 ± 919	3624 ± 1283	0.333 ns	6409 ± 1266	4114 ± 1205	0.209 ns
UPWARD **bandwidth**	86.5 ± 8.8	85.3 ± 6	0.91 ns	92.9 ± 5.5	98.9 ± 6.1	0.483 ns
DOWNWARD **bandwidth**	98.3 ± 4.6	102.8 ± 12.5	0.703 ns	101.7 ± 4.3	90.7 ± 4.8	0.114 ns
FORWARD **bandwidth**	104.4 ± 1.8	92.6 ± 3	0.005 **	97 ± 1.6	95.3 ± 1.8	0.499 ns

Table S1. The functional response properties of DS-RGCs are normal in *astray* mutants (related to Figure 1). Comparison of the size of each DS subpopulation and their tuning bandwidth between WT (n = 8, total of 24 optical sections) and *ast*^{ti272z} (n = 9, total of 27 optical sections) larvae at 4 and 7 dpf. Values show mean ± SEM. **p < 0.01; ns, not significant, unpaired t-test.

	7 dpf		
	wt<br>Mean ± SEM	*ast*<br>Mean ± SEM	*P*<br>value
**UPWARD subpopulation**	1.3 ± 0.4	1.5 ± 0.8	0.801<br>ns
**BACKWARD subpopulation**	3.1 ± 1	3.3 ± 1.2	0.919<br>ns
**DOWNWARD subpopulation**	1.1 ± 0.4	1.2 ± 0.5	0.907<br>ns
**FORWARD subpopulation**	6.2 ± 1.5	8.7 ± 2.6	0.428<br>ns
**TOTAL population**	14.8 ± 2.9	18.1 ± 4.8	0.561<br>ns
**UPWARD bandwidth**	81.1 ± 5.1	97.6 ± 8.7	0.1141<br>ns
**BACKWARD bandwidth**	93.6 ± 5.4	94.5 ± 8	0.9294<br>ns
**DOWNWARD population**	90.6 ± 9	83.7 ± 7.7	0.5817<br>ns
**FORWARD bandwidth**	91.1 ± 2.3	86.5 ± 4.4	0.3456<br>ns

Table S2. The functional response properties of DS tectal cells in *astray* mutants are indistinguishable from WT animals at 7 dpf (related to Figure 3). Comparison of the size of each DS subpopulation and their tuning bandwidth between WT (n = 12, total of 36 optical sections) and *ast*^{ti272z} (n = 12, total of 36 optical sections) tecta at 7 dpf. Values show mean ± SEM. ns, not significant, unpaired t-test.

Video S1. Calcium responses of SyGCaMP3-expressing RGC axons in the WT optic tectum evoked by drifting bar stimulus (related to Figure 1). Tuning experiment from a *Tg(Isl2b:Gal4;UAS:SyGCaMP3)* larva (WT#6) summarized as a montage in Figure S1A. Raw fluorescence (left) and normalized ΔF/F (right) responses of SyGCaMP3-expressing RGC axons in the tectal neuropil evoked by drifting bar stimulus are shown. Movie encompasses an entire tuning experiment in which all 12 directions of bar motion are presented.

Video S2. Calcium responses of SyGCaMP3-expressing RGC axons in the *astray* optic tectum evoked by drifting bar stimulus (related to Figure 1). Tuning experiment from a *Tg(Isl2b:Gal4;UAS:SyGCaMP3)* larva (*ast*^{ti272z}#5) summarized as a montage in Figure S1D. Raw fluorescence (left) and normalized ΔF/F (right) responses of SyGCaMP3-expressing RGC axons in the tectal neuropil evoked by drifting bar stimulus are shown. Movie encompasses an entire tuning experiment in which all 12 directions of bar motion are presented.

Video S3. Calcium responses of GCaMP5G-expressing tectal neurons in the WT optic tectum evoked by drifting bar stimulus (related to Figure 3). Tuning experiment from a *Tg(elavl3:GCaMP5G)* larva (WT#2) summarized as a montage in Figure S4A. Raw fluorescence (left) and normalized ΔF/F (right) responses of GCaMP5G-expressing tectal cells in the tectum evoked by drifting bar stimulus are shown. Movie encompasses an entire tuning experiment in which all 12 directions of bar motion are presented.

Video S4. Calcium responses of GCaMP5G-expressing tectal neurons in the *astray* optic tectum evoked by drifting bar stimulus (related to Figure 3). Tuning experiments from a *Tg(elavl3:GCaMP5G)* larva (*ast*^{ti272z}#2) that are presented as a montage in Figure S4D. Raw fluorescence (left) and normalized ΔF/F (right) responses of GCaMP5G-expressing tectal cells in the tectum evoked by drifting bar stimulus are shown. Movie encompasses an entire tuning experiment in which all 12 directions of bar motion are presented.

Video S5. Calcium responses in a single GCaMP6F-expressing FoxP2.A tectal cell evoked by drifting bar stimulus (related to Figure 4). Time-lapse data showing the raw fluorescence (left) and normalized ΔF/F (right) responses of a single GCaMP6F-expressing tectal cell in the tectum evoked by drifting bar stimulus. Movie encompasses an entire tuning experiment in which all 12 directions of bar motion are presented.

Video S6. Three-dimensional reconstructions of FoxP2.A labeled tectal cells (related to Figure 4). Traced FoxP2.A tectal cells were reconstructed as shown. An example of a forward tuned DS tectal neuron in WT (top) and a*st*^{ti272z} (bottom) tectum is shown.

Supplemental Experimental Procedures

Animals

Zebrafish were reared at 28.5°C on a 14 hr light/10 hr dark cycle. Embryos produced by natural crosses were staged by hours, or days post-fertilization (hpf, or dpf) and raised in Danieau's solution (58 mM NaCl, 0.7 mM KCl, 0.4 mM $MgSO_4$, 0.6 mM $Ca(NO_3)_2$, 5.0 mM HEPES, pH 7.2). Transgenic lines utilized in this study include *Tg(Isl2b:Gal4)zc60* (Ben Fredj et al., 2010), *Tg(UAS:SyGCaMP3)kg1* (Nikolaou et al., 2012) and *Tg(elavl3:GCaMP5G)a4598* (Ahrens et al., 2013). The *robo2* mutant allele used in this study was ast^{ti272z} (Fricke et al., 2001). Functional imaging experiments were performed in the pigmentation mutant $mitfa^{w2}$, which lacks all neural crest derived melanophores (Lister et al., 1999). This work was approved by the local Animal Care and Use Committee (King's College London), and was carried out in accordance with the Animals (Experimental Procedures) Act, 1986, under license from the United Kingdom Home Office.

Generation of DNA Plasmid Constructs

To make the *FoxP2.A:Gal4FF* construct, a plasmid containing the 9.7kb *FoxP2-enhancerA* fragment (*p5EFoxP2.A*) (Bonkowsky et al., 2008), (provided by Joshua Bonkowsky, University of Utah), was digested with *BsrGI* and a basal promoter (containing the adenovirus *E1B TATA* box) was inserted to generate a *p5EFoxP2.Abas* plasmid. A LR reaction (Gateway cloning system, Invitrogen) was performed in order to fuse *FoxP2.Abas* with *Gal4FF* (*pMEGal4FF*) in a plasmid containing Tol2 sites (*pDestTol2R4R2pA*) (Villefranc et al., 2007), thus generating a *pTolFoxP2.Abas:Gal4FF* plasmid. To make the *5UAS:GCaMP6F* construct, the genetically encoded calcium indicator, *GCaMP6F* (Chen et al., 2013) was PCR amplified and directionally cloned into the *BglII/NotI* sites downstream of the upstream activator sequence (*UAS*) motifs of the *5UAS:EGFP-N2* plasmid (Ben Fredj et al., 2010) thus replacing the *EGFP* sequence.

DNA Electroporations

Tectal cells were mosaically labeled by co-electroporating the *FoxP2.A:Gal4FF* activator plasmid with effector plasmids, where expression of either GCaMP6F or tandem-dimer tomato (tdTomato) is driven by *5UAS* motifs (Ben Fredj et al., 2010). Electroporations were performed as previously described (Hoegler and Horne, 2010). Briefly, 4 dpf zebrafish larvae were mounted in 1% low melting point agarose (Sigma) in electroporation buffer (180 mM NaCl, 5 mM KCl, 1.8 mM $CaCl_2$, 5 mM HEPES, pH 7.2). Plasmid DNA was prepared using midi-prep kits (Qiagen) and pressure-injected into the midbrain ventricle at a concentration of 500 ng/µl each. Electroporation electrodes were positioned such that the positive electrode is lateral and slightly dorsal to the hemisphere of the optic tectum to be targeted and the negative electrode lateral and ventral to the opposite eye of the larvae. DNA plasmids were electroporated by delivering 1 second trains of 5 ms, 85 V voltage pulses at 200 Hz using an SD9 stimulator (Grass Instruments). A total of five trains per larva were delivered.

Enucleations

Embryos were collected and raised at 22°C until the 6-10 somite stage (12-14 hpf). Embryos were then mounted in 1.3% low melting point agarose (Sigma) in fish Ringer´s (116 mM NaCl,

3 mM KCl, 4 mM $CaCl_2$, 1 mM $MgCl_2$, 5 mM HEPES, 20 µg/ml gentamycin sulfate, pH 7.2). A small window was opened in the epidermis above the right eye primordia using a drop of mineral oil (Sigma), through which the optic vesicles can be reached. The optic vesicles were removed using sharpened tungsten micro-needles and a wire microloop. 1 hour after enucleation, the embryos were removed from the agarose and raised under standard conditions in Danieau's.

Eye transplantations

Eye transplantations were performed as previously described (Picker et al., 1999). Briefly, age-matched host and donor embryos were collected and raised at 22°C until the 6-10 somite stage (12-14 hpf). Host and donor embryos were mounted in 1.3% low melting point agarose (Sigma) in fish Ringer´s (116 mM NaCl, 3 mM KCl, 4 mM $CaCl_2$, 1 mM $MgCl_2$, 5 mM HEPES, 20 µg/ml gentamycin sulfate, pH 7.2). A small window was opened in the epidermis above the right eye primordia using a drop of mineral oil (Sigma), through which the optic vesicles can be reached. The host optic vesicle was removed and the donor optic vesicle inserted using sharpened tungsten micro-needles and a wire microloop. 1 hour after transplantation, when healing was complete, the embryos were removed from the agarose and raised under standard conditions in Danieau's. At the end of the experiment, the host and donor eyes were labeled with different lipophilic dyes (see DiI/DiD labeling below).

Visual stimulation and functional imaging

Non-anaesthetized larvae were immobilized in 2% low melting point agarose (Sigma) prepared in Danieau's solution and mounted dorsal side up on a raised glass platform that was placed in a custom-made Danieau-filled chamber. The agarose was sufficient to restrain the larvae so that anesthesia was not required. A diffusive filter 3026 (Rosco, Inc., Hollywood, CA) was bonded to one side of the chamber and which served as a projection screen. The agarose surrounding the eye facing the projection screen was removed allowing an unobstructed view of the projected image on the side of the chamber, while permitting the objective to be positioned above the tectum for imaging. Larvae were positioned 3 cm away from the screen and the projected image filled a visual field of approximately 97° by 63°. Visual stimuli consisted of light (56 cd/m^2) or dark bars (8 cd/m^2) (75% and 25% of mean respectively) on a mean grey background (32 cd/m^2). As no qualitative differences between light and dark bars were noted, data for the two stimuli are combined. Each bar was 10° in width moving at 20°/second and separated from the preceding bar by 30° - enabling more than one bar on the screen at any one time. The long axis of the bar was orthogonal to the direction of motion. Visual experiments were generated and controlled using custom written Labview and Matlab code (MathWorks) implemented on a ViSaGe stimulus presenter (Cambridge Research Systems, UK) and delivered via a DLP pico projector (Optoma). Functional imaging was performed using an LSM 710 confocal microscope equipped with a spectral detection scan head and a 20×/1.0 NA water-immersion objective (Carl Zeiss). Functional time-series of visually evoked SyGCaMP3 responses in RGCs were acquired at a rate of 4.1 Hz and 0.415 x 0.415 µm resolution, (256 x 256 pixels) and 1 A.U. pinhole aperture at three depths separated by 2 µm. The average diameter of a presynaptic bouton in zebrafish RGCs is ~0.8 µm (Meyer and Smith, 2006). Thus, the physical X-Y dimensions of voxels are below that of a typical presynaptic bouton. Imaging of visually evoked GCaMP

responses in tectal cells were acquired at a rate of 3.2 Hz and 0.66 x 0.66 µm resolution (256 x 256 pixels) and 1 AU pinhole at three depths separated by 7 µm (in the case of pan-neuronal GCaMP5G expression) and at the level of the cell body (in the case of single GCaMP6F expressing tectal cells). Excitation was provided by 488 nm multi-line laser. Occasionally we saw responses in isolated voxels at the onset of the laser, but generally no responses were observed unless visual stimuli were applied. Each orientation was presented once (8 seconds - 4 cycles) in a pseudo-random order unique to each slice in every animal imaged; each inter-epoch interval was 20 seconds to enable GCaMP signals to return to baseline and provide enough baseline to determine the knot for cubic spline interpolation of the baseline signals (see next section). A blank screen null condition of 8 seconds was also interleaved.

Voxel-wise analysis

The confocal time-series were pre-processed prior to analysis as follows: time-series images from each experiment were corrected for motion with a rigid-body algorithm (spm8 - www.fil.ion.ucl.ac.uk/spm); median filtered with a kernel size of 1 voxel (0.415 µm) to remove dark and shot noise; spatially smoothed with a 2D Gaussian kernel = 2 voxels (0.83 µm) to improve signal-to-noise and; low-frequency drifts in baseline corrected using a cubic-spline algorithm extrapolating between knots averaged from 5 seconds of the inter-epoch interval data (~21-22 time-frames). Reference anatomical images were derived from the average of images at this stage of pre-processing. Normalized signal intensity changes (% ΔF/F) were calculated on the detrended time-series at each voxel and the integral response over the epoch-interval calculated to provide a single response metric of each presented orientation. Briefly, a threshold for each voxel within an acquisition image was determined from the normalized signal intensity (% ΔF/F) changes during the inter-epoch intervals and null condition, threshold = 5× standard deviations. All voxels that were suprathreshold within at least two visually presentation epochs were regarded as visually responsive and analyzed for direction- and orientation-selectivity. Direction- and orientation-selective indices (DSI and OSI) (Niell and Stryker, 2008), based on fitted von-Mises profiles (Swindale, 1998), were calculated together with an estimate for their goodness of fit - R^2 (Lowe et al., 2013). To minimize cross talk and overfitting associated with DSI and OSI metrics, a stringent approach was undertaken. For a voxel to be regarded as DS mutually exclusive criteria were used: DS if DSI > 0.5 and OSI < 0.5. The goodness of fit for DSI and OSI, respectively, had to be greater than 0.8; thus, the fitted curves explained at least 80% of the integral responses. The fitted curves used to estimate DSI provided an estimate of the preferred direction of motion from the center of the fitted curve. For automatic segmentation of tectal cells (Hunter et al., 2013), DS voxels were spatially aggregated into islands of like responses whose spatial extent was 10-50 voxels (~7-33 µm^2 – the approximate size limits of a cell soma at this age). The median preferred angle of all constituent voxels was taken as the preferred angle of the cluster.

Identifying functional subtypes of DS-RGC and tectal cells

To derive the number of subtypes of DS-RGCs and tectal cells, grouped cumulative histograms of the preferred direction of motion were constructed from the calculated preferred angles. Multiple von-Mises distributions were summed and fitted to the

cumulative histograms using a multidimensional constrained nonlinear minimization approach, with peak-center, height, concentration, and base height being free dimensions. In all cases, the residuals of the fitted summed distributions were inspected for missed coherent populations. Three and four individual distributions were summed for the RGC and tectal cell cumulative histograms respectively. In both cases no coherent residuals remained. Populations of DS functional subtypes were determined by band-limiting responsive voxels or cells to those within ± 2× the bandwidth of each fitted von-Mises-derived population peak.

Constructing standard tectal neuropil space

Composite maps of direction-selective responses were generated by rigid-body co-registration of the pre-processed derived reference anatomical images to the middle slice of an arbitrarily chosen fish (spm8 - www.fil.ion.ucl.ac.uk/fil/spm). A mean image of the co-registered images was calculated which then became a template that represented the anatomy of the group unbiased by any individual. All slices from all animals were then finally co-registered to this template. Great care was taken to acquire approximately the same slice in the tectal neuropil in all animals by prior imaging the whole neuropil and finding its dorsal and caudal poles. The translations for each imaged slice to the template atlas slice were applied to the threshold direction-selective maps. Individual maps were constructed by summing the incidence of DS functional subtypes across all slices. Composite maps for direction-selective voxels were then derived by color-coding for each respective subpopulation. Rotating the template and composite maps enabled these maps to be collapsed into summary line-plots using the Plot_Profile tool in ImageJ (NIH).

Morphological analysis of tectal cells

Electroporated GCaMP6F- and tdTomato-positive tectal cells were imaged as above. Excitation was 488 nm (for GCaMP6F) or 543 nm (for tdTomato). Image stacks of labeled cells were captured at a 0.33 x 0.33 µm resolution, (512 x 512 pixels) and 1 A.U. pinhole aperture with 0.7 µm sectioning z-interval. Datasets were opened in ImageJ2 (NIH) and tectal arbors were traced using the Simple_Neurite_Tracer plugin. Neurite traces were then further analyzed in ImageJ (NIH) to acquire their morphological parameters: total arbor length, distal arbor A-P span, distance from skin, and distal arbor laminar extent. To obtain the last two parameters the cells were reconstructed in three dimensions and rotated to obtain an orthogonal view. A rectangular region of interest was drawn across a relatively straight stretch of the tectal neuropil to cover the entire neurite. The Plot_Profile tool in ImageJ (NIH) was applied to the rectangle to calculate the fluorescence intensity across tectal depth. Line plots generated were normalized and analyzed using the Prism 5 (GraphPad) 'Smooth' and 'Area Under Curve' functions to measure distance from skin and distal arbor laminar extent (Figure S7A). Distance from skin and distal arbor laminar extent were based on the maximum width of the normalized line plot - a method previously used to characterize tectal arbor morphology (Robles et al., 2011). k-means clustering was performed using the above parameters. For cluster analysis, each data parameter was standardized by diving each value with the population mean. The mean silhouette value of 1-6 clusters was calculated to determine the optimum number of clusters.

DiI/DiD labeling

Retinal projections were anterogradely labeled by mounting paraformaldehyde-fixed larval zebrafish in 1% low melting point agarose (Sigma), and pressure-injecting a 10 mg/ml ethanol solution of the carbocyanine dyes DiI or DiD (Biotium) between the lens and neural retina to label the entire RGC layer. After an overnight diffusion period, DiI/DiD labeled axons were imaged as above. Excitation was 514 nm (for DiI) and 633 nm (for DiD). Image stacks were captured at a 1.18 x 1.18 µm resolution, (512 x 512 pixels) and 1 A.U. pinhole aperture with a 2 µm sectioning z-interval.

Analysis of tectal proliferation and differentiation

To analyze the degree of proliferation and differentiation in the tectum, whole-mount immunohistochemistry was performed on 48 hpf larvae as previously described (Hunter et al., 2011). Anti-PH-3 (Millipore) and anti-HuC/D (Life Technologies) antibodies were used at 1:350 and 1:100 respectively and the detection of primary antibodies was carried out using Alexa Fluor 488, 546 goat anti-rabbit or anti-mouse conjugates (1:500, Life Technologies). TO-PRO-3 (Life Technologies) was used at 1:1000 to stain cell nuclei. Confocal imaging was performed as above. Image stacks were captured at a 0.27 x 0.27 µm resolution, (1024 x 1024 pixels) and 1 A.U. pinhole aperture at several depths separated by 3 µm. The amount of proliferation and differentiation in the tectum was calculated by scoring the number of mitotic (PH-3$^+$) and postmitotic (HuC/D$^+$) cells, respectively, per tectal hemisphere (in a middle dorso-ventral section of the tectum).

Statistical analyses

Kolmogorov-Smirnov, D’Agostino and Pearson, and Shapiro-Wilk tests followed by an unpaired t-test or Mann Whitney test, as appropriate, were performed. For multiple comparisons, a Kruskal-Wallis and Dunn's multiple comparison test was used. The criterion for statistical significance was set at $p < 0.05$ and results are represented as mean ± SEM. All analyses were performed using Prism 5 (GraphPad).

Supplemental References

Ahrens, M.B., Orger, M.B., Robson, D.N., Li, J.M., and Keller, P.J. (2013). Whole-brain functional imaging at cellular resolution using light-sheet microscopy. Nature methods *10*, 413-420.

Ben Fredj, N., Hammond, S., Otsuna, H., Chien, C.B., Burrone, J., and Meyer, M.P. (2010). Synaptic activity and activity-dependent competition regulates axon arbor maturation, growth arrest, and territory in the retinotectal projection. J Neurosci *30*, 10939-10951.

Bonkowsky, J.L., Wang, X., Fujimoto, E., Lee, J.E., Chien, C.B., and Dorsky, R.I. (2008). Domain-specific regulation of foxP2 CNS expression by lef1. BMC developmental biology *8*, 103.

Chen, T.W., Wardill, T.J., Sun, Y., Pulver, S.R., Renninger, S.L., Baohan, A., Schreiter, E.R., Kerr, R.A., Orger, M.B., Jayaraman, V.*, et al.* (2013). Ultrasensitive fluorescent proteins for imaging neuronal activity. Nature *499*, 295-300.

Fricke, C., Lee, J.S., Geiger-Rudolph, S., Bonhoeffer, F., and Chien, C.B. (2001). astray, a zebrafish roundabout homolog required for retinal axon guidance. Science *292*, 507-510.

Hoegler, K.J., and Horne, J.H. (2010). Targeting the zebrafish optic tectum using in vivo electroporation. Cold Spring Harbor protocols *2010*, pdb prot5463.

Hunter, P.R., Lowe, A.S., Thompson, I.D., and Meyer, M.P. (2013). Emergent properties of the optic tectum revealed by population analysis of direction and orientation selectivity. J Neurosci *33*, 13940-13945.

Hunter, P.R., Nikolaou, N., Odermatt, B., Williams, P.R., Drescher, U., and Meyer, M.P. (2011). Localization of Cadm2a and Cadm3 proteins during development of the zebrafish nervous system. J Comp Neurol *519*, 2252-2270.

Lister, J.A., Robertson, C.P., Lepage, T., Johnson, S.L., and Raible, D.W. (1999). nacre encodes a zebrafish microphthalmia-related protein that regulates neural-crest-derived pigment cell fate. Development *126*, 3757-3767.

Lowe, A.S., Nikolaou, N., Hunter, P.R., Thompson, I.D., and Meyer, M.P. (2013). A Systems-Based Dissection of Retinal Inputs to the Zebrafish Tectum Reveals Different Rules for Different Functional Classes during Development. J Neurosci *33*, 13946-13956.

Meyer, M.P., and Smith, S.J. (2006). Evidence from in vivo imaging that synaptogenesis guides the growth and branching of axonal arbors by two distinct mechanisms. J Neurosci *26*, 3604-3614.

Niell, C.M., and Stryker, M.P. (2008). Highly selective receptive fields in mouse visual cortex. J Neurosci *28*, 7520-7536.

Nikolaou, N., Lowe, A.S., Walker, A.S., Abbas, F., Hunter, P.R., Thompson, I.D., and Meyer, M.P. (2012). Parametric functional maps of visual inputs to the tectum. Neuron *76*, 317-324.

Picker, A., Brennan, C., Reifers, F., Clarke, J.D., Holder, N., and Brand, M. (1999). Requirement for the zebrafish mid-hindbrain boundary in midbrain polarisation, mapping and confinement of the retinotectal projection. Development *126*, 2967-2978.

Robles, E., Smith, S.J., and Baier, H. (2011). Characterization of genetically targeted neuron types in the zebrafish optic tectum. Frontiers in neural circuits *5*, 1.

Swindale, N.V. (1998). Orientation tuning curves: empirical description and estimation of parameters. Biological cybernetics *78*, 45-56.

Villefranc, J.A., Amigo, J., and Lawson, N.D. (2007). Gateway compatible vectors for analysis of gene function in the zebrafish. Developmental dynamics : an official publication of the American Association of Anatomists *236*, 3077-3087.
