# Supplementary material for: Lamination Speeds the Functional Development of Visual Circuits
Source: Neuron. 2015 Dec 2;88(5):999–1013. doi: 10.1016/j.neuron.2015.10.020 (PMC4674658; doi:10.1016/j.neuron.2015.10.020)
Supplement: Document S2. Article plus Supplemental Information [file mmc8.pdf]

Article

Neuron

Lamination Speeds the Functional Development of Visual Circuits

Highlights

- Robo2 directs lamination of direction-selective retinal axons and tectal dendrites
- Tectal lamination is required for rapid assembly of direction-selective circuits
- Functional direction-selective circuits eventually form when lamination is lost
- Structural plasticity compensates for the loss of tectal lamination

Authors

Nikolas Nikolaou, Martin P. Meyer

Correspondence

nikolas.nikolaou@kcl.ac.uk (N.N.),
martin.meyer@kcl.ac.uk (M.P.M.)

In Brief

A feature of the brain is the arrangement of synapses in layers. The significance of this architecture is unknown. Nikolaou and Meyer show that lamination is ultimately dispensable for circuit function but that it is crucial for rapid circuit development.

Nikolaou & Meyer, 2015, Neuron *88*, 999–1013
December 2, 2015 ©2015 The Authors
http://dx.doi.org/10.1016/j.neuron.2015.10.020

Lamination Speeds the Functional Development of Visual Circuits

Nikolas Nikolaou[1,*] and Martin P. Meyer[1,*]
[1]MRC Centre for Developmental Neurobiology, King's College London, Guy's Hospital Campus, London, SE1 1UL, UK
*Correspondence: nikolas.nikolaou@kcl.ac.uk (N.N.), martin.meyer@kcl.ac.uk (M.P.M.)
http://dx.doi.org/10.1016/j.neuron.2015.10.020
This is an open access article under the CC BY license (http://creativecommons.org/licenses/by/4.0/).

SUMMARY

A common feature of the brain is the arrangement of synapses in layers. To examine the significance of this organizational feature, we studied the functional development of direction-selective (DS) circuits in the tectum of *astray* mutant zebrafish in which lamination of retinal ganglion cell (RGC) axons is lost. We show that although never laminar, the tuning of DS-RGC axons targeting the mutant tectum is normal. Analysis of mutant tectal neurons at late developmental stages reveals that directional tuning is indistinguishable from wild-type larvae. Furthermore, we show that structural plasticity of tectal dendrites and RGC axons compensates for the loss of lamination, establishing connectivity between DS-RGCs and their normal tectal targets. However, tectal direction selectivity is severely perturbed at earlier developmental stages. Thus, the formation of synaptic laminae is ultimately dispensable for the correct wiring of direction-selective tectal circuits, but it is crucial for the rapid assembly of these networks.

INTRODUCTION

A common feature of the CNS is the concentration of synapses in layers or laminae. Axons and dendrites are not randomly distributed among different laminae. Instead, a particular lamina will contain axons and dendrites arising from specific neuronal subtypes resulting in the formation of laminae that contain synapses with the same or similar functional properties. For example, the retina and tectum/superior colliculus contain multiple synaptic laminae stacked on top of one another and a lamina's output will often represent one visual feature such as motion in a particular direction (Baier, 2013; Dhande and Huberman, 2014; Sanes and Zipursky, 2010). While there is now a wealth of experimental evidence showing that the generation of layers can be achieved through multiple cellular and molecular mechanisms (Sanes and Yamagata, 2009), the purpose of layers themselves is not known. One possibility is that laminar-specific targeting of axons and dendrites is a necessary step in the development of synapses between specific neuronal subtypes. In this scenario, laminae are a by-product of the developmental mechanisms that establish wiring specificity in the brain. An alternative idea is that laminae may represent a fundamental unit of brain function, clustering synapses with similar functional properties and segregating synapses whose functional properties differ. This organizing principle may be vital for processing and integration of information in postsynaptic circuits. However, studies of *reeler* mutant mice, in which cortical lamination is lost, show only minor abnormalities in electrophysiological properties of single cortical neurons and sensory map organization and stimulus representation in the barrel and visual cortex are largely intact in these mutants (Dräger, 1981; Guy et al., 2015; Lemmon and Pearlman, 1981; Simmons and Pearlman, 1983). These findings suggest that subdivision of cortical networks into layers may not be required to establish wiring specificity or cortical function. A third explanation for the existence of layered neural architecture is wiring economy, the tendency to minimize axon and dendrite lengths. Wiring minimization has successfully explained the individual placement of neurons in simple nervous systems like that of *C. elegans* and *Drosophila melanogaster*, as well as the spatial organization of coarser structures like cortical areas in the mammalian brain, but it has yet to explain the organization of synaptic connections into layers (Chklovskii and Koulakov, 2004; Pérez-Escudero and de Polavieja, 2007; Rivera-Alba et al., 2011). Thus, there is currently little experimental or theoretical data demonstrating a role for layers in the brain.

Here we use the optic tectum of larval zebrafish to investigate the significance of a layered neural architecture. The tectum is involved in directing the head and eyes to particular locations in visual space and receives input from upward of 20 different RGC subtypes, each responding best to a specific feature in the visual scene. In the zebrafish tectum, RGC axons project to a neuropil composed of four major layers that, from the most superficial to the deepest, are named stratum opticum (SO), stratum fibrosum et griseum superficiale (SFGS), stratum griseum centrale (SGC), and a lamina at the interface between the stratum album centrale and the stratum periventriculare (SAC/SPV) (Burrill and Easter, 1994). The majority of retinal inputs target SFGS, which is further divided into at least six sublaminae (Robles et al., 2013). As a rule, a single RGC axon will only innervate a single sublamina and the laminar specificity with which RGC axons innervate the tectum is precise from the earliest developmental stages (Robles et al., 2013). Functional analysis of direction- and orientation-selective RGCs shows that, at least for these functional classes, precise targeting of axons can generate stereotypic and functionally specialized sublaminae within the tectum. For example, RGCs tuned

to upward-, downward-, and forward-directed motion specifically target superficial sublaminae within SFGS (Gabriel et al., 2012; Lowe et al., 2013; Nikolaou et al., 2012). Within the population of tectal neurons are subtypes similarly selective for up, down, and forward motion as well as a subtype selective for backward motion—an emergent property of tectal circuits (Gabriel et al., 2012; Grama and Engert, 2012; Hunter et al., 2013; Niell and Smith, 2005). For forward-selective tectal neurons, tuning can be predicted from the retinal input layer in which their dendrites stratify (Gabriel et al., 2012). Thus, the precise targeting of RGC axons channels information about specific visual features to particular sublaminae and selective sublaminar targeting of tectal dendrites provides a simple mechanism by which retinal information is sampled and integrated by tectal neurons.

How is the functional development of direction-selective (DS) connections between RGCs and tectal neurons affected when lamination of RGC axons is lost? To answer this, we took advantage of the *astray* (ast^{ti272z}) mutant zebrafish in which the laminar arrangement of retinal inputs to the tectum is severely disrupted. The ast^{ti272z} allele is a functional null mutation of *robo2*—part of an evolutionarily conserved group of transmembrane proteins that, together with their Slit ligands, have been shown to be important for long-range axon guidance, growth, and targeting (Campbell et al., 2007; Fricke et al., 2001; Xiao et al., 2011). Targeting errors of RGC axons within the ast^{ti272z} tectum are thought to arise from the failure of mutant axons to read a superficial-to-deep gradient of Slit1a (Xiao et al., 2011). The functional consequences of these targeting errors are unknown. Using in vivo functional imaging of RGC axons in the ast^{ti272z} mutant tectum we show that, although never laminar, the tuning of DS-RGCs is normal. Strikingly, the tuning properties of individual tectal neurons and population encoding of directional motion in the mutant tectum are indistinguishable from that seen in wild-type larvae at late stages of development. Furthermore, we show that structural plasticity of tectal dendrites or RGC axons overcomes the loss of lamination cues, enabling functional and cell-type-specific connections to form between DS-RGCs and their normal tectal neuron targets. However, at early stages of development, direction selectivity in the tectal population is significantly perturbed. Thus, our results show that a laminar tectal architecture is ultimately dispensable for the functional development of direction-selective circuits but that lamination is essential for the rapid assembly of these neural networks.

RESULTS

Functional Properties of DS-RGCs Targeting the Tectum Are Normal in *astray* Mutants

Functional properties of RGCs and tectal neurons have not been studied in the *astray* (ast^{ti272z}) mutant. Therefore, before determining how tectal circuits devoted to processing of motion were altered in animals with disrupted laminar input, we first determined whether direction-selective circuits are established correctly in the mutant retina. We did this by generating wild-type (WT) and ast^{ti272z} larvae expressing a presynaptically targeted, genetically encoded reporter of neural activity specifically in RGCs (*Tg(Isl2b:Gal4;UAS:SyGCaMP3)*), and recording visually evoked activity in presynaptic terminals of RGC axons within the intact tectum (Nikolaou et al., 2012). Drifting bars moving in each of 12 different directions were presented to one eye of *Tg(Isl2b:Gal4;UAS:SyGCaMP3)* larvae while visually evoked responses were imaged in the contralateral tectum (Movies S1 and S2). To characterize directional tuning in RGC axons, we employed an analysis strategy developed previously that permits direction selectivity to be determined on a voxel-by-voxel basis, but also describes how the direction of motion is encoded on a population basis. The integral response of each voxel during each stimulus epoch was calculated and this was then used to generate tuning curves for each voxel (Figures S1A–S1F). Direction- and orientation-selective indices (DSI and OSI) of all visually responsive voxels were calculated based on fitted von-Mises profiles, together with an estimate for goodness of fit, R^2. For a voxel to be regarded as DS, mutually exclusive criteria were employed: DS if DSI > 0.5, OSI < 0.5, and the R^2 for DSI and OSI had to be greater than 0.8 (Lowe et al., 2013) (see Experimental Procedures). The center of the fitted curves used to estimate DSI was also used to provide an estimate of the preferred direction of motion. The WT and ast^{ti272z} larvae were imaged at two stages of development—4 days post-fertilization (4 dpf), which is approximately 24 hr after the first RGC axons enter the tectum, and 7 dpf when synapses between RGC axons and tectal dendrites and visually evoked behaviors are well established (Fleisch and Neuhauss, 2006; Meyer and Smith, 2006; Niell et al., 2004). The preferred angles of DS voxels from all imaged larvae were used to generate a population histogram for each condition (WT and ast^{ti272z} larvae imaged at 4 and 7 dpf). These reveal distinct distributions of preferred angles that are strikingly similar in WT and mutant tecta (Figures 1A–1D). Moreover, by iteratively fitting three summed von-Mises distributions to the population histograms (overlaid color curves in Figures 1A–1D), we found that there are three, largely non-overlapping, DS subpopulations with peaks centered at very similar locations in WT and mutant larvae. Location of peak centers across the four experimental conditions ranged from 22°–28° (upward motion), 138°–178° (downward motion), and 253°–268° (forward motion) (color-coded arrows in Figures 1A–1D). To estimate the relative proportions of each DS functional subtype, we band-limited responsive voxels to those within two times the bandwidth of each fitted von-Mises distribution. We found that the number of voxels of each DS subtype and the total number of DS voxels are not significantly different in ast^{ti272z} and WT larvae at either 4 or 7 dpf (Figures 1E and 1F). To test whether the response properties of DS subtypes differ between the two experimental groups, we generated polar plots illustrating the mean (±1 SD) normalized response profile for each DS subtype (insets in Figures 1A–1D). These illustrate that in both WT and ast^{ti272z} larvae, the three DS-RGC subtypes fill direction space and exhibit a triangular organization. Furthermore, we found that in both ast^{ti272z} and WT tecta DS-RGC tuning is precise from 4 dpf and that tuning bandwidths of DS-RGC subtypes are very similar in WT and ast^{ti272z} larvae (Table S1). These data show that in WT larvae the tuning and development of DS-RGCs are the same as that described previously (Lowe et al., 2013; Nikolaou et al., 2012) and that the functional development of DS-RGCs is normal in ast^{ti272z} mutants.

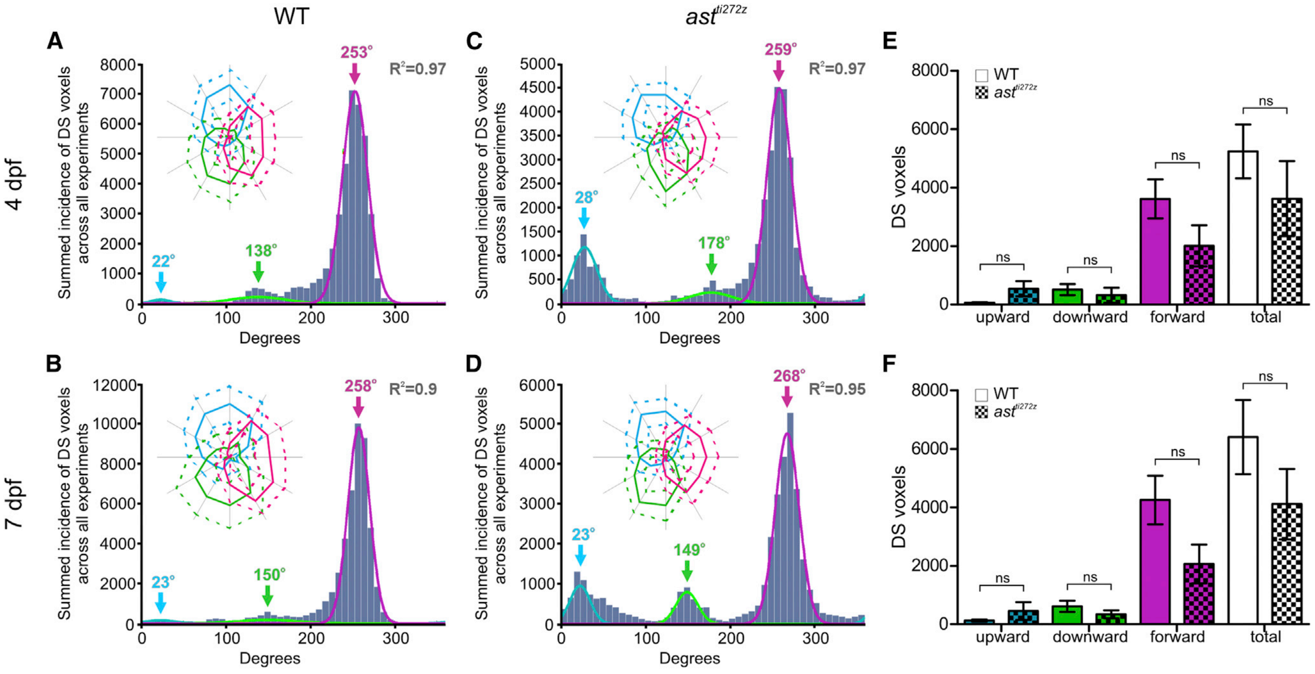

Figure 1. Tuning of DS-RGCs Innervating the Tectum Is Normal in *astray* Mutants

Functional imaging in WT (n = 8, total of 24 optical sections) and *ast*^{ti272z} (n = 9, total of 27 optical sections) larvae expressing the presynaptic reporter of neural activity, SyGCaMP3, specifically in RGC axons (*Tg(Isl2b:Gal4;UAS:SyGCaMP3)*).

(A–D) Cumulative histograms summarizing the incidence of preferred angles of all DS voxels in WT larvae at 4 dpf (A) and 7 dpf (B), and *ast*^{ti272z} larvae at 4 dpf (C) and 7 dpf (D). Overlaid colored curves show fitted von-Mises distributions used to identify three functional subtypes of DS-RGC. Color-coded arrows represent peak preferred angles of each subtype. R^2 values relate to the summed von-Mises distributions. Insets show polar plots of the mean (solid line) ± 1 SD (dashed line) normalized response profile for voxels of each DS-RGC subtype.

(E and F) Quantification of DS voxel numbers shows no significant difference between WT and *ast*^{ti272z} RGCs at 4 dpf (E) or 7 dpf (F). All graphs show mean values ± SEM. ns, not significant, unpaired t test. See also Figure S1, Table S1, Movies S1 and S2.

Lamination of DS-RGCs Is Perturbed in the *astray* Mutant Tectum

To examine how the targeting of axons of DS-RGCs is altered in the *ast*^{ti272z} mutant, we spatially co-registered data from all imaged larvae in each experimental condition to generate composite functional maps of DS-RGC axons in WT and mutant tecta (see Experimental Procedures). The panels in Figures 2A–2D are color coded for the three DS-RGC subtypes and in each parametric map, voxel brightness is proportional to the summed incidence of each functional subtype across all larvae. In WT animals, responses of DS-RGC subtypes are confined to superficial sublaminae within SGFS throughout development (Figures 2A and 2B) (Lowe et al., 2013; Nikolaou et al., 2012). In contrast, we found that the laminar functional architecture of DS-RGC subtypes in *ast*^{ti272z} larvae is severely disrupted at both 4 and 7 dpf, as would be expected from previous morphological studies of RGC axons in the *ast*^{ti272z} retinotectal projection (Xiao et al., 2011). Specifically, responses of DS-RGC subtypes are diffusely distributed across SFGS in *ast*^{ti272z} mutants (Figures 2C and 2D), a pattern that is also evident in functional maps generated from single larvae (compare Figures S2A and S2B with S2C and S2D). To further examine the distribution of DS-RGC responses in the mutant tectum, we used the composite maps to generate line plots showing the summed incidence of each functional subtype across an axis that represents the laminar organization of the tectal neuropil. In WT animals, the three DS-RGC subtypes at each age exhibit a sublaminar organization with the most abundant subtype (forward motion, magenta) found in a superficial sublamina, whilst the subtypes selective for upward and downward motion occupy a slightly deeper sublamina (Figures 2E and 2F) (Lowe et al., 2013; Nikolaou et al., 2012). In contrast, the laminar organization of DS-RGC subtypes in *ast*^{ti272z} animals is lost (Figures 2G and 2H). While lamination may be lost, the relative ordering of DS-RGC subtypes across the depth of the tectum may still be intact. To examine this, we performed pairwise comparisons of the spatial distributions of each subtype within WT and *ast*^{ti272z} larvae at 4 dpf (Figures 2I–2N) and 7 dpf (Figures S3G–S3L). This reveals that the degree of overlap between subtypes is increased in the mutant and that the relative order of functional subtypes across the laminar axis of the tectum is perturbed in the mutants. For example, in wild-type tecta, voxels tuned to forward motion always occupy the most superficial locations at both 4 and 7 dpf. In the mutant tectum, voxels tuned to upward motion invade the territory normally only occupied by voxels tuned to forward motion (compare Figure 2J with 2M and Figure S3H with S3K). Similarly, voxels tuned to upward and downward occupy the deepest locations in wild-type larvae but in the mutant voxels selective for forward motion invade these locations (for example, compare Figure S3I with S3L). Thus, lamination and the relative ordering of DS-RGC subtypes are perturbed in the

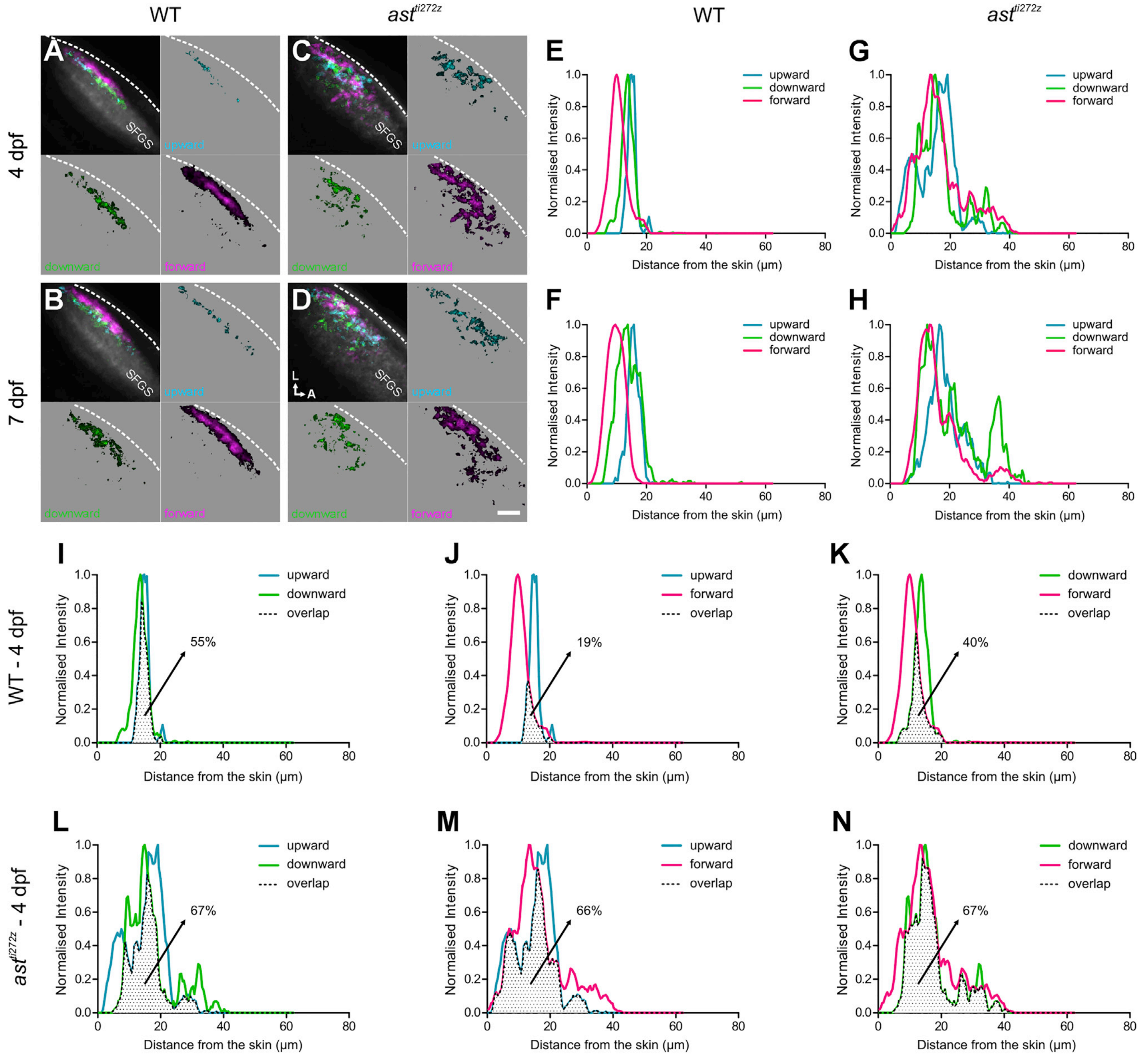

Figure 2. Lamination of DS-RGC Axons in the Tectum Is Lost in *astray* Mutants

(A–D) Composite parametric maps generated from all *Tg(Isl2b:Gal4;UAS:SyGCaMP3)* fish imaged showing the spatial distribution of DS-RGC subtypes in the tectal neuropil of WT larvae (n = 8, total of 24 optical sections) at 4 dpf (A) and 7 dpf (B), and *ast*^{ti272z} larvae (n = 9, total of 27 optical sections) at 4 dpf (C) and 7 dpf (D). Voxel brightness is proportional to the summed incidence of each functional subtype across all larvae imaged. The standard space template image derived from the mean fluorescence image of SyGCaMP3-expressing axons (grayscale) provides an anatomical reference. Dashed lines indicate the position of the skin overlaying the tectum. Scale bar represents 20 μm. A, anterior; L, lateral; SFGS, stratum fibrosum et griseum superficiale.

(E–H) Line plots generated from the composite parametric maps in (A)–(D) illustrating the distribution of DS-RGC subtypes along the laminar axis of the tectum in WT larvae at 4 dpf (E) and 7 dpf (F), and *ast*^{ti272z} larvae at 4 dpf (G) and 7 dpf (H). Line plots show normalized intensity of each DS-RGC subtype as a function of its distance from the skin.

(I–N) Pairwise comparisons showing the degree of spatial overlap between upward and downward (I and L), upward and forward (J and M), and downward and forward (K and N) DS-RGC subtypes within WT (I–K) and *ast*^{ti272z} (L–N) tecta at 4 dpf. Dotted area represents the area of intersection between the two subtypes. Values shown represent the fraction of downward (for I and L) or forward (for J, K, M, and N) DS voxels that spatially overlap with upward or downward DS voxels. See also Figures S2 and S3.

mutant. However, a bias toward the superficial half of SFGS is nevertheless maintained in the mutant tectum, suggesting mechanisms that establish a coarse organization of retinal afferents are present in the *ast*^{ti272z} tectum. By overlaying the line plots for each DS subtype from WT and *ast*^{ti272z} larvae, we estimated from the area of intersection the fraction of all DS voxels that are located

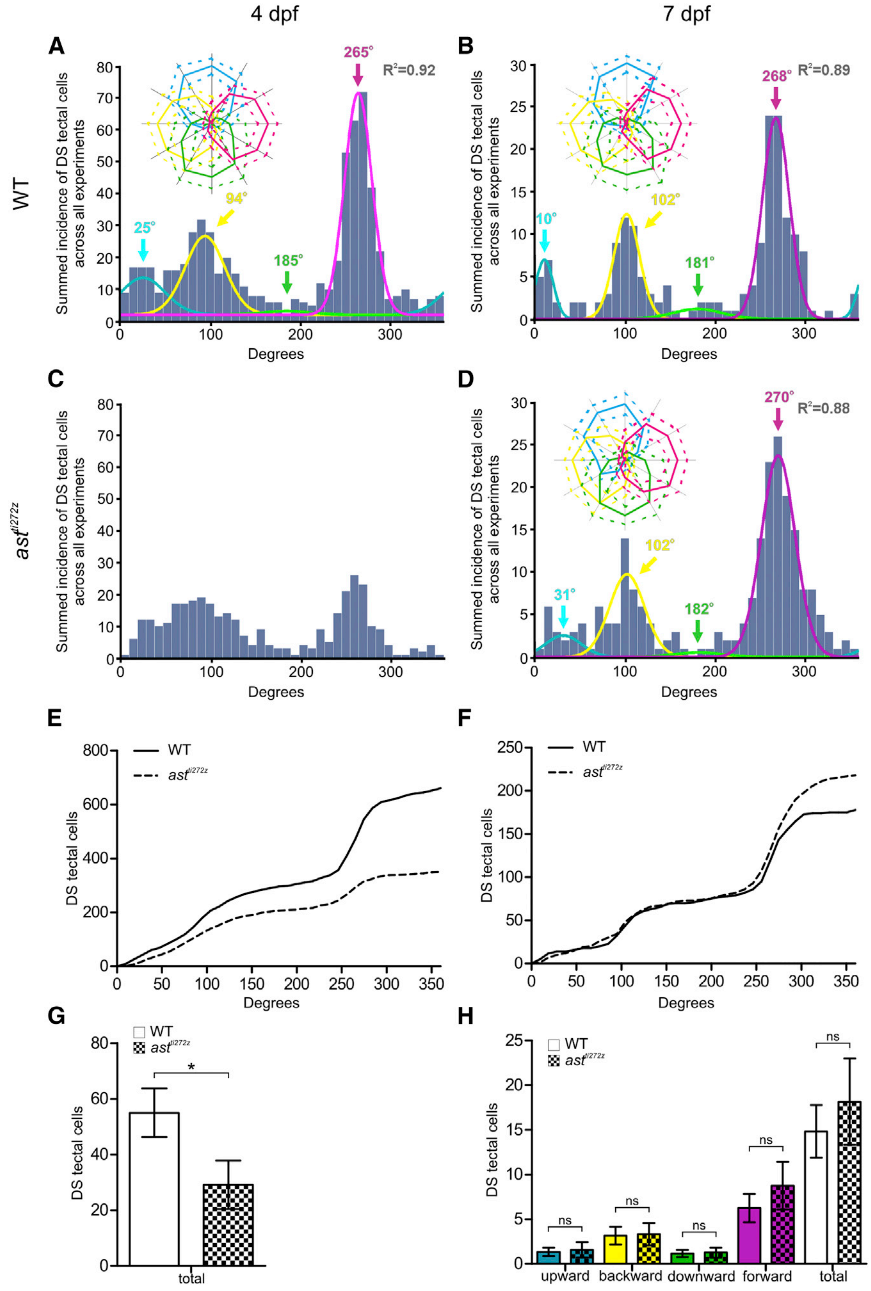

Figure 3. Functional Development of DS Tectal Cells Is Delayed in *astray* Mutants

Functional imaging of tectal cell responses in WT (n = 12, total of 36 optical sections) and *ast^{ti272z}* (n = 12, total of 36 optical sections) larvae with pan-neuronal expression of GCaMP5G (*Tg(elavl3:GCaMP5G)*).

(A–D) Cumulative histograms summarizing the incidence of preferred angles for DS tectal cells in WT larvae at 4 dpf (A) and 7 dpf (B), and *ast^{ti272z}* larvae at 4 dpf (C) and 7 dpf (D). Overlaid colored curves in (A), (B), and (D) show fitted von-Mises distributions used to identify the four DS subtypes and the color-coded arrows represent peak preferred angles. R^2 values relate to the summed von-Mises distributions. Insets in (A), (B), and (D) show polar plots of the mean (solid line) ± 1 SD (dashed line) normalized responses of cells within each DS subtype. Note that the same functional subtypes are present at 4 and 7 dpf in WT tecta.

(E and F) Cumulative plots generated from the data in (A)–(D) comparing the incidence of preferred angles for DS tectal cells between WT and *ast^{ti272z}* larvae at 4 dpf (E) and 7 dpf (F).

(G and H) Quantification of DS tectal cell number at 4 dpf (G) and 7 dpf (H). Note the significant reduction in DS tectal cell numbers at 4 dpf, but recovery at 7 dpf. All graphs show mean values ± SEM. *p < 0.05; ns, not significant, unpaired t test. See also Figures S4 and S5, Table S2, Movies S3 and S4.

in the correct location in mutant tecta. At 4 dpf, this fraction is 24% (upward motion), 43% (downward motion), and 38% (forward motion). At 7 dpf, the fraction is 44% (upward motion), 54% (downward motion), and 46% (forward motion) (Figures S3A–S3F). These data show that while the functional properties of DS-RGCs targeting the tectum are normal in *ast^{ti272z}* mutants, the laminar architecture of these inputs is absent at both 4 and 7 dpf.

Development of DS Tectal Cell Responses Is Delayed in *astray* Mutants

Our next goal was to determine how, if at all, spatial disorder in the retinal inputs to the tectum impacts the functional development of DS tectal neurons. We accomplished this by performing functional imaging of tectal neurons in *ast^{ti272z}* mutants expressing GCaMP5G in the majority of neurons in the brain *(Tg(elavl3:GCaMP5G)* (Ahrens et al., 2013) (Movies S3 and S4). DS-tectal neurons were identified by functional imaging at 4 dpf and 7 dpf using the procedure for RGCs described above (Figures S4A–S4F). DS metrics (DSI > 0.5, OSI < 0.5 and R^2 > 0.8) were applied to all visually responsive voxels followed by automatic aggregation of similarly tuned voxels into cell body-sized units (see Experimental Procedures). The resultant preferred angles of all DS tectal cells were summed across all subjects within each of the four experimental groups (WT and *ast^{ti272z}* larvae imaged at 4 and 7 dpf). Surprisingly, cumulative histograms reveal almost identical distributions in the preferred angles of tectal cells in WT and *ast^{ti272z}* larvae at 7 dpf (Figures 3B, 3D, and 3F). These distributions were fit by four summed von-Mises distributions (overlaid color curves in Figures 3B and 3D), demonstrating that the four DS subpopulations of tectal cells identified previously (tuned to upward, backward, downward, and forward motion) (Hunter et al., 2013) are present in both WT and *ast^{ti272z}* tecta. Furthermore, we found no significant difference in the number of DS tectal cells in each subtype or in

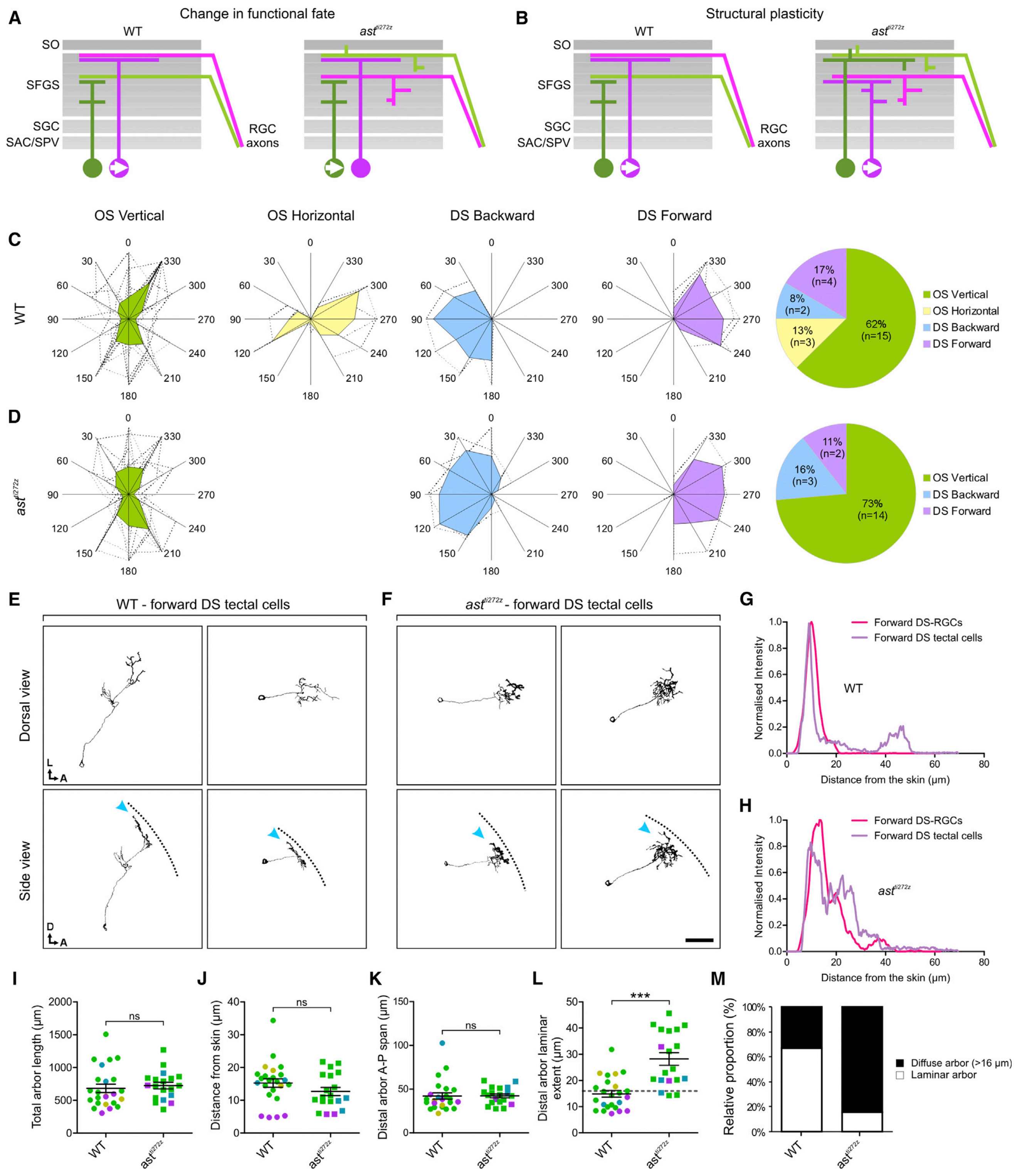

Figure 4. Morphology of DS Tectal Cell Dendrites Is Disrupted in the *astray* Mutant

(A and B) Two alternative models of how direction selectivity recovers in the tectal cell population in *ast*^{ti272z} mutants, functional plasticity (A) or structural plasticity (B). SO, stratum opticum; SFGS, stratum fibrosum et griseum superficiale; SGC, stratum griseum centrale; SAC, stratum album centrale; SPV, stratum periventriculare.

(legend continued on next page)

the total number of DS tectal neurons between WT and ast^{ti272z} larvae (Figure 3H). (The decrease in DS-tectal neurons seen in WT larvae reflects the progressive loss of GCaMP5G expression rather than a real reduction in DS tectal cell numbers, which have been shown to be constant during development [Niell and Smith, 2005].) We also found that tuning angle and bandwidths of DS tectal neurons were not significantly altered in mutant larvae (insets in Figures 3B and 3D; Table S2). These results demonstrate that despite the loss of laminated retinal input, all DS tectal neuron subtypes, including the emergent population tuned to backward motion, are established in appropriate numbers by 7 dpf. However, functional imaging of the same animals at 4 dpf reveals a marked reduction in the numbers of DS tectal cells across all preferred angles (Figures 3A, 3C, and 3E), resulting in a significant reduction in the total number of DS tectal neurons in the tectum of ast^{ti272z} mutants at 4 dpf (Figure 3G; WT: 55 ± 8.7, ast^{ti272z}: 29.1 ± 8.6, $p < 0.05$). (The four normally distributed populations are not as clearly evident in the cumulative data from 4 dpf ast^{ti272z} mutants, precluding the possibility of identifying the four functional subtypes at this stage.) The reduction in DS tectal cell number is not due to delayed proliferation or differentiation, because the numbers of mitotic (PH-3^{+}) and postmitotic (HuC/D^{+}) cells in the developing ast^{ti272z} tectum at 48 hpf are comparable to those found in WT animals (Figures S5A–S5D). The reduced number of DS tectal neurons is also unlikely to result from a failure to form retinotectal synapses per se, since the number of presynaptic sites in RGC axons is actually increased in ast^{ti272z} mutants (Campbell et al., 2007). These results show that loss of a laminar architecture results in a significant delay in the functional development of DS circuits in ast^{ti272z} mutant tecta.

Lamination of DS-Tectal Cell Dendrites Is Lost in *astray* Mutants

The recovery of tectal direction selectivity at 7 dpf in ast^{ti272z} mutants demonstrates a striking ability of the tectum to adapt to the loss of laminated retinal input. We reasoned that two mechanisms could account for functional recovery: (1) a change in functional fate, in which tectal cells that are not fated to become DS maintain their morphology in the mutant tectum but form synapses indiscriminately with the misplaced DS-RGC axons and, in so doing, adopt directional tuning (Figure 4A). Such a scenario would imply that lamination cues such as Robo2 are necessary for wiring specificity. (2) Structural plasticity, in which DS-tectal cells adjust their dendritic morphology in order to locate and synapse with the misplaced DS-RGCs. This would imply that cell-type-specific wiring is retained in the mutant and that structural plasticity in the developing brain enables functional and cell-type-specific connections to form despite the loss of lamination cues (Figure 4B). To distinguish between these mechanisms, it was necessary to label tectal cells whose morphology and function is predictable so that we could determine which is changing in the mutant. Here we identify the *FoxP2.A* enhancer as a means to genetically target subtypes of tectal cells (Bonkowsky et al., 2008). We used the *FoxP2.A* enhancer to drive expression of Gal4 and co-electroporated *FoxP2.A:Gal4FF* and *5UAS:GCaMP6F* plasmids into the tectum to label isolated tectal cells with the genetically encoded reporter of neural activity, GCaMP6F (Chen et al., 2013). This allowed us to examine both the tuning and morphology of single tectal cells in the WT and mutant tectum at 7 dpf (an example of a tuning experiment in single cell is shown in Movie S5). We found that tectal cells labeled by this approach could be grouped into four functional subtypes: 62% of labeled cells were selective for horizontally oriented bars moving along the vertical axis (vertically tuned), 13% were selective for vertically oriented bars moving along the horizontal axis (horizontally tuned), 8% were backward DS, and 17% were forward DS (Figure 4C; also Figures S6A–S6F for response profiles of individual FoxP2.A-labeled cells). Morphological reconstruction of these cell types revealed that they are morphologically diverse (Figures S7B and S7C) but, consistent with a previous study (Gabriel et al., 2012), we find that WT DS tectal cells tuned to forward motion have laminar dendrites (arrowheads in Figure 4E; Movie S6) that co-stratify with the axons of DS-RGCs in superficial sublaminae within SFGS (Figure 4G). Three of these subtypes were also labeled by the *FoxP2.A:Gal4FF* construct in the tectum of ast^{ti272z} mutants and in similar proportions to that seen in WT tecta (Figure 4D; also Figures S6G–S6L for response profiles of individual FoxP2.A-labeled cells). The exception was an absence of horizontally tuned cells—one of the cell types rarely labeled in WT larvae. The fact that the *FoxP2.A* enhancer labels similar functional subtypes of tectal neuron in the WT and mutant tectum suggests that a change in functional fate of tectal neurons is unlikely to account for the recovery of tectal direction selectivity in ast^{ti272z} mutants. If this were the case, we would expect to see a change either in tuning

(C and D) Polar plots illustrating functional subtypes of WT (n = 24 in 22 larvae) (C) and ast^{ti272z} (n = 19 in 16 larvae) (D) tectal cells singly labeled with GCaMP6F in the 7 dpf tectum following co-electroporations of *FoxP2.A:Gal4FF* + *5UAS:GCaMP6F* DNA constructs. Dashed lines indicate polar plots from individual cells, and filled color-coded polar plots represent the mean for each functional subtype. Pie charts to the right indicate the relative proportions of functional subtypes and number of neurons analyzed.

(E and F) Morphologies of tectal cells selective for forward motion. Two examples from WT (E) and ast^{ti272z} tecta (F) are shown. Note that the laminar profile of DS tectal cells tuned to forward motion is lost in ast^{ti272z} animals (arrowheads). Dashed lines indicate the position of the skin overlaying the tectum. Scale bar represents 30 μm. A, anterior; D, dorsal; L, lateral.

(G and H) Line plots comparing the distribution of forward tuned DS-RGC axons and forward tuned DS tectal cell dendrites along the laminar axis of the tectum at 7 dpf in WT (G) and ast^{ti272z} (H) animals. Line plots show normalized intensity of each cell type as a function of its distance from the skin. Note the alignment of DS-RGC axons and DS tectal dendrites in both conditions.

(I–L) Comparison of four morphological parameters extracted from all tectal cells (Figure S4A). For total arbor length (I), 682.6 ± 62.4 for WT and 724.6 ± 50.5 for ast^{ti272z}; for distance from skin (J), 15.2 ± 1.3 for WT and 12.6 ± 1.2 for ast^{ti272z}; for distal arbor anterior-posterior span (K), 42 ± 3.6 for WT and 42.3 ± 2.2 for ast^{ti272z}; and for distal arbor laminar extent (L), 14.9 ± 1.2 for WT and 28.2 ± 2.4 for ast^{ti272z}. Data points are color coded according to functional subtype as in (C) and (D). All graphs show mean values ± SEM. ***$p < 0.001$; ns, not significant, Mann Whitney test.

(M) Proportions of cells with laminar and diffuse (distal arbor laminar extent < 16 μm and > 16 μm, respectively) arbors in WT and ast^{ti272z} tecta. WT: 67% laminar, 33% diffuse arbor; and ast^{ti272z}: 16% laminar, 84% diffuse arbor. See also Figures S6 and S7 and Movies S5 and S6.

or in the proportions of functional subtypes targeted by the *FoxP2.A* enhancer. To examine whether structural plasticity could account for the functional recovery of direction selectivity in the tectum, we reconstructed the morphology of tectal neurons labeled by the *FoxP2.A* enhancer in ast^{ti272z} mutants. We found that dendrites from forward DS tectal cells in the ast^{ti272z} tectum lose their characteristic laminar profile and are instead diffuse (arrowheads in Figure 4F; Movie S6). However, by overlaying line plots showing the distribution of forward tuned DS tectal dendrites and RGCs in the mutant, we show that the two profiles nevertheless still show considerable overlap (Figure 4H). To quantify the change in morphology of tectal dendrites, we measured four morphological features of all imaged tectal cells in WT and ast^{ti272z} tecta (Figure S7A). Total arbor length, distance of distal arbor from skin, and the anterior-posterior span of the distal arbor are not significantly different in mutant and WT larvae (Figures 4I–4K). However, dendrites of tectal cells have a significantly broader laminar profile in ast^{ti272z} mutants (Figure 4L; WT: 14.9 ± 1.2, ast^{ti272z}: 28.2 ± 2.4, $p < 0.001$), and a higher proportion of them possess diffuse dendritic arbors (>16 μm extent in laminar axis) (Figure 4M; WT: 33%, ast^{ti272z}: 84%). Thus, dendritic lamination is lost but tuning properties of tectal cells targeted by the *FoxP2.A:Gal4FF* are unaltered in the mutant. These data suggest a model of functional recovery in which structural plasticity in the mutant tectum establishes functional connections between DS-RGCs and their normal tectal targets. It is interesting to note that tectal cells tuned to posterior motion and some vertically tuned tectal neurons also have laminar dendrites and that this lamination appears to be lost in the mutant tectum. This suggests that structural plasticity perhaps ensures appropriate functional wiring of multiple tectal neuron subtypes when lamination cues are lost (Figure 4L).

Robo2 Guides Laminar Growth of Tectal Cell Dendrites

Two mechanisms may potentially account for the switch from a laminar to a diffuse dendritic morphology of tectal cells in the mutant tectum. RGC axons may simply act as a scaffold for tectal dendrites—if DS-RGC axons are diffusely distributed in the mutant then so are the dendrites of tectal neurons. Alternatively, Robo2 may play a direct role in guiding laminar growth of tectal cell dendrites. In addition to the expression in RGCs, Robo2 is also expressed in tectal neurons but its role here is unknown (Campbell et al., 2007). To address this, we enucleated WT and mutant zebrafish prior to RGC innervation of the tectum in order to remove any positional information that might be provided to tectal dendrites by RGC axons. We then mosaically labeled tectal cells by co-electroporating *FoxP2.A:Gal4FF* and *5UAS:tdTomato* DNA constructs into tecta deprived of retinal input. Labeled cells were imaged at 7 dpf and morphologically reconstructed (Figure 5A). Total dendritic arbor length and anterior-posterior span were unaltered by enucleation in either WT or ast^{ti272z} larvae (Figures 5B and 5D). However, while tectal cells in WT enucleated animals retain their laminar morphology (Figure 5E, laminar extent of dendrites = 11.8 μm ± 0.9; Figure 5F, proportion of laminar arbors = 86%), lamination of tectal dendrites is lost in enucleated ast^{ti272z} larvae (Figures 5E, laminar extent = 22.4 μm ± 2.9; Figure 5F, proportion of laminar arbors = 20%). Thus, even in the absence of disorganized RGC axons, tectal dendrites lose dendritic stratification in the ast^{ti272z} tectum. These findings suggest that Robo2 can direct laminar growth of DS tectal cell dendrites.

Rapid Development of Tectal Direction Selectivity Requires Robo2 in RGC Axons and Tectum

Our data show that Robo2 can drive lamination of both tectal dendrites and RGC axons. Thus, by bringing axons and dendrites into precise spatial alignment Robo2 may speed the functional development of retinotectal connections. In this scenario, Robo2 expression would be required in both RGCs and tectal neurons. To test this, we transplanted mutant eyes into WT hosts and vice-versa prior to retinal differentiation and RGC innervation of the tectum. The WT and ast^{ti272z} hosts had pan neuronal expression of GCaMP5G (*Tg(elavl3:GCaMP5G)*), allowing us to perform functional imaging of the tectal population receiving input from the transplanted eye at 4 dpf and then again at 7 dpf (Figure 6A). To confirm the success of each transplantation experiment, we fixed samples post-functional imaging at 7 dpf and we bulk-loaded the donor and host eyes with the lipophilic dyes, DiI and DiD, respectively, in order to label the entire retinotectal projection (Figures S8A–S8D). As an additional control, we transplanted either mutant or WT eyes expressing SyGCaMP3 and then performed functional imaging of RGC axons originating from the transplanted eye (Figure S8E). This showed that regardless of the host-donor combination, similar numbers of DS-RGC voxels were found in the host tectum, indicating that functional development of DS-RGCs and innervation of the tectum is unaffected by the transplantation procedure or the differing genotypes of host and donor (Figures S8F and S8G). Each transplantation experiment fell within one of the following three groups: WT-to-WT group, which serves as a control group, and two experimental groups WT-to-ast^{ti272z} and ast^{ti272z}-to-WT transplants. Functional imaging of the population of tectal cells revealed that WT-to-ast^{ti272z} and ast^{ti272z}-to-WT groups have, on average, significantly less DS tectal cells compared to the WT-to-WT group at 4 dpf (Figure 6B; WT-to-WT: 53.4 ± 11.5, WT-to-ast^{ti272z}: 21.9 ± 7.9, and ast^{ti272z}-to-WT: 15.8 ± 6.1, $p < 0.05$). These findings indicate that Robo2 expression is required in both RGCs and tectal neurons for the rapid (by 4 dpf) development of DS retinotectal connections. However, at 7 dpf, the number of DS tectal cells in the two experimental groups match those found in the control, WT-to-WT group (Figure 6C), suggesting that DS tectal responses eventually recover in tecta receiving input from transplanted eyes.

Structural Plasticity of RGC Axons or Tectal Neurons Compensates for Loss of Robo2

The recovery of tectal direction selectivity in the transplants is surprising because of the molecular mismatch between tectal cells and RGC axons—the Robo2-positive cell type has a lamination cue while the Robo2-negative neuron does not. How then do the two mismatched cell types eventually connect with one another? To address this question, we transplanted WT eyes expressing SyGCaMP3 in RGCs into ast^{ti272z} hosts (Figure 8A). By performing functional imaging and generating composite functional maps, we show that the WT transplanted DS-RGCs show their normal sublaminar distribution within

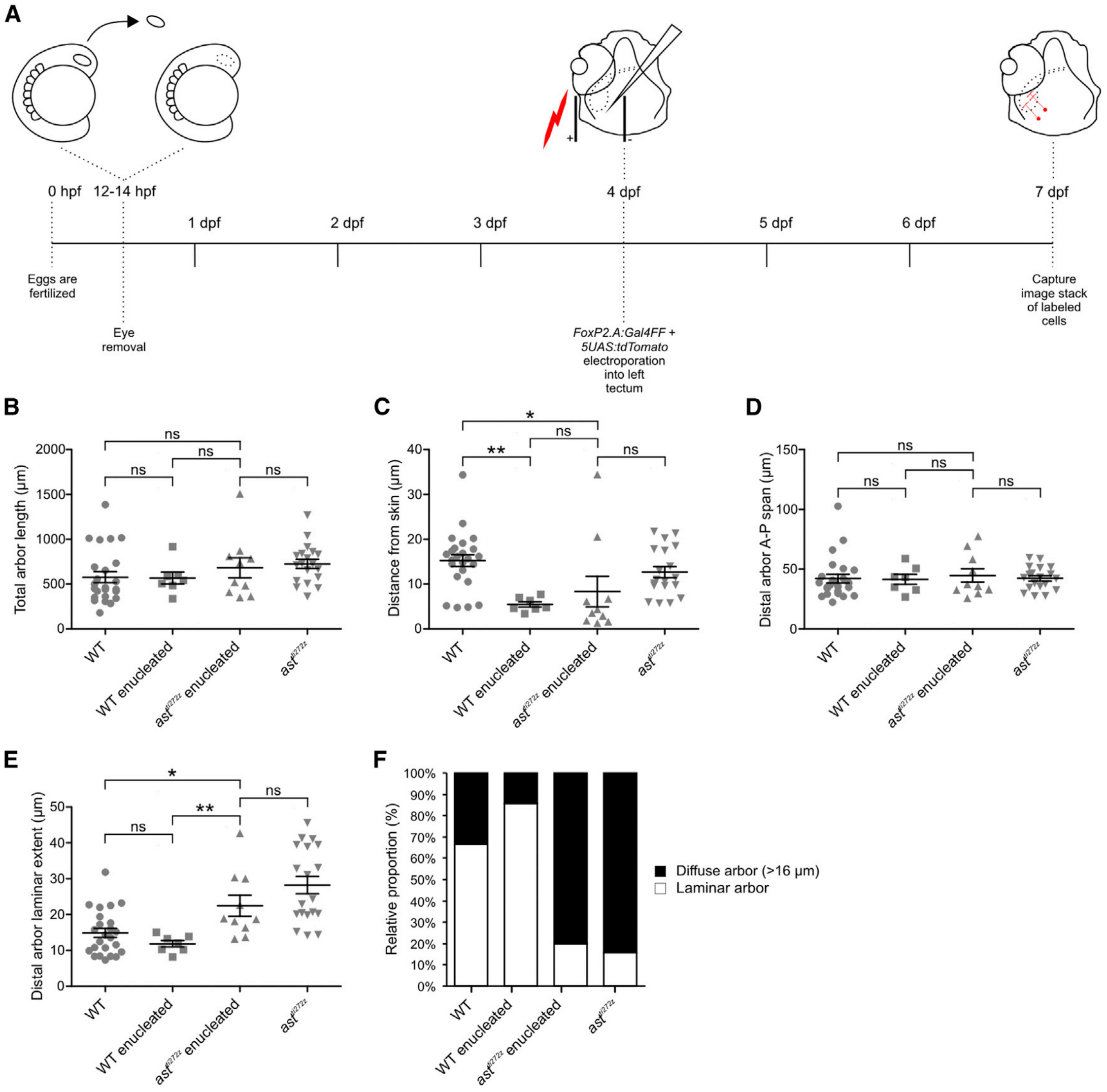

Figure 5. Robo2 Directs Laminar Growth of Tectal Cell Dendrites

(A) Experimental procedure used to assess the role of RGC axons and/or Robo2 in regulating the morphology of tectal cell dendrites. Optic vesicles were removed at 12–14 hpf. Single cells were labeled by co-electroporating *FoxP2.A:Gal4FF + 5UAS:tdTomato* DNA constructs into the deprived tectum at 4 dpf. Single tectal cells were imaged at 7 dpf.

(B–E) Comparison of morphological parameters of tectal cells in four conditions: WT (n = 24 cells in 22 larvae), WT enucleated (n = 7 cells in 6 larvae), ast^{ti272z} enucleated (n = 10 cell in 9 larvae), and ast^{ti272z} (n = 19 cells in 16 larvae). For total arbor length (B), 682.6 ± 62.4 for WT, 567.4 ± 66.7 for WT enucleated, 681.7 ± 110.4 for ast^{ti272z} enucleated, and 724.6 ± 50.5 for ast^{ti272z}; for distance from skin (C), 15.2 ± 1.3 for WT, 5.4 ± 0.5 for WT enucleated, 8.3 ± 3.4 for ast^{ti272z} enucleated, and 12.6 ± 1.2 for ast^{ti272z}; for distal arbor anterior-posterior span (D), 42 ± 3.6 for WT, 41.4 ± 4.1 for WT enucleated, 44.6 ± 5.6 for ast^{ti272z} enucleated, and 42.3 ± 2.2 for ast^{ti272z}; and for distal arbor laminar extent (E), 14.9 ± 1.2 for WT, 11.8 ± 0.9 for WT enucleated, 22.4 ± 2.9 for ast^{ti272z} enucleated, and 28.2 ± 2.4 for ast^{ti272z}. All graphs show mean values ± SEM. $^{**}p < 0.01$; $^{*}p < 0.05$; ns, not significant, Kruskal-Wallis and Dunn's multiple comparison tests.

(F) Proportions of cells with laminar and diffuse (distal arbor laminar extent < 16 μm and > 16 μm, respectively) arbors within each experimental group. WT: 67% laminar, 33% diffuse arbor; WT enucleated: 86% laminar, 14% diffuse arbor; ast^{ti272z} enucleated: 20% laminar, 80% diffuse arbor; and ast^{ti272z}: 16% laminar, 84% diffuse arbor.

SFGS, consistent with a role for Robo2 in laminar targeting of RGC axons (Figures 8B, 8C, and 8E). Furthermore, this suggests that functional recovery in the WT-to- ast^{ti272z} transplants is not due WT RGCs shifting their position within the mutant tectum in order to locate the misplaced tectal dendrites. Functional recovery must therefore result from structural plasticity of tectal neurons in the ast^{ti272z} tectum. To test this, we mosaically labeled tectal cells by co-electroporating *FoxP2.A:Gal4FF* and *5UAS: tdTomato* DNA constructs into the tectum receiving input from the WT transplanted eye. Labeled cells were imaged at 7 dpf and morphologically reconstructed (Figure 7A). Strikingly, when Robo2-negative tectal neurons receive input from WT

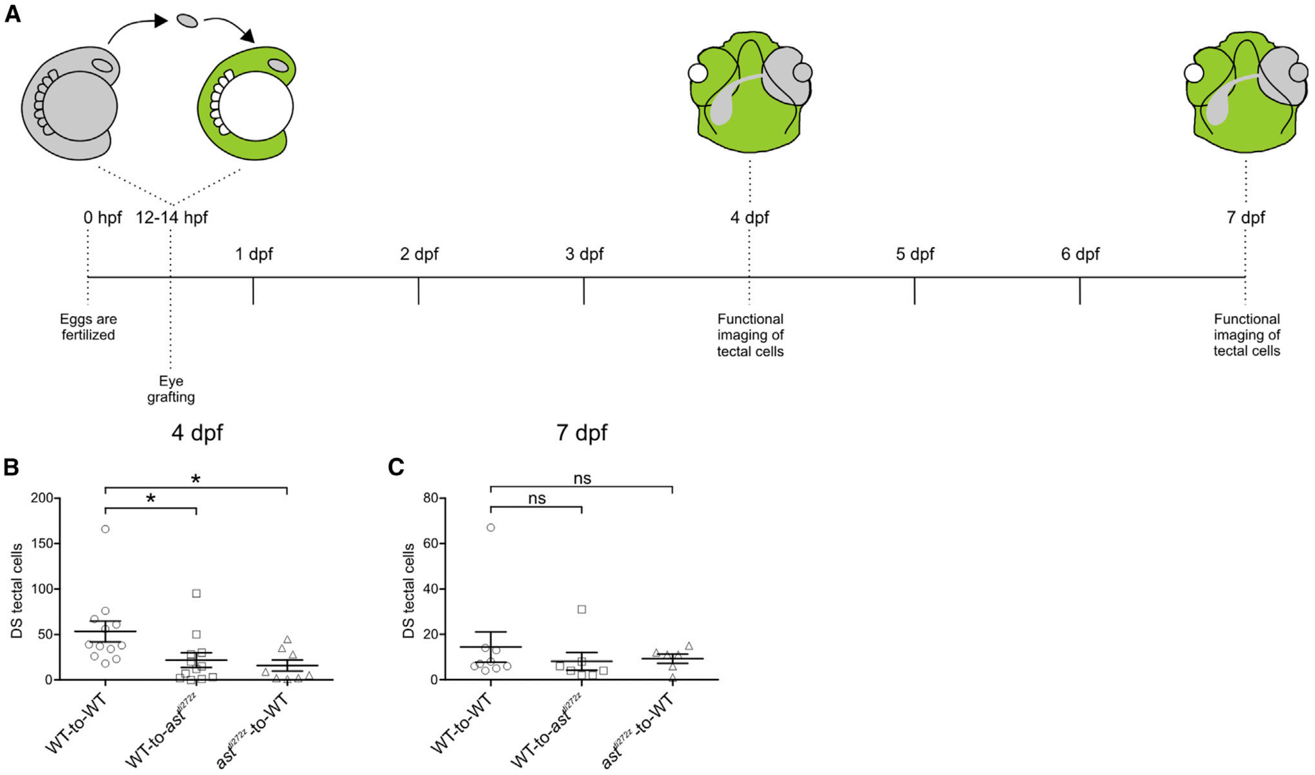

Figure 6. Robo2 Is Required in RGC Axons and Tectum for Rapid Development of DS Responses in Tectal Cells

(A) Experimental procedure used to assess the cellular requirements for Robo2 in the functional development of DS tectal cells. Optic vesicles from donor embryos were transplanted into *Tg(elavl3:GCaMP5G)* hosts at 12-14 hpf and functional imaging of tectal cell responses was performed at 4 and 7 dpf.

(B and C) Quantification of average number of DS tectal cells per group. Total number of DS cells at 4 dpf (B), 53.4 ± 11.5 for WT-to-WT (n = 12 larvae, total of 36 optical sections), 21.9 ± 7.9 for WT-to-ast^{ti272z} (n = 12 larvae, total of 36 optical sections), and 15.8 ± 6.1 for ast^{ti272z}-to-WT (n = 8 larvae, total of 24 optical sections). Total number of DS cells at 7 dpf (C), 14.4 ± 6.6 for WT-to-WT (n = 9 larvae, total of 27 optical sections), 8.1 ± 3.8 for WT-to-ast^{ti272z} (n = 7 larvae, total of 21 optical sections), and 9.3 ± 2 for ast^{ti272z}-to-WT (n = 6 larvae, total of 18 optical sections). All graphs show mean values ± SEM. *p < 0.05; ns, not significant, Kruskal-Wallis and Dunn's multiple comparison tests. See also Figure S8.

RGCs, the proportion of cells that are laminar is identical to that seen in WT animals and in the WT-to-WT transplant controls (Figures 7E and 7F). Furthermore, dendrites of Robo2-negative tectal neurons that receive input from WT RGCs are morphologically indistinguishable from cells labeled in the WT tectum (Figures 7B–7D). These findings suggest that WT RGC axons can provide lamination cues for Robo2-negative tectal neurons, which themselves exhibit sufficient structural plasticity to target the correct lamina despite the loss of lamination cues provided by Robo2. We then performed the converse experiment by transplanting ast^{ti272z} eyes expressing SyGCaMP3 in RGCs into WT hosts (ast^{ti272z}-to-WT group). We show that Robo2-positive tectal cells receiving input from Robo2-negative RGC axons have laminar dendrites (Figures 7E and 7F). This is consistent with a role for Robo2 in guiding growth of tectal dendrites and demonstrates that WT tectal neurons do not alter their dendritic morphology when receiving input from mutant RGC axons. Thus, in this scenario functional recovery of tectal direction selectivity must result from the shifting of DS-RGC axons to the correct lamina. To test this, we performed functional imaging of mutant DS-RGC axons in the WT tectum and generated composite functional maps at 7 dpf. Because of the technically challenging nature of the experiments, the numbers of animals imaged in this way were relatively low (n = 4). This combined with the fact that DS-RGCs tuned to upward and downward motion are rare means that composite maps generated for these subtypes appear patchy. It is therefore difficult to reliably quantify the distributions of these subtypes in the ast^{ti272z}-to-WT group. For this reason, we focused on the largest subpopulation of DS-RGC tuned to forward motion. We find that the distribution of mutant DS-RGCs in WT hosts is significantly more laminar than that seen in ast^{ti272z} animals (compare Figure 8D with Figure 2D). Furthermore, the fraction of correctly positioned DS-RGC voxels is virtually normal when ast^{ti272z} RGC axons are within a WT tectum (Figures 8E and 8F). Thus, the presence of WT tectal dendrites restores the normal laminar functional architecture in Robo2-negative DS-RGCs. These findings also suggest that structural plasticity of Robo2-negative RGC axons enables them to locate their Robo2-positive tectal targets. Collectively, the above findings imply that (1) tectal cells and RGC axons can provide positional information to one another, and (2) structural plasticity in RGC axons or tectal dendrites can compensate for the lack of lamination cues provided by Robo2.

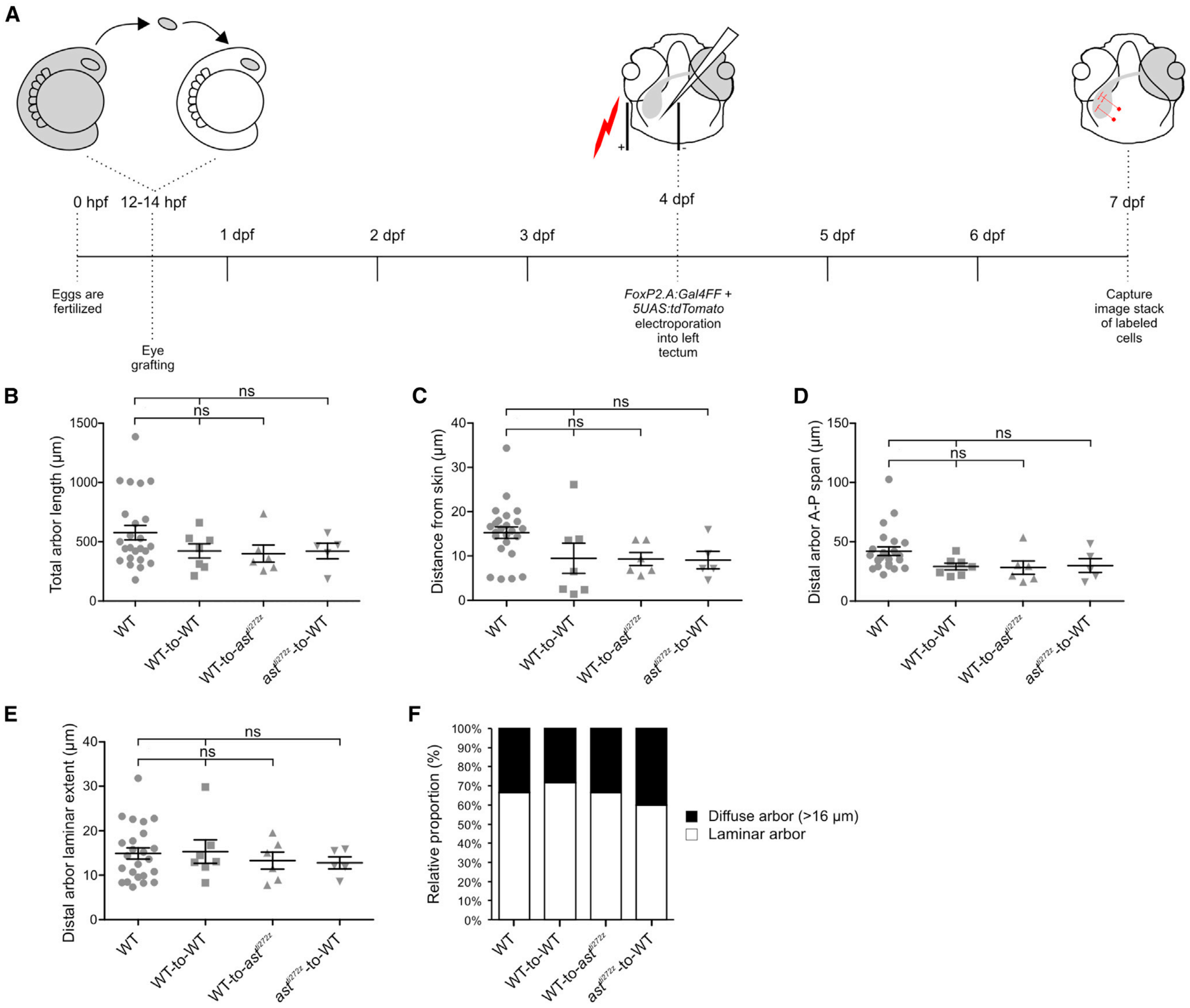

Figure 7. *robo2*$^{+/+}$ RGC Axons Provide Positional Cues for *robo2*$^{-/-}$ Tectal Cell Dendrites

(A) Schematic showing the experimental procedure followed. Optic vesicles from donor embryos were transplanted into hosts at 12–14 hpf. Single cells were labeled by co-electroporating *FoxP2.A:Gal4FF* + *5UAS:tdTomato* DNA constructs into the tectum receiving retinal input from transplanted eyes at 4 dpf and imaged at 7 dpf.

(B–E) Comparisons of the main morphological parameters of tectal cells in four conditions: WT (n = 24 in 22 larvae), WT-to-WT (n = 7 in 6 larvae), WT-to-*ast*^{ti272z} (n = 6 in 5 larvae), and *ast*^{ti272z}-to-WT (n = 5 in 4 larvae) tectal cells. For total arbor length (B), 682.6 ± 62.4 for WT, 423.6 ± 60 for WT-to-WT, 399.5 ± 71.9 for WT-to-*ast*^{ti272z}, and 421.8 ± 64.5 for *ast*^{ti272z}-to-WT; for distance from skin (C), 15.2 ± 1.3 for WT, 9.4 ± 3.4 for WT-to-WT, 9.3 ± 1.4 for WT-to-*ast*^{ti272z}, and 9 ± 1.9 for *ast*^{ti272z}-to-WT; for distal arbor anterior-posterior span (D), 42 ± 3.6 for WT, 29.2 ± 2.9 for WT-to-WT, 28.2 ± 5.5 for WT-to-*ast*^{ti272z}, and 29.9 ± 5.8 for *ast*^{ti272z}-to-WT; and for distal arbor laminar extent (E), 14.9 ± 1.2 for WT, 15.3 ± 2.6 for WT-to-WT, 13.2 ± 1.8 for WT-to-*ast*^{ti272z}, and 12.8 ± 1.3 for *ast*^{ti272z}-to-WT. All graphs show mean values ± SEM ns, not significant, Kruskal-Wallis and Dunn's multiple comparison tests.

(F) Proportions of cells with laminar and diffuse (distal arbor laminar extent < 16 μm and > 16 μm, respectively) arbors within each experimental group. WT: 67% laminar, 33% diffuse arbor; WT-to-WT: 71% laminar, 29% diffuse arbor; WT-to-*ast*^{ti272z}: 67% laminar, 33% diffuse arbor; and *ast*^{ti272z}-to-WT: 60% laminar, 40% diffuse arbor.

DISCUSSION

The aim of our study was to exploit the *astray* mutant zebrafish to investigate how loss of a layered neural organization impacts the functional development of neural circuits. We focused on the development of direction-selective circuits and found that while the laminar organization of DS retinal inputs to the tectum is lost in the *astray* tectum, the tuning properties of these inputs are normal. However, functionally intact DS input from the retina, by itself, is not sufficient to establish early development of DS circuits within the tectum. Thus, precise laminar targeting of retinal inputs to the tectum is required for rapid assembly of

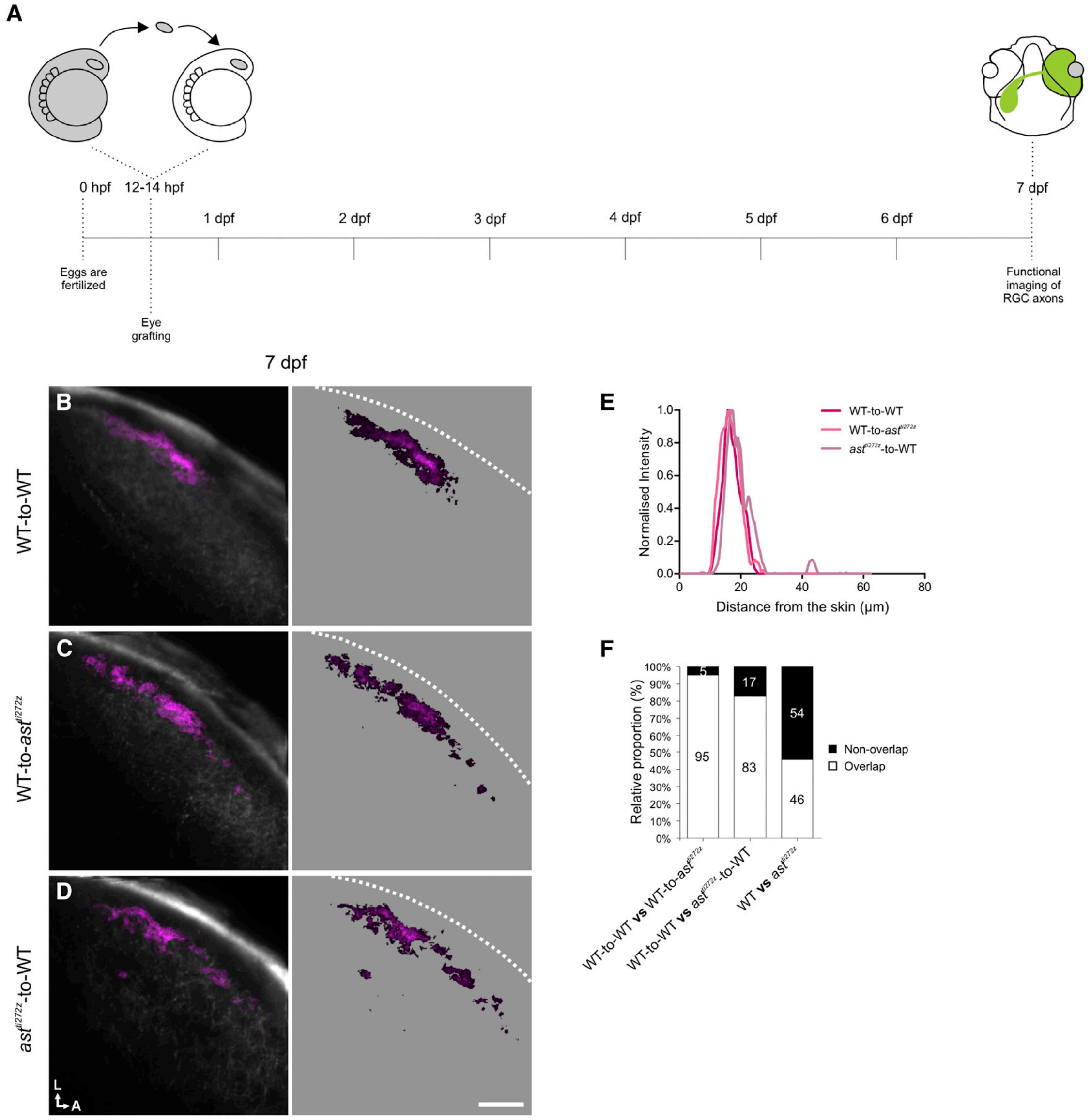

Figure 8. *robo2*$^{+/+}$ Tectal Cell Dendrites Provide Positional Cues for *robo2*$^{-/-}$ RGC Axons

(A) Schematic showing the experimental procedure followed. Optic vesicles from *Tg(Isl2b:Gal4;UAS:SyGCaMP3)* donor embryos were transplanted into hosts at 12–14 hpf and functional imaging of donor RGC axons within the tectum was performed at 7 dpf.

(B–D) Composite parametric maps across all fish imaged showing the spatial distribution of forward tuned DS voxels in the tectal neuropil of WT-to-WT (n = 8 larvae, total of 24 optical sections) (B), WT-to-*ast*^{ti272z} (n = 4 larvae, total of 12 optical sections) (C), and *ast*^{ti272z}-to-WT (n = 4 larvae, total of 12 optical sections) (D). Within individual parametric maps, voxel brightness is proportional to the summed incidence of the functional subtype across all larvae imaged. The standard space template image derived from the mean fluorescence image of SyGCaMP3-expressing axons (grayscale) provides an anatomical reference. Dashed lines indicate the position of the skin overlaying the tectum. Scale bar represents 20 µm. A, anterior; L, lateral.

(E) Line plots generated from the composite parametric maps in (B)–(D) illustrating the laminar organization of forward tuned DS voxels within each group. Line plots show normalized intensity of DS voxels as a function of distance from the skin.

(F) Pairwise comparisons showing the extent of spatial overlap in the distribution of forward tuned DS voxels in the different experimental groups. Note the increased fraction of correctly positioned DS voxels in transplanted *ast*^{ti272z} axons (*ast*^{ti272z}-to-WT group) compared with the *ast*^{ti272z} condition.

direction-selective circuits. A surprising finding from our study is that directional tuning in individual neurons and population encoding of directional motion by tectal neurons at later stages of development is indistinguishable from that seen in WT larvae. These findings echo those from the *reeler* mouse mutant that found that visual and somatosensory areas of the cortex were functionally intact despite the loss of a laminated architecture (Dräger, 1981; Guy et al., 2015; Lemmon and Pearlman, 1981; Simmons and Pearlman, 1983). Similarly mice with null mutations in *PlexinA4* or *Semaphorin6A* exhibit severe defects in

lamina-specific neurite arborization of tyrosine hydroxylase-expressing dopaminergic amacrine cells (TH-ACs) and their synaptic partners—the intrinsically photosensitive RGCs (ipRGCs) (Matsuoka et al., 2011). However, the axons of TH-ACs still overlap with the dendrites of ipRGCs in ectopic locations, suggesting that specific wiring between these cell types persists in these mutants (Matsuoka et al., 2011). Because these studies focused on a single time point late in development, a role for laminae in speeding circuit assembly would not have been apparent. The ability to follow the functional development of circuits within a single animal is a particular strength of the zebrafish and is one that has allowed us to provide experimental evidence demonstrating a role for layers in the brain. It is important to note that although tuning of individual neurons and population encoding of directional motion are ultimately intact in the *astray* tectum, other aspects of circuit function, which cannot be measured using functional calcium imaging, may not. There is increasing evidence that sophisticated local processing may be carried out within the dendritic tree, with nonlinear interactions between closely spaced synaptic inputs shaping the output of the neuron (Branco and Häusser, 2010). The loss of a laminar dendritic profile in tectal dendrites in the *astray* tectum that we and others have observed (Xiao et al., 2011) is likely to significantly alter the spatial relationship between synaptic inputs and consequently alter how these inputs are integrated in tectal neurons. Indeed, the optic tectum of *astray* mutants may be a useful model for understanding how changes in dendritic geometry alter the integrative properties of neurons.

Our study also provides new information on the molecular mechanisms that guide laminar-specific targeting of neurites and in so doing provides insight into precisely why lamination speeds circuit assembly. We show that in addition to targeting of DS-RGC axons, Robo2 guides laminar targeting of DS-tectal cell dendrites. This finding supports a simple model for how Slit-Robo signaling brings pre- and postsynaptic partners into rapid and precise alignment in the nascent tectal neuropil: a gradient of the Robo2 ligand, Slit1a, across the laminar axis of the tectum could provide positional information to growing RGC axons and tectal dendrites. Appropriately, matched axons and dendrites may interpret this gradient similarly, perhaps by expressing similar levels of the Robo2 receptor, thereby bringing cognate pre- and postsynaptic partners into spatial proximity. In this model, lamination cues such as Robo2 could speed circuit assembly simply by increasing the likelihood of contact between matching pre- and postsynaptic neurons. Support for such a model comes from our finding that Robo2 is required in both RGCs and tectal neurons for the rapid assembly of DS circuits in the tectum. A role for Slit-Robo signaling in coordinating the spatial arrangement of both pre- and postsynaptic elements has also been demonstrated in the leg neuropil of *Drosophila*, suggesting that an evolutionarily conserved role for Slit-Robo signaling as a global neuropilar organizer (Brierley et al., 2009).

We have also provided insight into the plasticity mechanisms that allow functional circuits to establish themselves when lamination is perturbed. Our transplantation experiments, in which one synaptic partner expresses Robo2 while the other does not, demonstrates that structural plasticity of Robo2-negative RGC axons or tectal dendrites is sufficient to compensate for the loss of this lamination cue. Time-lapse imaging studies of tectal dendrite and RGC axon growth in the normal zebrafish tectum have shown that both are extremely dynamic processes (Kaethner and Stuermer, 1992, 1997; Meyer and Smith, 2006; Niell et al., 2004; Simpson et al., 2013). Thus, the structural plasticity that enables functional recovery in the *astray* tectum is likely to be part of a normal developmental program rather than a process triggered specifically by the loss of lamination cues. An obvious implication of these findings is that cell-type-specific wiring is ultimately determined by mechanisms unrelated to Robo2 and lamination. Studies of neurite patterning in the inner plexiform layer (IPL) of the retina have implicated homophilic and heterophilic cell-surface molecules on axons and dendrites in synaptic partner recognition. For example, members of the immunoglobulin-domain-containing superfamily (Sidekick 1 and 2, Dscam, DscamL, Cadherin 8 and 9) have been identified as being important elements of a cell-surface recognition code that ensures precise connectivity in the chick and mouse retina (Duan et al., 2014; Krishnaswamy et al., 2015; Yamagata and Sanes, 2008; Yamagata et al., 2002). The precise nature of the code in the tectum or superior colliculus is unknown. Although such code could match specific pre- and postsynaptic neurons, it could not provide an explicit mechanism for determining precisely where a given layer might form relative to other layers. A global neuropil organizer such as Robo2 would not only increase the speed and likelihood of contact between pre- and postsynaptic neurons expressing complementary cell-surface codes, it may also account for the stereotypic order of sublaminae found in the zebrafish tectum.

Our findings suggest that lamination is not ultimately required for the correct wiring of neural networks, but they do not rule out a role for lamination in generating wiring specificity in other species or areas of the brain. For example, in the mouse retina, perturbation of the type II Cadherins, *cdh8* and *cdh9*, disrupts laminar targeting of axons of bipolar cell types, BC2 and BC5, in the inner plexiform layer—a perturbation that results in defective functional connectivity with ON-OFF DS-RGCs (Duan et al., 2014). However, in this instance it is difficult to attribute the functional deficits to the lamination errors since Cadherins may also play a direct role in synapse formation. Furthermore, the role of layers may well be diverse. The principle driving force for a laminated architecture may even vary between subtypes of the same neuron type. For example, in zebrafish laminar targeting of all subtypes of RGC is precise from early developmental stages and functional imaging studies have shown that, in addition to directional tuning, selectivity for other visual features is established very soon after RGC axons first enter the tectum (Niell and Smith, 2005). For young larval zebrafish, which are crucially dependent on a functional visual system for survival, speed of circuit assembly may be the main evolutionary driver of a laminated architecture. However, studies of RGC subtypes innervating the mouse superior colliculus have revealed diverse and subtype-specific strategies for generating lamina-specific connections. For example, axons of the direction-selective J- and BD-RGC subtypes target definitive laminae within the superior colliculus early in postnatal development suggesting that, as

in zebrafish, precise laminar targeting of RGC axons may facilitate rapid functional assembly of direction-selective circuits (Kim et al., 2010). In contrast, lamination of the non-direction-selective W3- and OFF-αRGC axons emerges gradually from an initially diffuse pattern (Huberman et al., 2008; Kim et al., 2010). Do these different developmental patterns reflect subtype-specific differences in the requirement for laminae themselves? Further studies are required to examine the diversity of roles played by layers in the brain, but our study demonstrates that while lamination appears to be dispensable for wiring specificity in direction-selective circuits, it is essential for the rapid functional assembly of these networks.

EXPERIMENTAL PROCEDURES

Transgenic and Mutant Lines

Transgenic lines *Tg(Isl2b:Gal4)*zc60, *Tg(UAS:SyGCaMP3)kg1* and *Tg(elavl3:GCaMP5G)a4598* have been previously described (Ahrens et al., 2013; Ben Fredj et al., 2010; Nikolaou et al., 2012). The *ast*^{ti272z} mutant allele (Fricke et al., 2001) was kindly provided by Catherine Becker and Thomas Becker (University of Edinburgh).

Functional Imaging, Voxel-Wise Analysis and Generation of Functional Maps

Visual stimulation, voxel-wise analysis, and generation of functional maps were performed as previously described (Hunter et al., 2013; Lowe et al., 2013; Nikolaou et al., 2012). (See also Supplemental Experimental Procedures.)

DNA Electroporations

Tectal cells were mosaically labeled by co-electroporating the *FoxP2.A:Gal4FF* activator plasmid (FoxP2.A enhancer construct was a gift of Joshua Bonkowsky, University of Utah) together with GCaMP6F or tdTomato effector plasmids, as previously described (Hoegler and Horne, 2010). (See also Supplemental Experimental Procedures.)

Tissue Micro-manipulations

Enucleations and eye transplantations were performed at 6–10 somite stage (12–14 hpf). (See also Supplemental Experimental Procedures.)

SUPPLEMENTAL INFORMATION

Supplemental Information includes Supplemental Experimental Procedures, eight figures, two tables, and six movies and can be found with this article online at http://dx.doi.org/10.1016/j.neuron.2015.10.020.

A video abstract is available at http://dx.doi.org/10.1016/j.neuron.2015.10.020#mmc9.

AUTHOR CONTRIBUTIONS

Conceptualization, N.N. and M.P.M.; Methodology, N.N. and M.P.M.; Investigation, NN; Formal Analysis, N.N. and M.P.M.; Writing – Original Draft, N.N. and M.P.M.; Writing – Review & Editing, N.N. and M.P.M.; Funding Acquisition, M.P.M.

ACKNOWLEDGMENTS

We thank T. Becker and C. Becker for kindly giving us the *astray* mutant allele, J. Clarke for the a-PH-3 antibody, C. Houart for the a-HuC/D antibody, and J. Bonkowsky for the FoxP2.A enhancer construct. We thank the King's College London fish facility staff at Guy's Hospital campus for their excellent fish husbandry and care. We also thank G. Murphy, M. Grubb, O. Marin, and C. Larsen for comments on the manuscript. This study was supported by a BBSRC project grant (BB/L004992/1) awarded to M.P.M.

Received: July 9, 2015
Revised: September 17, 2015
Accepted: October 8, 2015
Published: November 19, 2015

REFERENCES

Ahrens, M.B., Orger, M.B., Robson, D.N., Li, J.M., and Keller, P.J. (2013). Whole-brain functional imaging at cellular resolution using light-sheet microscopy. Nat. Methods *10*, 413–420.

Baier, H. (2013). Synaptic laminae in the visual system: molecular mechanisms forming layers of perception. Annu. Rev. Cell Dev. Biol. *29*, 385–416.

Ben Fredj, N., Hammond, S., Otsuna, H., Chien, C.B., Burrone, J., and Meyer, M.P. (2010). Synaptic activity and activity-dependent competition regulates axon arbor maturation, growth arrest, and territory in the retinotectal projection. J. Neurosci. *30*, 10939–10951.

Bonkowsky, J.L., Wang, X., Fujimoto, E., Lee, J.E., Chien, C.B., and Dorsky, R.I. (2008). Domain-specific regulation of foxP2 CNS expression by lef1. BMC Dev. Biol. *8*, 103.

Branco, T., and Häusser, M. (2010). The single dendritic branch as a fundamental functional unit in the nervous system. Curr. Opin. Neurobiol. *20*, 494–502.

Brierley, D.J., Blanc, E., Reddy, O.V., Vijayraghavan, K., and Williams, D.W. (2009). Dendritic targeting in the leg neuropil of Drosophila: the role of midline signalling molecules in generating a myotopic map. PLoS Biol. *7*, e1000199.

Burrill, J.D., and Easter, S.S., Jr. (1994). Development of the retinofugal projections in the embryonic and larval zebrafish (Brachydanio rerio). J. Comp. Neurol. *346*, 583–600.

Campbell, D.S., Stringham, S.A., Timm, A., Xiao, T., Law, M.Y., Baier, H., Nonet, M.L., and Chien, C.B. (2007). Slit1a inhibits retinal ganglion cell arborization and synaptogenesis via Robo2-dependent and -independent pathways. Neuron *55*, 231–245.

Chen, T.W., Wardill, T.J., Sun, Y., Pulver, S.R., Renninger, S.L., Baohan, A., Schreiter, E.R., Kerr, R.A., Orger, M.B., Jayaraman, V., et al. (2013). Ultrasensitive fluorescent proteins for imaging neuronal activity. Nature *499*, 295–300.

Chklovskii, D.B., and Koulakov, A.A. (2004). Maps in the brain: what can we learn from them? Annu. Rev. Neurosci. *27*, 369–392.

Dhande, O.S., and Huberman, A.D. (2014). Retinal ganglion cell maps in the brain: implications for visual processing. Curr. Opin. Neurobiol. *24*, 133–142.

Dräger, U.C. (1981). Observations on the organization of the visual cortex in the reeler mouse. J. Comp. Neurol. *201*, 555–570.

Duan, X., Krishnaswamy, A., De la Huerta, I., and Sanes, J.R. (2014). Type II cadherins guide assembly of a direction-selective retinal circuit. Cell *158*, 793–807.

Fleisch, V.C., and Neuhauss, S.C. (2006). Visual behavior in zebrafish. Zebrafish *3*, 191–201.

Fricke, C., Lee, J.S., Geiger-Rudolph, S., Bonhoeffer, F., and Chien, C.B. (2001). astray, a zebrafish roundabout homolog required for retinal axon guidance. Science *292*, 507–510.

Gabriel, J.P., Trivedi, C.A., Maurer, C.M., Ryu, S., and Bollmann, J.H. (2012). Layer-specific targeting of direction-selective neurons in the zebrafish optic tectum. Neuron *76*, 1147–1160.

Grama, A., and Engert, F. (2012). Direction selectivity in the larval zebrafish tectum is mediated by asymmetric inhibition. Front. Neural Circuits *6*, 59.

Guy, J., Wagener, R.J., Mock, M., and Staiger, J.F. (2015). Persistence of functional sensory maps in the absence of cortical layers in the somsatosensory cortex of reeler mice. Cereb. Cortex *25*, 2517–2528.

Hoegler, K.J., and Horne, J.H. (2010). Targeting the zebrafish optic tectum using in vivo electroporation. Cold Spring Harb. Protoc. *2010*, t5463.

Huberman, A.D., Manu, M., Koch, S.M., Susman, M.W., Lutz, A.B., Ullian, E.M., Baccus, S.A., and Barres, B.A. (2008). Architecture and activity-mediated refinement of axonal projections from a mosaic of genetically identified retinal ganglion cells. Neuron *59*, 425–438.

Hunter, P.R., Lowe, A.S., Thompson, I.D., and Meyer, M.P. (2013). Emergent properties of the optic tectum revealed by population analysis of direction and orientation selectivity. J. Neurosci. *33*, 13940–13945.

Kaethner, R.J., and Stuermer, C.A. (1992). Dynamics of terminal arbor formation and target approach of retinotectal axons in living zebrafish embryos: a time-lapse study of single axons. J. Neurosci. *12*, 3257–3271.

Kaethner, R.J., and Stuermer, C.A. (1997). Dynamics of process formation during differentiation of tectal neurons in embryonic zebrafish. J. Neurobiol. *32*, 627–639.

Kim, I.J., Zhang, Y., Meister, M., and Sanes, J.R. (2010). Laminar restriction of retinal ganglion cell dendrites and axons: subtype-specific developmental patterns revealed with transgenic markers. J. Neurosci. *30*, 1452–1462.

Krishnaswamy, A., Yamagata, M., Duan, X., Hong, Y.K., and Sanes, J.R. (2015). Sidekick 2 directs formation of a retinal circuit that detects differential motion. Nature *524*, 466–470.

Lemmon, V., and Pearlman, A.L. (1981). Does laminar position determine the receptive field properties of cortical neurons? A study of corticotectal cells in area 17 of the normal mouse and the reeler mutant. J. Neurosci. *1*, 83–93.

Lowe, A.S., Nikolaou, N., Hunter, P.R., Thompson, I.D., and Meyer, M.P. (2013). A systems-based dissection of retinal inputs to the zebrafish tectum reveals different rules for different functional classes during development. J. Neurosci. *33*, 13946–13956.

Matsuoka, R.L., Nguyen-Ba-Charvet, K.T., Parray, A., Badea, T.C., Chédotal, A., and Kolodkin, A.L. (2011). Transmembrane semaphorin signalling controls laminar stratification in the mammalian retina. Nature *470*, 259–263.

Meyer, M.P., and Smith, S.J. (2006). Evidence from in vivo imaging that synaptogenesis guides the growth and branching of axonal arbors by two distinct mechanisms. J. Neurosci. *26*, 3604–3614.

Niell, C.M., and Smith, S.J. (2005). Functional imaging reveals rapid development of visual response properties in the zebrafish tectum. Neuron *45*, 941–951.

Niell, C.M., Meyer, M.P., and Smith, S.J. (2004). In vivo imaging of synapse formation on a growing dendritic arbor. Nat. Neurosci. *7*, 254–260.

Nikolaou, N., Lowe, A.S., Walker, A.S., Abbas, F., Hunter, P.R., Thompson, I.D., and Meyer, M.P. (2012). Parametric functional maps of visual inputs to the tectum. Neuron *76*, 317–324.

Pérez-Escudero, A., and de Polavieja, G.G. (2007). Optimally wired subnetwork determines neuroanatomy of Caenorhabditis elegans. Proc. Natl. Acad. Sci. USA *104*, 17180–17185.

Rivera-Alba, M., Vitaladevuni, S.N., Mishchenko, Y., Lu, Z., Takemura, S.Y., Scheffer, L., Meinertzhagen, I.A., Chklovskii, D.B., and de Polavieja, G.G. (2011). Wiring economy and volume exclusion determine neuronal placement in the Drosophila brain. Curr. Biol. *21*, 2000–2005.

Robles, E., Filosa, A., and Baier, H. (2013). Precise lamination of retinal axons generates multiple parallel input pathways in the tectum. J. Neurosci. *33*, 5027–5039.

Sanes, J.R., and Yamagata, M. (2009). Many paths to synaptic specificity. Annu. Rev. Cell Dev. Biol. *25*, 161–195.

Sanes, J.R., and Zipursky, S.L. (2010). Design principles of insect and vertebrate visual systems. Neuron *66*, 15–36.

Simmons, P.A., and Pearlman, A.L. (1983). Receptive-field properties of transcallosal visual cortical neurons in the normal and reeler mouse. J. Neurophysiol. *50*, 838–848.

Simpson, H.D., Kita, E.M., Scott, E.K., and Goodhill, G.J. (2013). A quantitative analysis of branching, growth cone turning, and directed growth in zebrafish retinotectal axon guidance. J. Comp. Neurol. *521*, 1409–1429.

Xiao, T., Staub, W., Robles, E., Gosse, N.J., Cole, G.J., and Baier, H. (2011). Assembly of lamina-specific neuronal connections by slit bound to type IV collagen. Cell *146*, 164–176.

Yamagata, M., and Sanes, J.R. (2008). Dscam and Sidekick proteins direct lamina-specific synaptic connections in vertebrate retina. Nature *451*, 465–469.

Yamagata, M., Weiner, J.A., and Sanes, J.R. (2002). Sidekicks: synaptic adhesion molecules that promote lamina-specific connectivity in the retina. Cell *110*, 649–660.

Neuron

Supplemental Information

Lamination Speeds the Functional Development of Visual Circuits

Nikolas Nikolaou and Martin P. Meyer

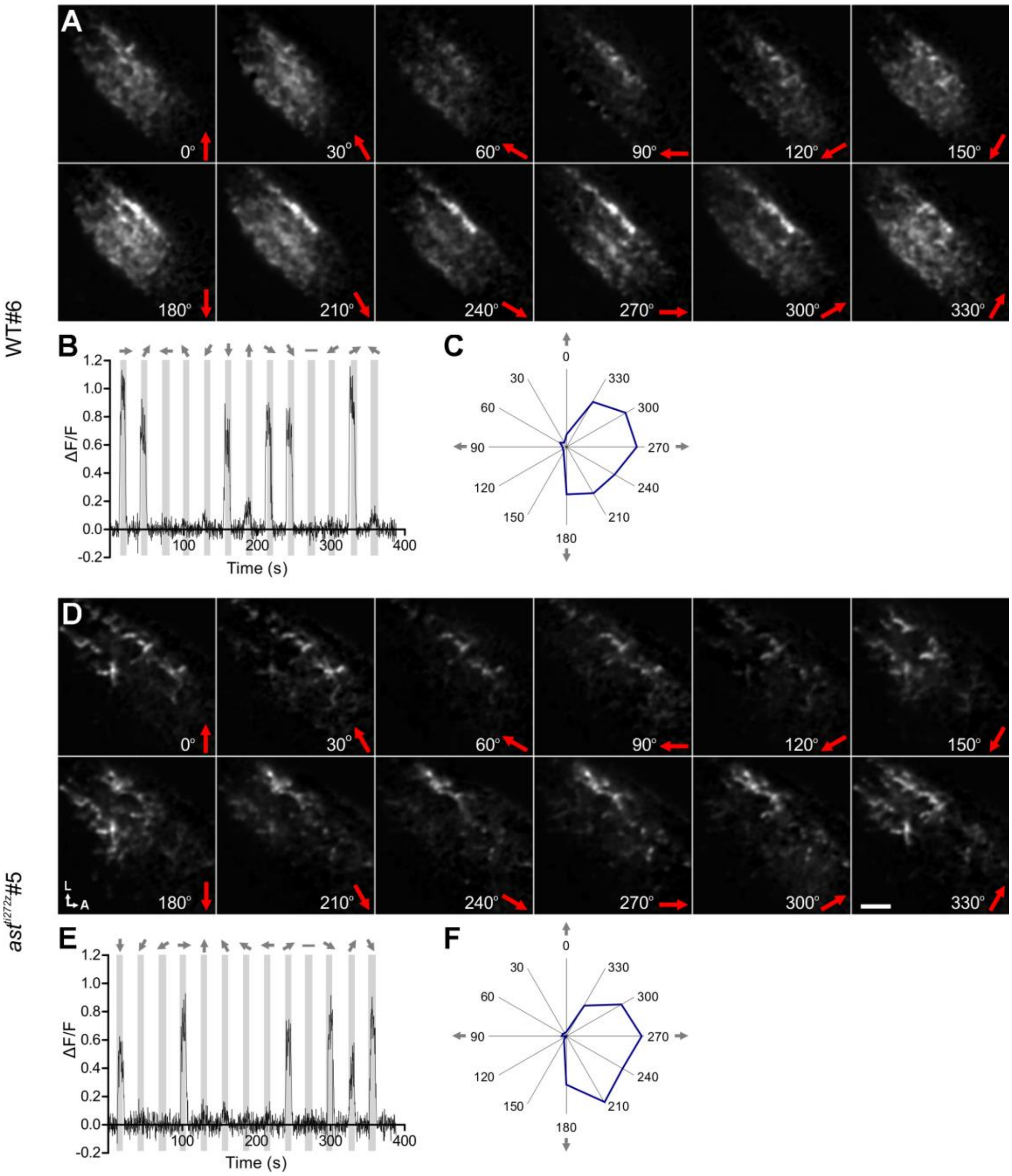

Figure S1. Responses of SyGCaMP3-expressing RGC axons terminating in the optic tectum (related to Figure 1). Examples of tuning experiments from WT (A-C) and *ast*^{ti272z} (D-F) *Tg(Isl2b:Gal4;UAS:SyGCaMP3)* larvae at 7 dpf are shown. (A, D) Montages showing integral responses (grayscale) of all RGC axons in the tectum. Direction of motion is shown on the bottom right in each panel. Scale bar represents 20 µm. A, anterior; L, lateral. (B, E) Representative response of single voxels during a tuning experiment. Stimulus epochs are shown in gray and direction of motion is indicated by arrows on the top. (C, F) Integral responses from (B) and (E) are shown in polar plot form.

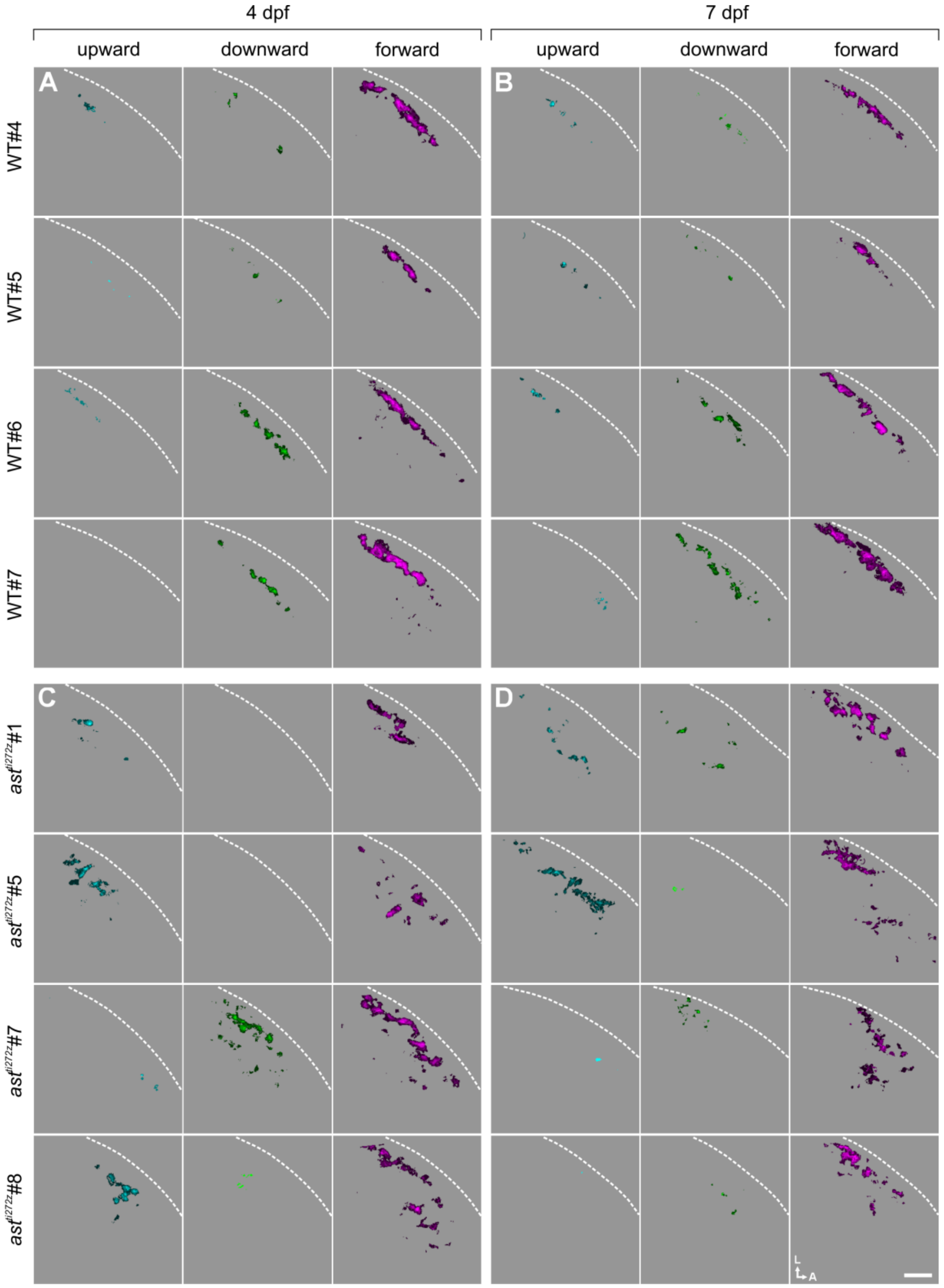

Figure S2. DS-RGC axons are diffusely distributed in the tectal neuropil of individual *astray* mutants (related to Figure 2). (A-D) Functional maps generated from single *Tg(Isl2b:Gal4;UAS:SyGCaMP3)* larvae showing the spatial distribution of DS-RGC subtypes in the tectal neuropil of WT larvae at 4 dpf (A) and 7 dpf (B), and *ast*^{ti272z} larvae at 4 dpf (C) and 7 dpf (D). Four examples of WT and *ast*^{ti272z} larvae are shown, respectively. Voxel brightness is proportional to the summed incidence of each functional subtype across all experiments

performed in each individual fish. Dashed lines indicate the position of the skin overlaying the tectum. Scale bar represents 20 µm. A, anterior; L, lateral.

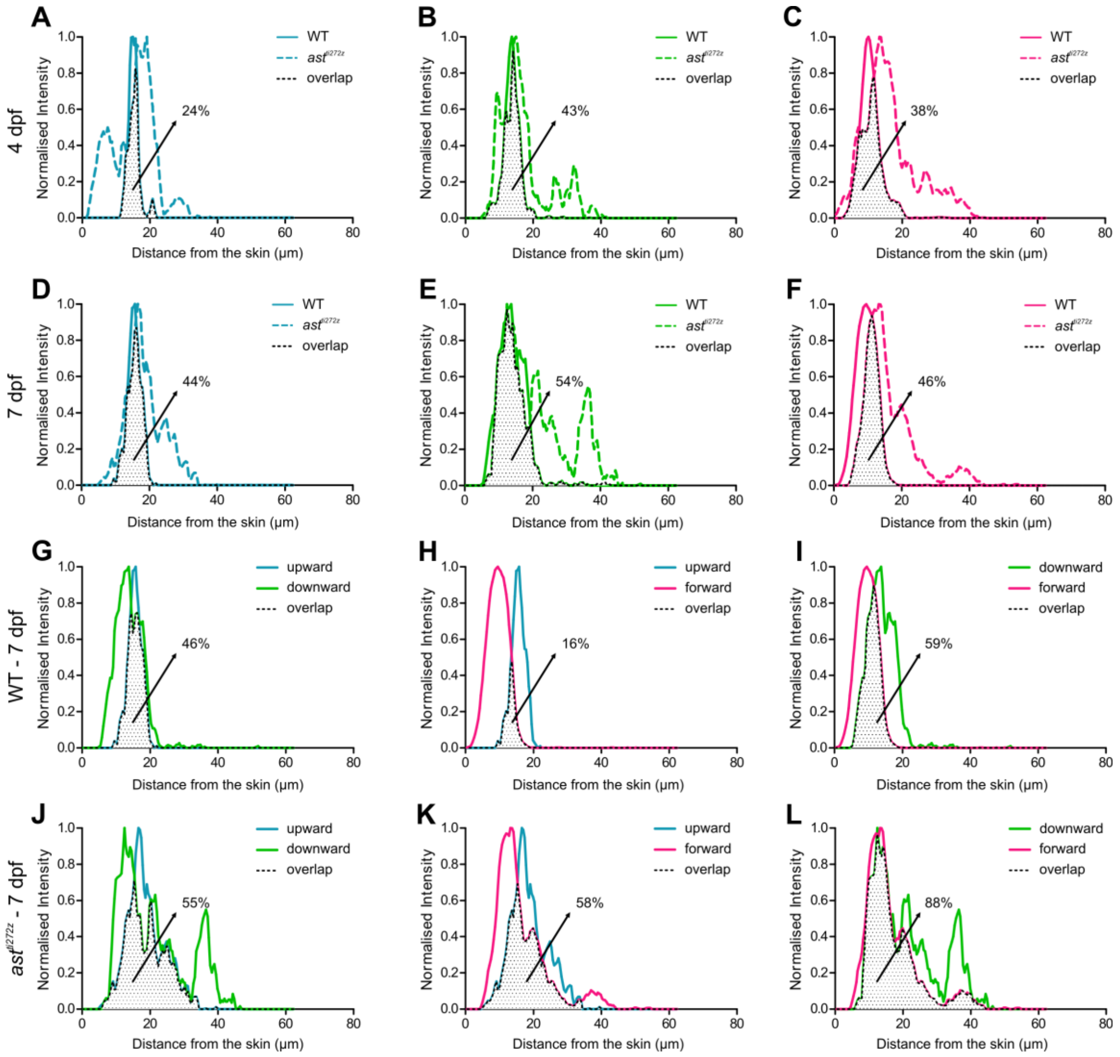

Figure S3. The lamination and segregation of DS-RGC axons is perturbed in *astray* mutants (related to Figure 2). Line plots generated from the composite parametric maps in Figures 2A-2D comparing the lamination (A-F) and segregation (G-L) of DS-RGC subtypes in WT (n = 8, total of 24 optical sections) and ast^{ti272z} (n = 9, total of 27 optical sections) tecta at 4 and 7 dpf. All plots show normalized intensities as a function of distance from the skin. (A-F) Pairwise comparison showing the degree of overlap for upward (A and D), downward (B and E) and forward (C and F) DS-RGC subtypes at 4 dpf (A-C) and 7 dpf (D-F). Dotted area represents the area of intersection between the WT and ast^{ti272z} domains. Values shown represent the fraction of all DS voxels that are located in the correct location in ast^{ti272z} tecta. (G-L) Pairwise comparisons showing the degree of spatial overlap between upward and downward (G and J), upward and forward (H and K) and downward and forward (I and L) DS-RGC subtypes within WT (G-I) and ast^{ti272z} (J-L) tecta at 7 dpf. Doted area represents the area of intersection between the two subtypes. Values shown represent the fraction of downward (for G and J) or forward (for H, I, K and L) DS voxels that spatially overlap with upward or downward DS voxels.

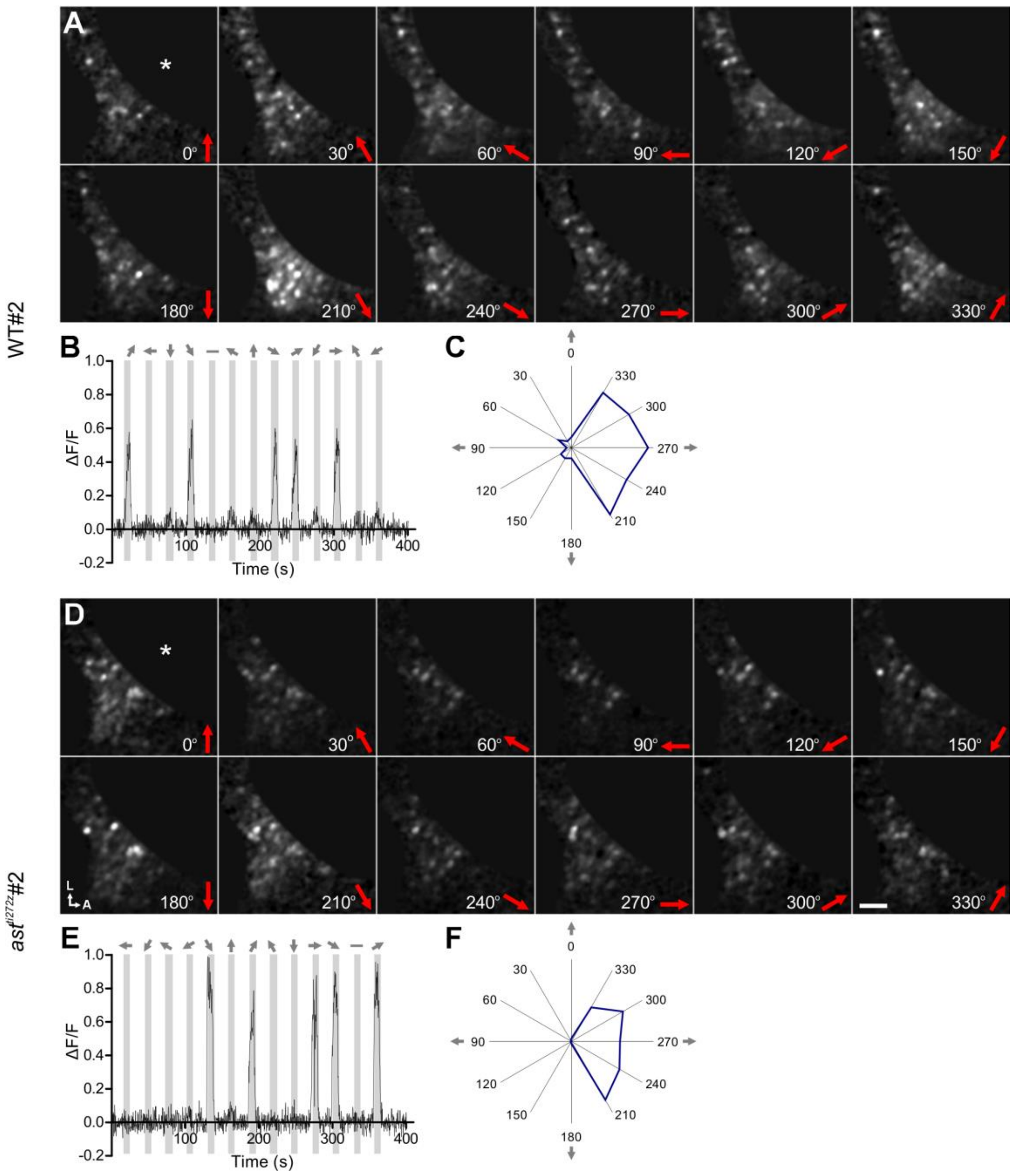

Figure S4. Responses of GCaMP5G-expressing tectal neurons in the optic tectum (related to Figure 3). Examples of tuning experiments from individual WT (A-C) and *ast*^{ti272z} (D-F) *Tg(elavl3:GCaMP5G)* larvae at 7 dpf are shown. Asterisks indicate the neuropil area of the tectum which has been removed for analysis purposes. (A, D) Montages showing integral responses (grayscale) of all voxels (tectal cell body area) in the tectum. Direction of motion is shown on the bottom right in each panel. Scale bar represents 30 µm. A, anterior; L, lateral. (B, E) Representative response of single voxels during a tuning experiment. Stimulus epochs are shown in gray and direction of motion is indicated by arrows on the top. (C, F) Integral responses from (B) and (E) are shown in polar plot form.

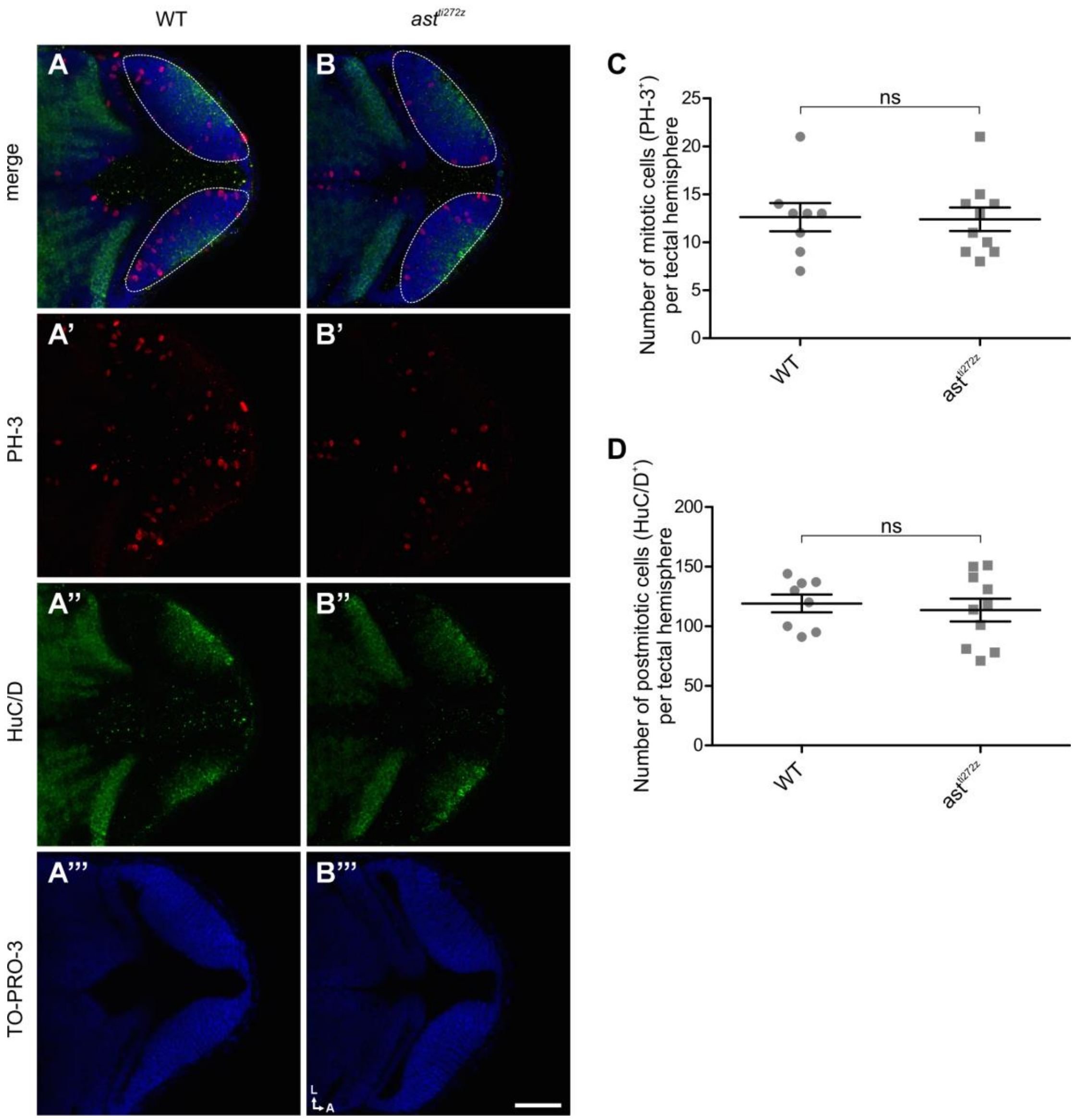

Figure S5. Tectal proliferation and differentiation are unaffected in *astray* mutants (related to Figure 3). Measurement of proliferation and differentiation in WT (n = 4) and ast^{ti272z} (n = 5) larvae at 48 hpf. (A-B) Single confocal sections showing dorsal views of WT (A) and ast^{ti272z} (B) embryos immunostained for PH-3 and HuC/D to mark mitotic and postmitotic cells, respectively. TO-PRO-3 was also used to stain cell nuclei. Dashed line outlines the border of each tectal hemisphere. Scale bar represents 50 µm. A, anterior; L, lateral. (C-D) Quantification of the degree of proliferation and differentiation in the tectum. For the number of mitotic cells (PH-3$^+$) (C) 12.6 ± 1.4 for WT and 12.4 ± 1.2 for ast^{ti272z}; for the number of post-mitotic cells (HuC/D$^+$) (D) 119.1 ± 7.4 for WT and 113.6 ± 9.4 for ast^{ti272z}. All graphs show mean values ± SEM. ns, not significant, unpaired t-test.

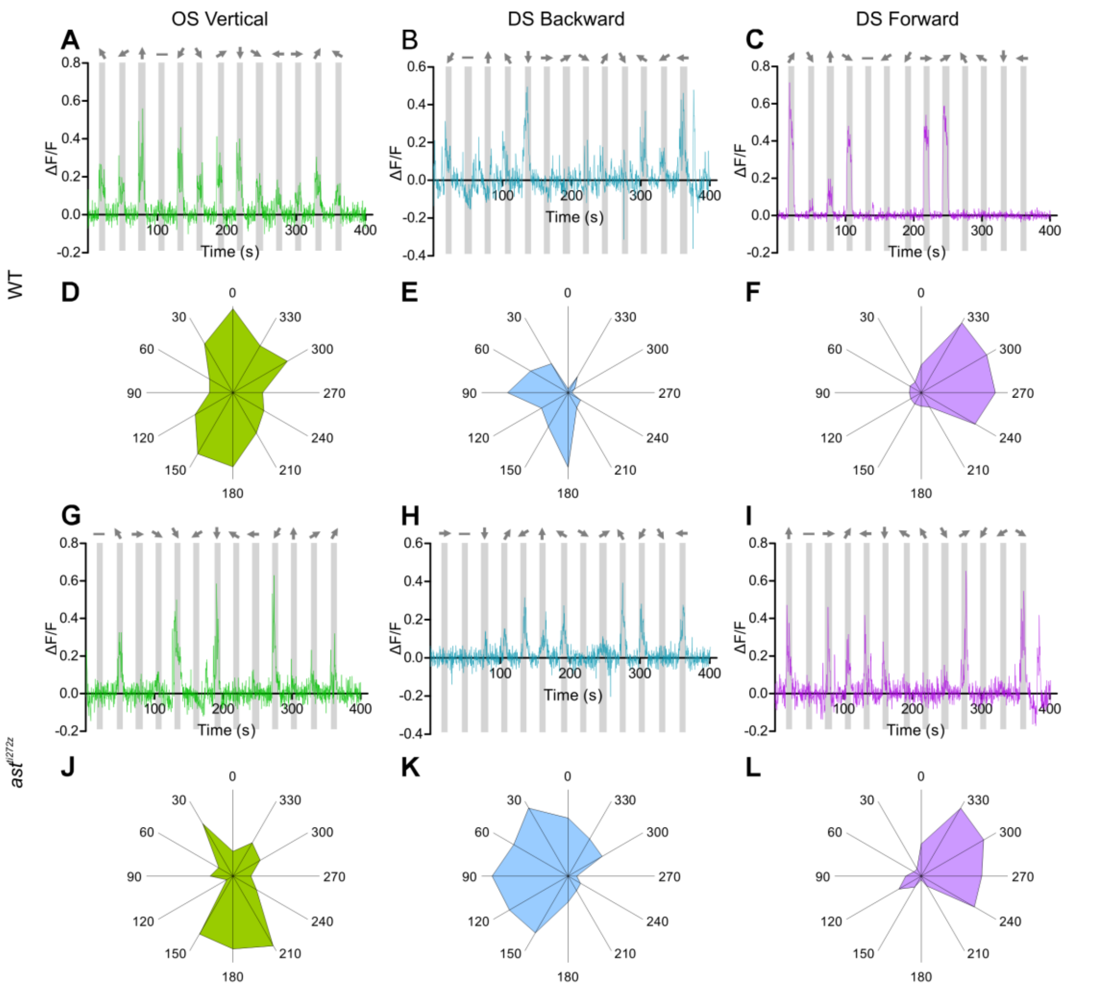

Figure S6. Responses of individual GCaMP6F-expressing FoxP2.A tectal cells (related to Figure 4). Examples of tuning experiments from individual FoxP2.A tectal cells in WT (A-F) and *ast*^{ti272z} (G-L) larvae at 7 dpf. OS vertical, DS backward and DS forward tuned cells from control and experimental groups are shown. (A, B, C, G, H, I) Cell body responses during a tuning experiment. Stimulus epochs are shown in gray and direction of motion is indicated by arrows on the top. (D, E, F, J, K, L) Integral responses are shown in polar plot form. Response traces and polar plots are color-coded accordingly to match the color-coding scheme used to describe the functional classes in Figure 4.

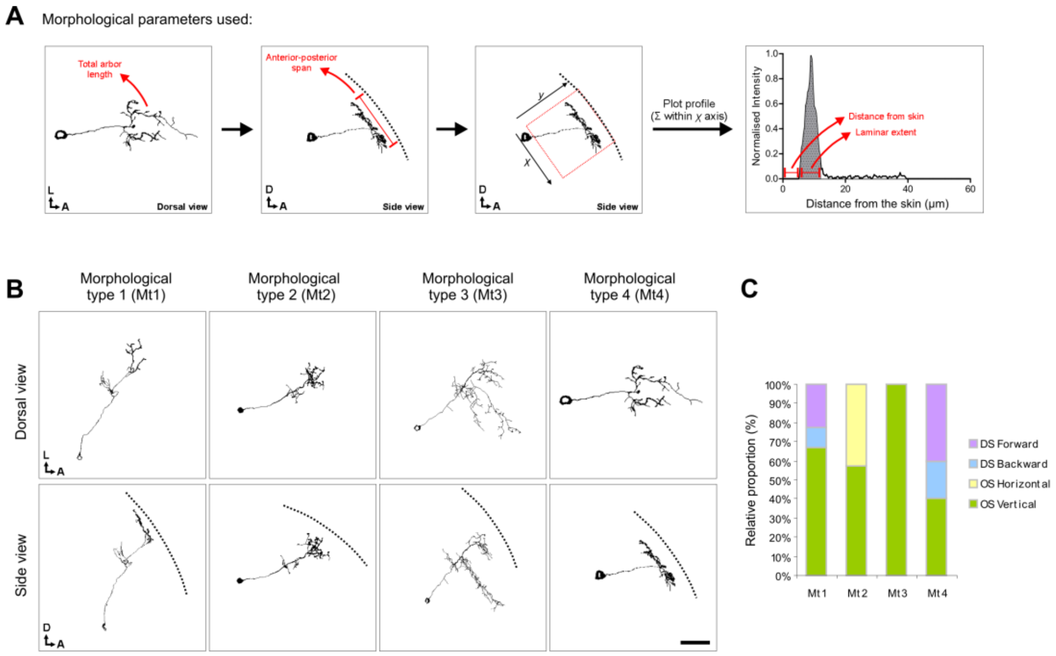

Figure S7. Morphological analysis of FoxP2.A labeled tectal cells (related to Figure 4). (A) Morphological parameters used to characterize FoxP2.A tectal cells and the process followed to extract them. Labeled tectal arbors were traced and total arbor length was calculated by summing the length of all individual traced fragments. Cells were reconstructed in three dimensions and then rotated to obtain an orthogonal view. From this view, an anterior-posterior span was measured. To obtain the other two parameters a rectangular region of interest was drawn across a relatively straight stretch of the tectal neuropil to cover the entire neurite. The fluorescence intensity across tectal depth was then calculated and the derived line plots were used to measure the distance from the skin and the laminar extent of distal arbors. A, anterior; D, dorsal; L, lateral. (B-C) Clustering of WT FoxP2.A responsive cells (n = 24 in 22 larvae) based on their morphological parameters was performed using k-means clustering. In total four morphological types are present (B), with each morphological cluster containing one or more functional subtypes (C). Scale bar represents 30 µm. A, anterior; D, dorsal; L, lateral.

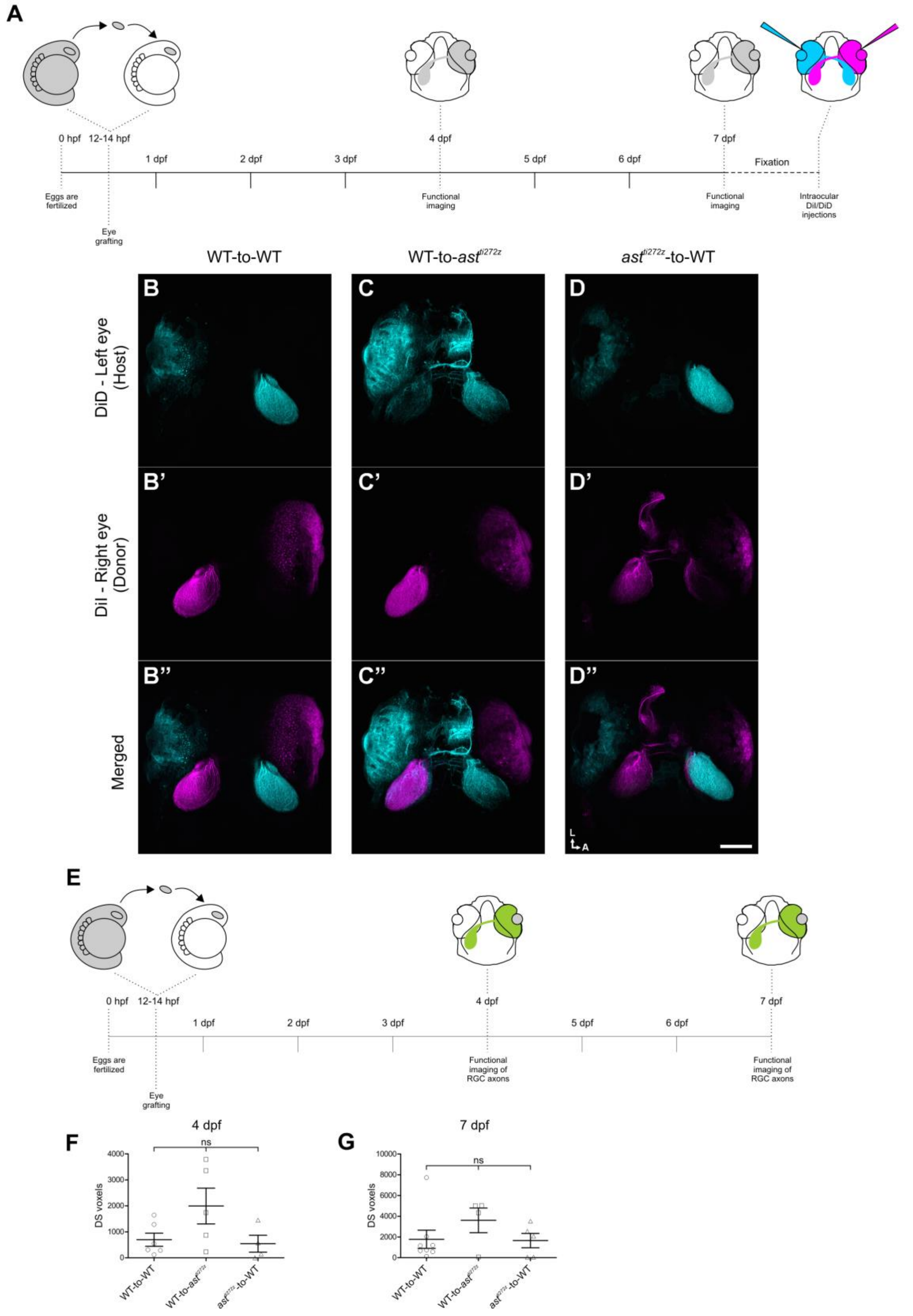

Figure S8. Eye transplantations as a means to assess the cellular requirements for Robo2 in the functional development of DS tectal cells (related to Figure 6). (A) Schematic showing the experimental procedure followed. Optic vesicles from donor embryos were transplanted into hosts at 12-14 hpf and functional imaging was performed at 4 and 7 dpf. To confirm the success of each transplantation experiment, samples were fixed post-functional imaging at

7dpf and the donor and host eyes were bulk-loaded with the lipophilic dyes, DiI and DiD respectively, in order to label the entire retinotectal projection. (B-D) Three transplantation examples (one per transplantation group) showing the retinotectal projection of host and donor eyes. Scale bar represents 100 µm. A, anterior; L, lateral. (E) Schematic showing the experimental procedure followed to investigate the functional development of donor DS-RGC axons. Optic vesicles from *Tg(Isl2b:Gal4;UAS:SyGCaMP3)* donor embryos were transplanted into hosts at 12-14 hpf and functional imaging of donor RGC axons within the tectum was performed at 4 and 7 dpf. (F-G) Quantification of average number of DS-RGC voxels per group. Total number of DS-RGC voxels at 4 dpf (F), 697 ± 252.6 for WT-to-WT (n = 6 larvae, total of 18 optical sections), 1996 ± 689.9 for WT-to-ast^{ti272z} (n = 5 larvae, total of 15 optical sections) and 544.3 ± 325.4 for ast^{ti272z}-to-WT (n = 4 larvae, total of 12 optical sections). Total number of DS cells at 7 dpf (G), 1783 ± 870.9 for WT-to-WT (n = 8 larvae, total of 24 optical sections), 3606 ± 1190 for WT-to-ast^{ti272z} (n = 4 larvae, total of 12 optical sections) and 1653 ± 692.9 for ast^{ti272z}-to-WT (n = 5 larvae, total of 15 optical sections). All graphs show mean values ± SEM. ns, not significant, Kruskal-Wallis and Dunn's multiple comparison tests.

	4 dpf			7 dpf		
	wt Mean ± SEM	*ast*$^{-/-}$ Mean ± SEM	*P* value	wt Mean ± SEM	*ast*$^{-/-}$ Mean ± SEM	*P* value
UPWARD **subpopulation**	56.5 ± 26.9	545.9 ± 257.1	0.095 ns	128.6 ± 39	455.7 ± 305.7	0.333 ns
DOWNWARD **subpopulation**	507.5 ± 191	316.2 ± 258.1	0.568 ns	618.1 ± 192.7	345.1 ± 139.6	0.261 ns
FORWARD **subpopulation**	3613 ± 667.4	2009 ± 705.4	0.121 ns	4259 ± 832.9	2067 ± 666.4	0.055 ns
TOTAL **population**	5237 ± 919	3624 ± 1283	0.333 ns	6409 ± 1266	4114 ± 1205	0.209 ns
UPWARD **bandwidth**	86.5 ± 8.8	85.3 ± 6	0.91 ns	92.9 ± 5.5	98.9 ± 6.1	0.483 ns
DOWNWARD **bandwidth**	98.3 ± 4.6	102.8 ± 12.5	0.703 ns	101.7 ± 4.3	90.7 ± 4.8	0.114 ns
FORWARD **bandwidth**	104.4 ± 1.8	92.6 ± 3	0.005 **	97 ± 1.6	95.3 ± 1.8	0.499 ns

Table S1. The functional response properties of DS-RGCs are normal in *astray* mutants (related to Figure 1). Comparison of the size of each DS subpopulation and their tuning bandwidth between WT (n = 8, total of 24 optical sections) and *ast*^{ti272z} (n = 9, total of 27 optical sections) larvae at 4 and 7 dpf. Values show mean ± SEM. **p < 0.01; ns, not significant, unpaired t-test.

	7 dpf		
	wt<br>Mean ± SEM	*ast*<br>Mean ± SEM	*P*<br>value
**UPWARD subpopulation**	1.3 ± 0.4	1.5 ± 0.8	0.801<br>ns
**BACKWARD subpopulation**	3.1 ± 1	3.3 ± 1.2	0.919<br>ns
**DOWNWARD subpopulation**	1.1 ± 0.4	1.2 ± 0.5	0.907<br>ns
**FORWARD subpopulation**	6.2 ± 1.5	8.7 ± 2.6	0.428<br>ns
**TOTAL population**	14.8 ± 2.9	18.1 ± 4.8	0.561<br>ns
**UPWARD bandwidth**	81.1 ± 5.1	97.6 ± 8.7	0.1141<br>ns
**BACKWARD bandwidth**	93.6 ± 5.4	94.5 ± 8	0.9294<br>ns
**DOWNWARD population**	90.6 ± 9	83.7 ± 7.7	0.5817<br>ns
**FORWARD bandwidth**	91.1 ± 2.3	86.5 ± 4.4	0.3456<br>ns

Table S2. The functional response properties of DS tectal cells in *astray* mutants are indistinguishable from WT animals at 7 dpf (related to Figure 3). Comparison of the size of each DS subpopulation and their tuning bandwidth between WT (n = 12, total of 36 optical sections) and *ast*^{ti272z} (n = 12, total of 36 optical sections) tecta at 7 dpf. Values show mean ± SEM. ns, not significant, unpaired t-test.

Video S1. Calcium responses of SyGCaMP3-expressing RGC axons in the WT optic tectum evoked by drifting bar stimulus (related to Figure 1). Tuning experiment from a *Tg(Isl2b:Gal4;UAS:SyGCaMP3)* larva (WT#6) summarized as a montage in Figure S1A. Raw fluorescence (left) and normalized ΔF/F (right) responses of SyGCaMP3-expressing RGC axons in the tectal neuropil evoked by drifting bar stimulus are shown. Movie encompasses an entire tuning experiment in which all 12 directions of bar motion are presented.

Video S2. Calcium responses of SyGCaMP3-expressing RGC axons in the *astray* optic tectum evoked by drifting bar stimulus (related to Figure 1). Tuning experiment from a *Tg(Isl2b:Gal4;UAS:SyGCaMP3)* larva (*ast*^{ti272z}#5) summarized as a montage in Figure S1D. Raw fluorescence (left) and normalized ΔF/F (right) responses of SyGCaMP3-expressing RGC axons in the tectal neuropil evoked by drifting bar stimulus are shown. Movie encompasses an entire tuning experiment in which all 12 directions of bar motion are presented.

Video S3. Calcium responses of GCaMP5G-expressing tectal neurons in the WT optic tectum evoked by drifting bar stimulus (related to Figure 3). Tuning experiment from a *Tg(elavl3:GCaMP5G)* larva (WT#2) summarized as a montage in Figure S4A. Raw fluorescence (left) and normalized ΔF/F (right) responses of GCaMP5G-expressing tectal cells in the tectum evoked by drifting bar stimulus are shown. Movie encompasses an entire tuning experiment in which all 12 directions of bar motion are presented.

Video S4. Calcium responses of GCaMP5G-expressing tectal neurons in the *astray* optic tectum evoked by drifting bar stimulus (related to Figure 3). Tuning experiments from a *Tg(elavl3:GCaMP5G)* larva (*ast*^{ti272z}#2) that are presented as a montage in Figure S4D. Raw fluorescence (left) and normalized ΔF/F (right) responses of GCaMP5G-expressing tectal cells in the tectum evoked by drifting bar stimulus are shown. Movie encompasses an entire tuning experiment in which all 12 directions of bar motion are presented.

Video S5. Calcium responses in a single GCaMP6F-expressing FoxP2.A tectal cell evoked by drifting bar stimulus (related to Figure 4). Time-lapse data showing the raw fluorescence (left) and normalized ΔF/F (right) responses of a single GCaMP6F-expressing tectal cell in the tectum evoked by drifting bar stimulus. Movie encompasses an entire tuning experiment in which all 12 directions of bar motion are presented.

Video S6. Three-dimensional reconstructions of FoxP2.A labeled tectal cells (related to Figure 4). Traced FoxP2.A tectal cells were reconstructed as shown. An example of a forward tuned DS tectal neuron in WT (top) and a*st*^{ti272z} (bottom) tectum is shown.

Supplemental Experimental Procedures

Animals

Zebrafish were reared at 28.5°C on a 14 hr light/10 hr dark cycle. Embryos produced by natural crosses were staged by hours, or days post-fertilization (hpf, or dpf) and raised in Danieau's solution (58 mM NaCl, 0.7 mM KCl, 0.4 mM $MgSO_4$, 0.6 mM $Ca(NO_3)_2$, 5.0 mM HEPES, pH 7.2). Transgenic lines utilized in this study include *Tg(Isl2b:Gal4)zc60* (Ben Fredj et al., 2010), *Tg(UAS:SyGCaMP3)kg1* (Nikolaou et al., 2012) and *Tg(elavl3:GCaMP5G)a4598* (Ahrens et al., 2013). The *robo2* mutant allele used in this study was ast^{ti272z} (Fricke et al., 2001). Functional imaging experiments were performed in the pigmentation mutant $mitfa^{w2}$, which lacks all neural crest derived melanophores (Lister et al., 1999). This work was approved by the local Animal Care and Use Committee (King's College London), and was carried out in accordance with the Animals (Experimental Procedures) Act, 1986, under license from the United Kingdom Home Office.

Generation of DNA Plasmid Constructs

To make the *FoxP2.A:Gal4FF* construct, a plasmid containing the 9.7kb *FoxP2-enhancerA* fragment (*p5EFoxP2.A*) (Bonkowsky et al., 2008), (provided by Joshua Bonkowsky, University of Utah), was digested with *BsrGI* and a basal promoter (containing the adenovirus *E1B TATA* box) was inserted to generate a *p5EFoxP2.Abas* plasmid. A LR reaction (Gateway cloning system, Invitrogen) was performed in order to fuse *FoxP2.Abas* with *Gal4FF* (*pMEGal4FF*) in a plasmid containing Tol2 sites (*pDestTol2R4R2pA*) (Villefranc et al., 2007), thus generating a *pTolFoxP2.Abas:Gal4FF* plasmid. To make the *5UAS:GCaMP6F* construct, the genetically encoded calcium indicator, *GCaMP6F* (Chen et al., 2013) was PCR amplified and directionally cloned into the *BglII/NotI* sites downstream of the upstream activator sequence (*UAS*) motifs of the *5UAS:EGFP-N2* plasmid (Ben Fredj et al., 2010) thus replacing the *EGFP* sequence.

DNA Electroporations

Tectal cells were mosaically labeled by co-electroporating the *FoxP2.A:Gal4FF* activator plasmid with effector plasmids, where expression of either GCaMP6F or tandem-dimer tomato (tdTomato) is driven by *5UAS* motifs (Ben Fredj et al., 2010). Electroporations were performed as previously described (Hoegler and Horne, 2010). Briefly, 4 dpf zebrafish larvae were mounted in 1% low melting point agarose (Sigma) in electroporation buffer (180 mM NaCl, 5 mM KCl, 1.8 mM $CaCl_2$, 5 mM HEPES, pH 7.2). Plasmid DNA was prepared using midi-prep kits (Qiagen) and pressure-injected into the midbrain ventricle at a concentration of 500 ng/µl each. Electroporation electrodes were positioned such that the positive electrode is lateral and slightly dorsal to the hemisphere of the optic tectum to be targeted and the negative electrode lateral and ventral to the opposite eye of the larvae. DNA plasmids were electroporated by delivering 1 second trains of 5 ms, 85 V voltage pulses at 200 Hz using an SD9 stimulator (Grass Instruments). A total of five trains per larva were delivered.

Enucleations

Embryos were collected and raised at 22°C until the 6-10 somite stage (12-14 hpf). Embryos were then mounted in 1.3% low melting point agarose (Sigma) in fish Ringer´s (116 mM NaCl,

3 mM KCl, 4 mM $CaCl_2$, 1 mM $MgCl_2$, 5 mM HEPES, 20 µg/ml gentamycin sulfate, pH 7.2). A small window was opened in the epidermis above the right eye primordia using a drop of mineral oil (Sigma), through which the optic vesicles can be reached. The optic vesicles were removed using sharpened tungsten micro-needles and a wire microloop. 1 hour after enucleation, the embryos were removed from the agarose and raised under standard conditions in Danieau's.

Eye transplantations

Eye transplantations were performed as previously described (Picker et al., 1999). Briefly, age-matched host and donor embryos were collected and raised at 22°C until the 6-10 somite stage (12-14 hpf). Host and donor embryos were mounted in 1.3% low melting point agarose (Sigma) in fish Ringer´s (116 mM NaCl, 3 mM KCl, 4 mM $CaCl_2$, 1 mM $MgCl_2$, 5 mM HEPES, 20 µg/ml gentamycin sulfate, pH 7.2). A small window was opened in the epidermis above the right eye primordia using a drop of mineral oil (Sigma), through which the optic vesicles can be reached. The host optic vesicle was removed and the donor optic vesicle inserted using sharpened tungsten micro-needles and a wire microloop. 1 hour after transplantation, when healing was complete, the embryos were removed from the agarose and raised under standard conditions in Danieau's. At the end of the experiment, the host and donor eyes were labeled with different lipophilic dyes (see DiI/DiD labeling below).

Visual stimulation and functional imaging

Non-anaesthetized larvae were immobilized in 2% low melting point agarose (Sigma) prepared in Danieau's solution and mounted dorsal side up on a raised glass platform that was placed in a custom-made Danieau-filled chamber. The agarose was sufficient to restrain the larvae so that anesthesia was not required. A diffusive filter 3026 (Rosco, Inc., Hollywood, CA) was bonded to one side of the chamber and which served as a projection screen. The agarose surrounding the eye facing the projection screen was removed allowing an unobstructed view of the projected image on the side of the chamber, while permitting the objective to be positioned above the tectum for imaging. Larvae were positioned 3 cm away from the screen and the projected image filled a visual field of approximately 97° by 63°. Visual stimuli consisted of light (56 cd/m^2) or dark bars (8 cd/m^2) (75% and 25% of mean respectively) on a mean grey background (32 cd/m^2). As no qualitative differences between light and dark bars were noted, data for the two stimuli are combined. Each bar was 10° in width moving at 20°/second and separated from the preceding bar by 30° - enabling more than one bar on the screen at any one time. The long axis of the bar was orthogonal to the direction of motion. Visual experiments were generated and controlled using custom written Labview and Matlab code (MathWorks) implemented on a ViSaGe stimulus presenter (Cambridge Research Systems, UK) and delivered via a DLP pico projector (Optoma). Functional imaging was performed using an LSM 710 confocal microscope equipped with a spectral detection scan head and a 20×/1.0 NA water-immersion objective (Carl Zeiss). Functional time-series of visually evoked SyGCaMP3 responses in RGCs were acquired at a rate of 4.1 Hz and 0.415 x 0.415 µm resolution, (256 x 256 pixels) and 1 A.U. pinhole aperture at three depths separated by 2 µm. The average diameter of a presynaptic bouton in zebrafish RGCs is ~0.8 µm (Meyer and Smith, 2006). Thus, the physical X-Y dimensions of voxels are below that of a typical presynaptic bouton. Imaging of visually evoked GCaMP

responses in tectal cells were acquired at a rate of 3.2 Hz and 0.66 x 0.66 µm resolution (256 x 256 pixels) and 1 AU pinhole at three depths separated by 7 µm (in the case of pan-neuronal GCaMP5G expression) and at the level of the cell body (in the case of single GCaMP6F expressing tectal cells). Excitation was provided by 488 nm multi-line laser. Occasionally we saw responses in isolated voxels at the onset of the laser, but generally no responses were observed unless visual stimuli were applied. Each orientation was presented once (8 seconds - 4 cycles) in a pseudo-random order unique to each slice in every animal imaged; each inter-epoch interval was 20 seconds to enable GCaMP signals to return to baseline and provide enough baseline to determine the knot for cubic spline interpolation of the baseline signals (see next section). A blank screen null condition of 8 seconds was also interleaved.

Voxel-wise analysis

The confocal time-series were pre-processed prior to analysis as follows: time-series images from each experiment were corrected for motion with a rigid-body algorithm (spm8 - www.fil.ion.ucl.ac.uk/spm); median filtered with a kernel size of 1 voxel (0.415 µm) to remove dark and shot noise; spatially smoothed with a 2D Gaussian kernel = 2 voxels (0.83 µm) to improve signal-to-noise and; low-frequency drifts in baseline corrected using a cubic-spline algorithm extrapolating between knots averaged from 5 seconds of the inter-epoch interval data (~21-22 time-frames). Reference anatomical images were derived from the average of images at this stage of pre-processing. Normalized signal intensity changes (% ΔF/F) were calculated on the detrended time-series at each voxel and the integral response over the epoch-interval calculated to provide a single response metric of each presented orientation. Briefly, a threshold for each voxel within an acquisition image was determined from the normalized signal intensity (% ΔF/F) changes during the inter-epoch intervals and null condition, threshold = 5× standard deviations. All voxels that were suprathreshold within at least two visually presentation epochs were regarded as visually responsive and analyzed for direction- and orientation-selectivity. Direction- and orientation-selective indices (DSI and OSI) (Niell and Stryker, 2008), based on fitted von-Mises profiles (Swindale, 1998), were calculated together with an estimate for their goodness of fit - R^2 (Lowe et al., 2013). To minimize cross talk and overfitting associated with DSI and OSI metrics, a stringent approach was undertaken. For a voxel to be regarded as DS mutually exclusive criteria were used: DS if DSI > 0.5 and OSI < 0.5. The goodness of fit for DSI and OSI, respectively, had to be greater than 0.8; thus, the fitted curves explained at least 80% of the integral responses. The fitted curves used to estimate DSI provided an estimate of the preferred direction of motion from the center of the fitted curve. For automatic segmentation of tectal cells (Hunter et al., 2013), DS voxels were spatially aggregated into islands of like responses whose spatial extent was 10-50 voxels (~7-33 µm^2 – the approximate size limits of a cell soma at this age). The median preferred angle of all constituent voxels was taken as the preferred angle of the cluster.

Identifying functional subtypes of DS-RGC and tectal cells

To derive the number of subtypes of DS-RGCs and tectal cells, grouped cumulative histograms of the preferred direction of motion were constructed from the calculated preferred angles. Multiple von-Mises distributions were summed and fitted to the

cumulative histograms using a multidimensional constrained nonlinear minimization approach, with peak-center, height, concentration, and base height being free dimensions. In all cases, the residuals of the fitted summed distributions were inspected for missed coherent populations. Three and four individual distributions were summed for the RGC and tectal cell cumulative histograms respectively. In both cases no coherent residuals remained. Populations of DS functional subtypes were determined by band-limiting responsive voxels or cells to those within ± 2× the bandwidth of each fitted von-Mises-derived population peak.

Constructing standard tectal neuropil space

Composite maps of direction-selective responses were generated by rigid-body co-registration of the pre-processed derived reference anatomical images to the middle slice of an arbitrarily chosen fish (spm8 - www.fil.ion.ucl.ac.uk/fil/spm). A mean image of the co-registered images was calculated which then became a template that represented the anatomy of the group unbiased by any individual. All slices from all animals were then finally co-registered to this template. Great care was taken to acquire approximately the same slice in the tectal neuropil in all animals by prior imaging the whole neuropil and finding its dorsal and caudal poles. The translations for each imaged slice to the template atlas slice were applied to the threshold direction-selective maps. Individual maps were constructed by summing the incidence of DS functional subtypes across all slices. Composite maps for direction-selective voxels were then derived by color-coding for each respective subpopulation. Rotating the template and composite maps enabled these maps to be collapsed into summary line-plots using the Plot_Profile tool in ImageJ (NIH).

Morphological analysis of tectal cells

Electroporated GCaMP6F- and tdTomato-positive tectal cells were imaged as above. Excitation was 488 nm (for GCaMP6F) or 543 nm (for tdTomato). Image stacks of labeled cells were captured at a 0.33 x 0.33 µm resolution, (512 x 512 pixels) and 1 A.U. pinhole aperture with 0.7 µm sectioning z-interval. Datasets were opened in ImageJ2 (NIH) and tectal arbors were traced using the Simple_Neurite_Tracer plugin. Neurite traces were then further analyzed in ImageJ (NIH) to acquire their morphological parameters: total arbor length, distal arbor A-P span, distance from skin, and distal arbor laminar extent. To obtain the last two parameters the cells were reconstructed in three dimensions and rotated to obtain an orthogonal view. A rectangular region of interest was drawn across a relatively straight stretch of the tectal neuropil to cover the entire neurite. The Plot_Profile tool in ImageJ (NIH) was applied to the rectangle to calculate the fluorescence intensity across tectal depth. Line plots generated were normalized and analyzed using the Prism 5 (GraphPad) 'Smooth' and 'Area Under Curve' functions to measure distance from skin and distal arbor laminar extent (Figure S7A). Distance from skin and distal arbor laminar extent were based on the maximum width of the normalized line plot - a method previously used to characterize tectal arbor morphology (Robles et al., 2011). k-means clustering was performed using the above parameters. For cluster analysis, each data parameter was standardized by diving each value with the population mean. The mean silhouette value of 1-6 clusters was calculated to determine the optimum number of clusters.

DiI/DiD labeling

Retinal projections were anterogradely labeled by mounting paraformaldehyde-fixed larval zebrafish in 1% low melting point agarose (Sigma), and pressure-injecting a 10 mg/ml ethanol solution of the carbocyanine dyes DiI or DiD (Biotium) between the lens and neural retina to label the entire RGC layer. After an overnight diffusion period, DiI/DiD labeled axons were imaged as above. Excitation was 514 nm (for DiI) and 633 nm (for DiD). Image stacks were captured at a 1.18 x 1.18 µm resolution, (512 x 512 pixels) and 1 A.U. pinhole aperture with a 2 µm sectioning z-interval.

Analysis of tectal proliferation and differentiation

To analyze the degree of proliferation and differentiation in the tectum, whole-mount immunohistochemistry was performed on 48 hpf larvae as previously described (Hunter et al., 2011). Anti-PH-3 (Millipore) and anti-HuC/D (Life Technologies) antibodies were used at 1:350 and 1:100 respectively and the detection of primary antibodies was carried out using Alexa Fluor 488, 546 goat anti-rabbit or anti-mouse conjugates (1:500, Life Technologies). TO-PRO-3 (Life Technologies) was used at 1:1000 to stain cell nuclei. Confocal imaging was performed as above. Image stacks were captured at a 0.27 x 0.27 µm resolution, (1024 x 1024 pixels) and 1 A.U. pinhole aperture at several depths separated by 3 µm. The amount of proliferation and differentiation in the tectum was calculated by scoring the number of mitotic ($PH-3^+$) and postmitotic (HuC/D^+) cells, respectively, per tectal hemisphere (in a middle dorso-ventral section of the tectum).

Statistical analyses

Kolmogorov-Smirnov, D’Agostino and Pearson, and Shapiro-Wilk tests followed by an unpaired t-test or Mann Whitney test, as appropriate, were performed. For multiple comparisons, a Kruskal-Wallis and Dunn's multiple comparison test was used. The criterion for statistical significance was set at $p < 0.05$ and results are represented as mean ± SEM. All analyses were performed using Prism 5 (GraphPad).

Supplemental References

Ahrens, M.B., Orger, M.B., Robson, D.N., Li, J.M., and Keller, P.J. (2013). Whole-brain functional imaging at cellular resolution using light-sheet microscopy. Nature methods *10*, 413-420.

Ben Fredj, N., Hammond, S., Otsuna, H., Chien, C.B., Burrone, J., and Meyer, M.P. (2010). Synaptic activity and activity-dependent competition regulates axon arbor maturation, growth arrest, and territory in the retinotectal projection. J Neurosci *30*, 10939-10951.

Bonkowsky, J.L., Wang, X., Fujimoto, E., Lee, J.E., Chien, C.B., and Dorsky, R.I. (2008). Domain-specific regulation of foxP2 CNS expression by lef1. BMC developmental biology *8*, 103.

Chen, T.W., Wardill, T.J., Sun, Y., Pulver, S.R., Renninger, S.L., Baohan, A., Schreiter, E.R., Kerr, R.A., Orger, M.B., Jayaraman, V.*, et al.* (2013). Ultrasensitive fluorescent proteins for imaging neuronal activity. Nature *499*, 295-300.

Fricke, C., Lee, J.S., Geiger-Rudolph, S., Bonhoeffer, F., and Chien, C.B. (2001). astray, a zebrafish roundabout homolog required for retinal axon guidance. Science *292*, 507-510.

Hoegler, K.J., and Horne, J.H. (2010). Targeting the zebrafish optic tectum using in vivo electroporation. Cold Spring Harbor protocols *2010*, pdb prot5463.

Hunter, P.R., Lowe, A.S., Thompson, I.D., and Meyer, M.P. (2013). Emergent properties of the optic tectum revealed by population analysis of direction and orientation selectivity. J Neurosci *33*, 13940-13945.

Hunter, P.R., Nikolaou, N., Odermatt, B., Williams, P.R., Drescher, U., and Meyer, M.P. (2011). Localization of Cadm2a and Cadm3 proteins during development of the zebrafish nervous system. J Comp Neurol *519*, 2252-2270.

Lister, J.A., Robertson, C.P., Lepage, T., Johnson, S.L., and Raible, D.W. (1999). nacre encodes a zebrafish microphthalmia-related protein that regulates neural-crest-derived pigment cell fate. Development *126*, 3757-3767.

Lowe, A.S., Nikolaou, N., Hunter, P.R., Thompson, I.D., and Meyer, M.P. (2013). A Systems-Based Dissection of Retinal Inputs to the Zebrafish Tectum Reveals Different Rules for Different Functional Classes during Development. J Neurosci *33*, 13946-13956.

Meyer, M.P., and Smith, S.J. (2006). Evidence from in vivo imaging that synaptogenesis guides the growth and branching of axonal arbors by two distinct mechanisms. J Neurosci *26*, 3604-3614.

Niell, C.M., and Stryker, M.P. (2008). Highly selective receptive fields in mouse visual cortex. J Neurosci *28*, 7520-7536.

Nikolaou, N., Lowe, A.S., Walker, A.S., Abbas, F., Hunter, P.R., Thompson, I.D., and Meyer, M.P. (2012). Parametric functional maps of visual inputs to the tectum. Neuron *76*, 317-324.

Picker, A., Brennan, C., Reifers, F., Clarke, J.D., Holder, N., and Brand, M. (1999). Requirement for the zebrafish mid-hindbrain boundary in midbrain polarisation, mapping and confinement of the retinotectal projection. Development *126*, 2967-2978.

Robles, E., Smith, S.J., and Baier, H. (2011). Characterization of genetically targeted neuron types in the zebrafish optic tectum. Frontiers in neural circuits *5*, 1.

Swindale, N.V. (1998). Orientation tuning curves: empirical description and estimation of parameters. Biological cybernetics *78*, 45-56.

Villefranc, J.A., Amigo, J., and Lawson, N.D. (2007). Gateway compatible vectors for analysis of gene function in the zebrafish. Developmental dynamics : an official publication of the American Association of Anatomists *236*, 3077-3087.
